# Identification of 22 susceptibility loci associated with testicular germ cell tumors

**DOI:** 10.1038/s41467-021-24334-y

**Published:** 2021-07-23

**Authors:** John Pluta, Louise C. Pyle, Kevin T. Nead, Rona Wilf, Mingyao Li, Nandita Mitra, Benita Weathers, Kurt D’Andrea, Kristian Almstrup, Lynn Anson-Cartwright, Javier Benitez, Christopher D. Brown, Stephen Chanock, Chu Chen, Victoria K. Cortessis, Alberto Ferlin, Carlo Foresta, Marija Gamulin, Jourik A. Gietema, Chiara Grasso, Mark H. Greene, Tom Grotmol, Robert J. Hamilton, Trine B. Haugen, Russ Hauser, Michelle A. T. Hildebrandt, Matthew E. Johnson, Robert Karlsson, Lambertus A. Kiemeney, Davor Lessel, Ragnhild A. Lothe, Jennifer T. Loud, Chey Loveday, Paloma Martin-Gimeno, Coby Meijer, Jérémie Nsengimana, David I. Quinn, Thorunn Rafnar, Shweta Ramdas, Lorenzo Richiardi, Rolf I. Skotheim, Kari Stefansson, Clare Turnbull, David J. Vaughn, Fredrik Wiklund, Xifeng Wu, Daphne Yang, Tongzhang Zheng, Andrew D. Wells, Struan F. A. Grant, Ewa Rajpert-De Meyts, Stephen M. Schwartz, D. Timothy Bishop, Katherine A. McGlynn, Peter A. Kanetsky, Katherine L. Nathanson, The Testicular Cancer Consortium, Christian Kubisch

**Affiliations:** 1 Division of Translational Medicine and Human Genetics, Department of Medicine, Perelman School of Medicine, University of Pennsylvania, Philadelphia, PA USA; 2 Division of Human Genetics, Department of Pediatrics, Children’s Hospital of Philadelphia, Philadelphia, PA USA; 3 Department of Radiation Oncology, Perelman School of Medicine, University of Pennsylvania, Philadelphia, PA USA; 4 Department of Biostatistics, Epidemiology and Informatics, Perelman School of Medicine, University of Pennsylvania, Philadelphia, PA USA; 5 Department of Growth and Reproduction, Rigshospitalet, Copenhagen, Denmark; 6 Department of Surgery (Urology), University of Toronto and The Princess Margaret Cancer Centre, Toronto, ON Canada; 7 Human Genetics Group, Spanish National Cancer Centre (CNIO), Madrid, Spain; 8 Department of Genetics, Perelman School of Medicine, University of Pennsylvania, Philadelphia, PA USA; 9 Division of Cancer Epidemiology and Genetics, Clinical Genetics Branch, National Cancer Institute, Bethesda, MD USA; 10 Program in Epidemiology, Fred Hutchinson Cancer Research Center; Department of Epidemiology, University of Washington, Seattle, WA USA; 11 Departments of Preventive Medicine and Obstetrics and Gynecology, Keck School of Medicine at the University of Southern California, Los Angeles, CA USA; 12 Unit of Endocrinology and Metabolism, Department of Clinical and Experimental Sciences, University of Brescia, Brescia, Italy; 13 Unit of Andrology and Reproductive Medicine, Department of Medicine, University of Padova, Padova, Italy; 14 Department of Oncology, Division of Medical Oncology, University Hospital Centre Zagreb, University of Zagreb School of Medicine, Zagreb, Croatia; 15 Department of Medical Oncology, University Medical Center Groningen, University of Groningen, Groningen, Netherlands; 16 Cancer Epidemiology Unit, Department of Medical Sciences, University of Turin and CPO-Piemonte, Turin, Italy; 17 Department of Research, Cancer Registry of Norway, Oslo, Norway; 18 Faculty of Health Sciences, OsloMet—Oslo Metropolitan University, Oslo, Norway; 19 Department of Environmental Health, Department of Epidemiology, Harvard T.H. Chan School of Public Health, Boston, MA USA; 20 Department of Epidemiology, University of Texas MD Anderson Cancer Center, Houston, TX USA; 21 Center for Spatial and Functional Genomics, Children’s Hospital of Philadelphia, Philadelphia, PA USA; 22 Department of Medical Epidemiology and Biostatistics, Karolinska Institutet, Stockholm, Sweden; 23 Radboud University Medical Center, Nijmegen, Netherlands; 24 Institute of Human Genetics, University Medical Center Hamburg-Eppendorf, Hamburg, Germany; 25 Department of Molecular Oncology, Institute for Cancer Research, Oslo University Hospital-Radiumhospitalet, Oslo, Norway; 26 Institute for Clinical Medicine, Faculty of Medicine, University of Oslo, Oslo, Norway; 27 Division of Genetics & Epidemiology, The Institute of Cancer Research, London, UK; 28 Biostatistics Research Group, Population Health Sciences Institute, Faculty of Medical Sciences, Newcastle University, Newcastle, UK; 29 Division of Oncology, Keck School of Medicine at the University of Southern California, Los Angeles, CA USA; 30 deCODE Genetics/Amgen, Reykjavik, Iceland; 31 Department of Informatics, Faculty of Mathematics and Natural Sciences, University of Oslo, Oslo, Norway; 32 William Harvey Research Institute, Queen Mary University, London, UK; 33 Division of Hematology and Oncology, Department of Medicine, Perelman School of Medicine, University of Pennsylvania, Philadelphia, PA USA; 34 Abramson Cancer Center, Perelman School of Medicine, University of Pennsylvania, Philadelphia, PA USA; 35 School of Public Health, Zhejiang University, Zhejiang, China; 36 Department of Epidemiology, Brown School of Public Health, Brown University, Providence, RI USA; 37 Department of Pathology and Laboratory Medicine, Perelman School of Medicine, University of Pennsylvania, Philadelphia, PA USA; 38 Department of Haematology and Immunology, Leeds Institute of Medical Research at St James’s, University of Leeds, Leeds, UK; 39 Department of Cancer Epidemiology, H. Lee Moffitt Cancer Center and Research Institute, Tampa, FL USA

**Keywords:** Cancer genetics, Testicular cancer, Genetic association study

## Abstract

Testicular germ cell tumors (TGCT) are the most common tumor in young white men and have a high heritability. In this study, the international Testicular Cancer Consortium assemble 10,156 and 179,683 men with and without TGCT, respectively, for a genome-wide association study. This meta-analysis identifies 22 TGCT susceptibility loci, bringing the total to 78, which account for 44% of disease heritability. Men with a polygenic risk score (PRS) in the 95^th^ percentile have a 6.8-fold increased risk of TGCT compared to men with median scores. Among men with independent TGCT risk factors such as cryptorchidism, the PRS may guide screening decisions with the goal of reducing treatment-related complications causing long-term morbidity in survivors. These findings emphasize the interconnected nature of two known pathways that promote TGCT susceptibility: male germ cell development within its somatic niche and regulation of chromosomal division and structure, and implicate an additional biological pathway, mRNA translation.

## Abstract (web summary)

Testicular germ cell tumors are highly heritable, and the authors present the largest genome association study, identifying 22 novel loci, which account for a third of those identified to date. Implicated pathways include male germ cell development and differentiation, and chromosomal segregation.

## Introduction

TGCTs are the most common cancers in young men of European ancestry, and incidence of TGCT has doubled over the past 20 years^1,2^. Family history and cryptorchidism are the strongest known risk factors^3–5^, but no robust environmental risk factors have been identified^1^. Despite the high heritability of TGCT, estimated at 37–49%^6,7^, *CHEK2* is the only moderate penetrance gene in which pathogenic variants have been associated with risk of TGCT^8^.

In contrast, genome-wide association studies (GWAS) have succeeded in identifying common variation associated with TGCT susceptibility^9–21^. Most risk variants map to loci containing genes encoding proteins implicated in critical pathways for male germ cell development, chromosomal segregation, sex determination, and DNA maintenance. Biologically these findings complement the current understanding of disease pathogenesis involving in utero transformation of fetal germ cells into germ cell neoplasia in situ (GCNIS), the common precursor of TGCT^22,23^.

To gain further insight into the genetic underpinnings of TGCT, the Testicular Cancer Consortium (TECAC) present results from a large meta-analysis of 10,156 men with TGCT and 179,683 men without TGCT that combined summary data from numerous extant TGCT GWAS and de novo genotyping from men with and without TGCT. We identify 22 independent loci for TGCT (*P* < 5 × 10^−8^), many of which map to genes that encode proteins in pathways related to male germ cell development, sex determination and chromosomal segregation, as well as mRNA translation. Polygenic risk score (PRS) analysis of all 78 identified risk loci to date reveals a 6.8-fold increase in TGCT risk for men in the top 5% of PRS score compared to those at the median.

## Results

Our meta-analysis incorporated estimates from our published TGCT analysis^9^, genotyping data from deCODE genetics^24^ and the UK Biobank^25^, and summary statistics from genotypes collected from 14 studies collaborating as part of the Testicular Cancer Consortium (TECAC) (Supplementary Tables 1, 2; Supplementary Methods). Initial findings were extended by incorporating results from targeted genotyping of 1039 men with TGCT and 1398 men without TGCT (Supplementary Tables 3, 4).

### GWAS meta-analysis of TGCT

**Fig. 1:**
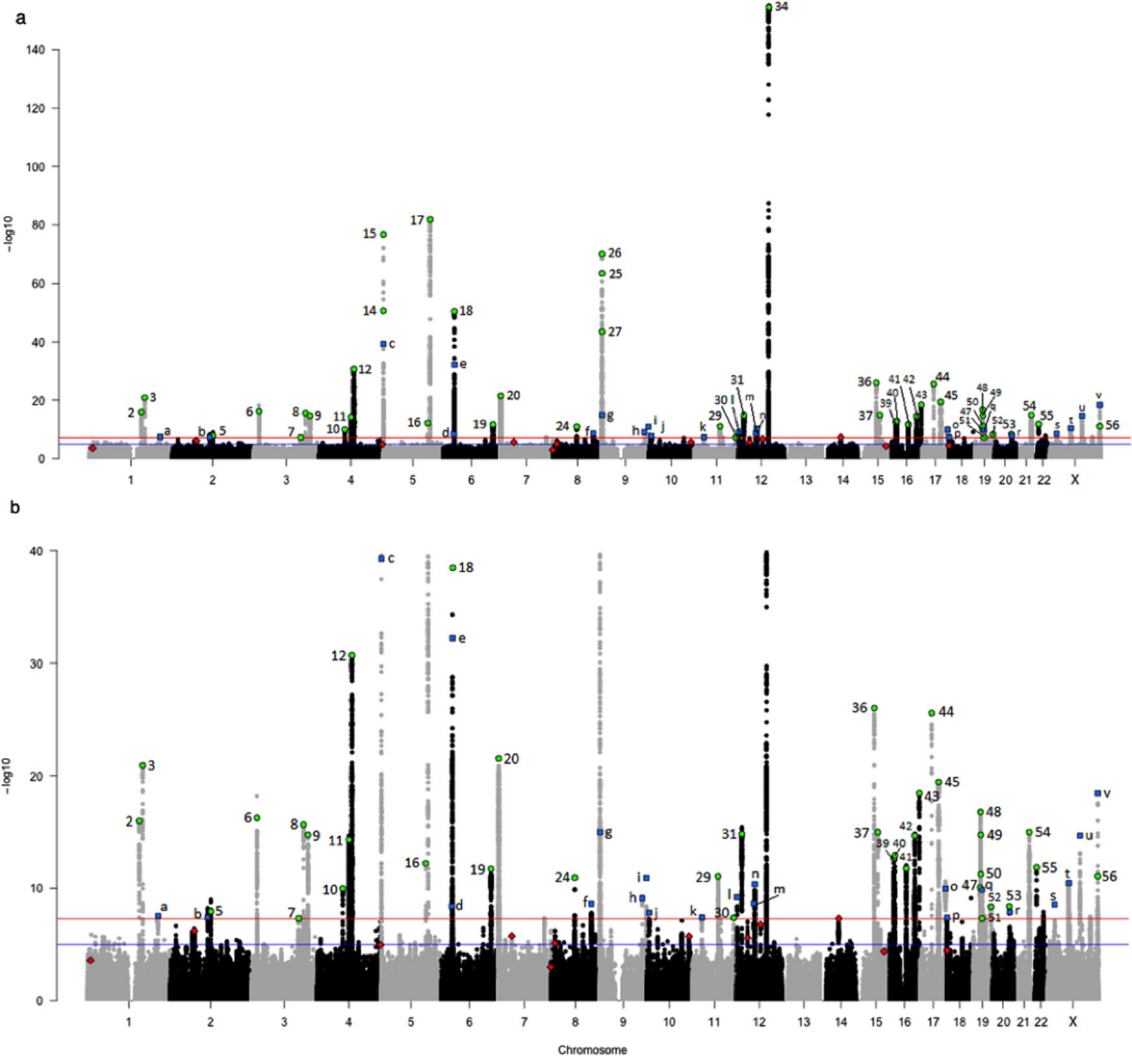
Manhattan plots of markers associated with TGCT risk. Novel markers identified in the current meta-analysis are shown as blue squares () with lowercase letters corresponding to column 1 of Table 1. Susceptibility markers identified in previous studies that surpassed genome-wide significance (*P* ≤ 1 × 10^−8^) in the current meta-analysis are shown as green circles () with numbers corresponding to column 1 of Supplementary Data 2. Susceptibility markers identified in previous studies that failed to attain genome-wide significance (P > 1 × 10^−8^) in the current meta-analysis are shown as red diamonds () with numbers corresponding to column 1 of Supplementary Data 2. **a** Markers are plotted against a full range *y*-axis that incorporates rs4474514 at *KITLG* (*P* = 1.42 × 10^−154^). **b** Markers are plotted against a partial range *y*-axis capped at *P* = 1.42 × 10^−40^ to allow for better visualization and discrimination of most associations.

Our final meta-analysis identified 22 independent susceptibility loci for TGCT (*P* < 5 × 10^−8^) (Table 1, Fig. 1, Supplementary Fig. 1), including four independent signals at previously identified genetic regions (Supplementary Table 5; Supplementary Fig. 2) and four loci on the previously disregarded X chromosome (Supplementary Data 1). The Q–Q plot (Supplementary Fig. 3) and estimated genomic inflation factor (*λ* = 1.03) suggested minimal systematic bias. Only three signals (rs9987332, rs8104804, and rs4898474) showed effect heterogeneity (I^2^ > 50). Forty-four of the 56 previously identified TGCT susceptibility loci^9–21^ replicated at *P* ≤ 5 × 10^−8^ (Supplementary Data 2; Supplementary Data 3). Possible reasons for not replicating all known loci include differences in underlying population substructure, prior overestimation of genetic effect size, effect size heterogeneity, and low r^2^ between the current and previously published loci (Supplementary Data 3). Multiple independent signals were observed at *BAK1* (2), *TKTL1* (2), *TERT* (3), *DMRT1* (4), and the 19p11-p12 (6) region (Table 1; Supplementary Data 2), a complex region containing multiple KRAB-zinc finger proteins (Supplementary Fig. 4). Minimal overlap is present between the 66 novel and replicated independent loci for TGCT and susceptibility loci identified in GWAS of other cancers (Supplementary Data 4). Only four (6%) loci were associated with risk of another cancer type, each with consistency in direction of effect: *BCL2L11* (rs6708784–rs1439287, r^2^ = 0.93) with chronic lymphocytic leukemia, TERT (rs2735940) with colorectal cancer, *HEATR3* (rs2160570–rs10852606, r^2^ = 0.99) with glioblastoma, and *HNF1B* (rs11263762–rs12601991, r^2^ = 1.00) with cancer (pleiotropy).

**Table 1:** Summary information for novel independent TGCT susceptibility loci.

Label	Cytoband	rsID	Location (hg19)	A1/A2	A1 frequency	OR	CI	*P*	Number of genes in region	Adjudicated implicated gene(s)*	Location of signal
a	1p11.1	rs351418	212449403	T/C	0.38	1.11	(1.07, 1.16)	2.85 × 10^−8^	2	***PPP2R5A, PACC1***	Proximal, distal
b	2q13	rs6708784	111927379	G/A	0.50	1.11	(1.07, 1.15)	3.91 × 10^−8^	2	*BCL2L11*	Distal
c	5p15.33	rs7734992	1280128	T/C	0.60	1.32	(1.26, 1.37)	5.17 × 10^−40^	1	TERT*	Intronic
d	6p21.32	rs9469079	32032421	T/C	0.13	1.18	(1.11, 1.25)	3.93 × 10^−9^	1	***TNXB***	Intronic
e	6p21.32	rs141079110	33533625	A/G	0.75	1.23	(1.18, 1.29)	9.39 × 10^−22^	1	BAK1*	Distal
f	8q24.12	rs9987332	120933963	A/G	0.44	1.12	(1.08, 1.16)	2.34 × 10^−9^	1	***DEPTOR***	Intronic
g	9p24.3	rs10976519	779507	G/T	0.42	1.16	(1.12, 1.20)	1.04 × 10^−15^	0	[*DMRT1*]*	Proximal
h	9q33.3	rs10818964	127190340	G/A	0.67	1.13	(1.09, 1.18)	7.92 × 10^−10^	0	Undetermined	–
i	9q34.3	rs28393706	140073294	T/C	0.75	1.18	(1.13, 1.23)	1.20 × 10^−11^	3	***SSNA1***, ***ANAPC2***, ***TPRN***	Intronic, proximal, distal
j	10p14	rs7912968	7534248	C/G	0.38	1.11	(1.07, 1.16)	1.50 × 10^−8^	0	Undetermined	–
k	11p14.1	rs7927974	30351223	G/A	0.29	1.12	(1.08, 1.17)	4.03 × 10^−8^	2	***ARL14EP****, MPPED2*	Intronic, distal
l	12p13.33	rs2887532	1051495	C/T	0.82	1.17	(1.11, 1.23)	6.23 × 10^−10^	1	***RAD52***	Intronic
m	12q13.13	rs12830125	51301431	C/G	0.34	1.14	(1.09, 1.19)	2.18 × 10^−9^	2	Undetermined	–
n	12q13.2	rs35969688	53793209	A/G	0.18	1.17	(1.12, 1.23)	4.32 × 10^−11^	2	***SP1****, AMRH2*	Intronic, proximal
o	17q25.3	rs55779573	76691564	C/T	0.53	1.13	(1.09, 1.17)	1.08 × 10^−10^	2	***CYTH1****, USP36*	Intronic, distal
p	18p11.32	rs2847334	692095	G/A	0.57	1.11	(1.07, 1.16)	4.16 × 10^−8^	1	***ENOSF1***	Intronic
q	19q12	rs8104804	28356614	C/T	0.19	1.17	(1.12, 1.23)	1.38 × 10^−10^	1	***LOC101927151***	Intronic
r	20q13.2	rs6068588	52197366	A/C	0.12	1.18	(1.11, 1.25)	1.32 × 10^−8^	2	***ZNF217***	Intronic
s	Xp22.11	rs72620486	24384181	T/C	0.15	1.14	(1.09, 1.19)	2.74 × 10^−9^	2	*SUPT20HL1*, *PDK3*	5’ UTR, proximal
t	Xq12	rs2335864	66489986	G/A	0.20	1.15	(1.10, 1.20)	3.39 × 10^−11^	0	[*AR*]	Proximal
u	Xq22.1	rs2360670	100432681	A/T	0.54	1.14	(1.10, 1.17)	2.08 × 10^−15^	2	[*CENPI, DPR2*]	Distal, proximal
v	Xq28	rs4898474	153535143	C/T	0.31	1.18	(1.14, 1.22)	3.60 × 10^−19^	2	TKTL1*	Intronic, proximal

Detailed information on the meta-analysis and evaluation of the 22 top signals can be found in Supplementary Data 1, 5, 8 and Supplementary Table 8. Label refers to letter designation in Fig. 1a, b. Novel independent signals in a previously identified gene are indicated with an asterisk (*). Number of genes in the region is defined by r^2^ > 0.80 of the top signal. Associations were tested using a two-sided Wald test on the logistic regression coefficient with an alpha level of 5 × 10^−8^ to account for multiple comparisons. Genes evaluated as highly likely to be the target genes are indicated in **bold** font, and those evaluated as moderately likely are not bold. Genes listed in [brackets] indicate those evaluated as low likelihood to be the target gene. However, one is a well-established TGCT susceptibility gene (*DMRT1*) and the others are located on the X chromosome for which some data is lacking for complete target gene evaluation.

*A1* risk allele, *OR* odds ratio, *CI* confidence interval, *P* P-value for fixed-effects meta-analysis.

Stratified analyses by histology, family history, or cryptorchidism (Supplementary Table 6) did not identify subgroup associations. All 22 susceptibility signals displayed marked differences in minor allele frequency between men of European and African ancestry (Supplementary Table 7) likely explaining some of the observed racial differences in TGCT risk. The 22 identified loci explain 7.0% of father-to-son heritability and 4.7% of heritability among siblings, increasing the overall heritability estimates to 44.0% and 29.1%, respectively.

**Fig. 2:**
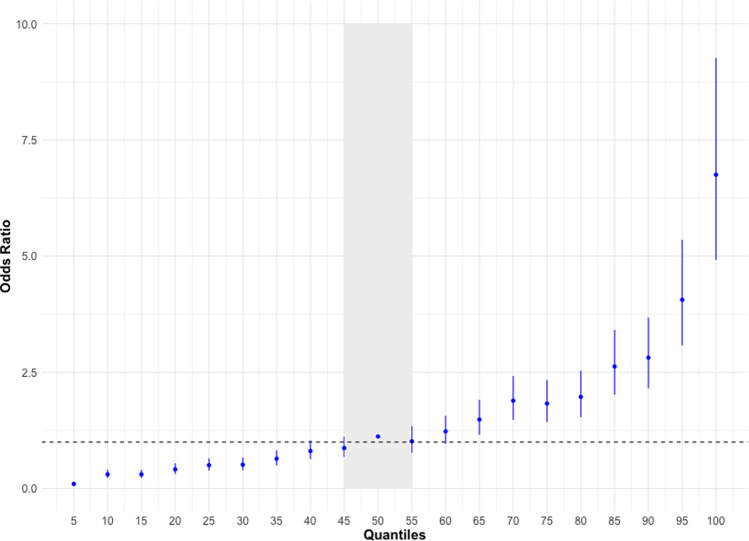
Association of polygenic risk score and TGCT status. Polygenic risk scores (PRS) were calculated for independent samples from *n* = 5602 men with TGCT and 5006 men without disease from a model incorporating the 22 novel and 56 previously identified markers and effect size estimates from the meta-analysis. Odds ratios are relative to the median risk, composed of subjects in 45–55th percentile of PRS. Men in the top 95th percentile had a 6.8-fold increase (odds ratio (OR) = 6.75, 95% confidence interval (CI) 4.92–9.26; *P* = 2.84 × 10^−32^) in risk of developing TGCT compared to men at the 45–55th percentile. Dashed line indicates OR = 1; error bars represent 95% CI.

To generate a polygenic risk score (PRS) for TGCT, we modeled all 78 identified TGCT susceptibility markers, including those that did and did not achieve genome-wide significance in the current study. We found that men in the 95th percentile of PRS had a 6.8-fold increased disease risk (3.4% lifetime risk) compared to men with median scores (Fig. 2). This model identifies men with TGCT with 78.1% accuracy.

### Assessment of credible risk variants (CRV)

We defined a credible risk variant (CRV) as a SNP in strong LD (r^2^ ≥ 0.8) with any of the 66 novel or replicated signals to determine if among the set of 4755 CRVs there are potential functional variants that influence function or expression of the target gene (Supplementary Table 8 and Supplementary Data 5). A total of 108 unique genes were in regions demarcated by the CRVs on the autosomes and X chromosome. Most GWAS have implicated noncoding variation that work through gene regulation (e.g., enhancers, promoters), but coding variation can also influence target gene function. Seventy-three (1.5%) CRVs were located in coding regions; 34 (0.7%) were synonymous and 39 (0.8%) were missense variants (Supplementary Data 6). None were predicted to be pathogenic using REVEL and VEST4^26,27^. Seven (0.1%) CRVs were annotated at a splice site; but only one, rs1060604 at *PMF1* was predicted to influence splicing^28^. These results align with those from other GWAS and support that most susceptibility functional variants affect the regulation of target genes rather than directly altering gene function.

### Inference of autosomal genes associated with TGCT

**Fig. 3:**
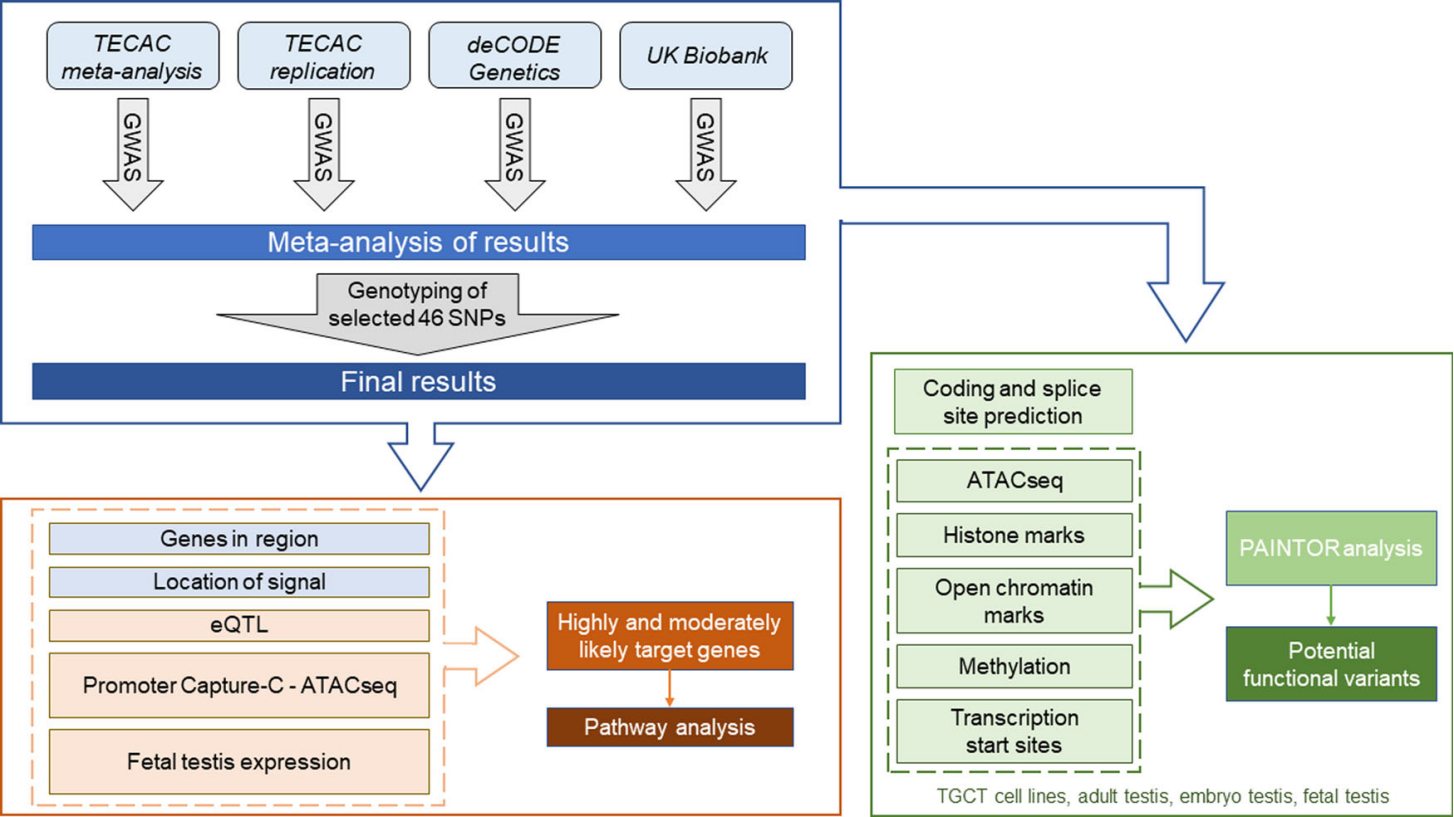
Flow diagram for gene and functional variant inference. Highly and moderately likely target genes were determined by evaluating information derived from GWAS results (blue) and external data sources (orange) including eQTL and promoter Capture-C analyses, and gene expression in fetal testis. To explore potential functional variants, Empirical Bayes modeling in PAINTOR (green) was conducted for all credible risk variants after annotation from multiple publicly available and locally derived data sources.

To identify highly and moderately likely target genes on autosomes, we assessed the gene regions delimited by 4484 CRVs corresponding to 61 top signals (Supplementary Data 5). The total number of target genes evaluated was 108, corresponding to 101 unique genes. As further detailed below, we evaluated (i) the number of genes in the region, (ii) location of the most significantly associated signal, (iii) results from colocalization eQTL analysis^29^, (iv) gene expression in fetal germ cells^30^, and (v) results from promoter Capture-C analysis of the TGCT cell line NT2-D1 (NTERA2)^31,32^ evaluated in conjunction with data from ATAC-seq (Fig. 3). The number of genes in each region ranged from one to eight. For 46 (75%) signals, the gene region included only one or two genes; and for six (10%) signals the gene region encompassed no genes (Supplementary Data 5). Forty-three (70%) of the top signals were in an exon, an intron, or within 10 Kb of a start site (Supplementary Data 5). The colocalization analysis found an eQTL in at least two (non-testis) tissues for 23 (21%) genes, and in testis tissue for 4 (4%) genes (Supplementary Data 5, 7).

Type 2 TGCTs originate from either fetal primordial germ cells or gonocytes and then develop from the noninvasive precursor GCNIS^22^. In the absence of available RNA sequencing data on GCNIS, we used single-cell RNA sequencing data from Li et al.^30^ to evaluate candidate genes for expression in fetal gonads measured at various timepoints. We included male and female germ cells and soma to get a complete picture of the potential expression of genes that may be influencing TGCT development. Transcript levels were categorized as low expression (≤698) for 33 (31%) genes, medium expression (699–2348) for 37 (34%) genes, and high expression (≥2349) for 38 (35%) genes based on tertiles of expression values (Supplementary Fig. 5; Supplementary Data 5). We conducted Assay for Transposase-Accessible Chromatin analysis using sequencing (ATAC-seq) on four TGCT cell lines (Supplementary Data 8, available at https://genome.ucsc.edu/s/jpluta/TECAC2020). The CRVs were significantly enriched in open chromatin regions in all cell lines (EP2102, *P* = 0.0015; NT2-D1 [NTERA2], *P* = 2.63 × 10^−10^; NCCIT, *P* = 4.37 × 10^−8^; TCAM2, *P* = 1.04 × 10^−14^), consistent with a potential effect on gene regulation. We further evaluated data from ATAC-seq in the context of promoter Capture-C data available on one of the cell lines, NT2-D1 to determine whether the promoter region of a target gene demonstrated a connection with a CRV located in an open chromatin region. Seventeen (16%) genes demonstrated these connections (Supplementary Data 5). Connections appearing in two or more cell lines were scored more highly than a connection found in just one cell line. Based on this evaluation of the potential target genes on the autosomes, we classified 37 (37%) genes as highly likely, 25 (25%) genes as moderately likely, and 39 (39%) as unlikely to be associated with TGCT; genes with multiple classification levels were counted in the highest likelihood group (Table 1, Supplementary Data 2, 5).

### Inference of sex chromosome genes associated with TGCT

On the X chromosome, we assessed the gene regions delimited by 271 CRVs corresponding to five top signals (Supplementary Table 8). The total number of unique target genes interrogated was seven. Due to the absence of available eQTL data for X chromosome genes and the lack of expression data in fetal gonads for one target gene, it was not possible to create an equivalent schema to evaluate candidate target genes on the X chromosome. Still, based on our reduced evaluation scheme, one (14%) gene was scored as highly likely and two (29%) genes as moderately likely to be associated with TGCT (Table 1; Supplementary Table 8). However, should eQTL and expression data become available, the four (57%) genes unlikely to be associated with TGCT could be scored as highly or moderately likely (and similarly, the two moderately likely genes could be scored as highly likely); thus, we considered all genes as possible target genes (Supplementary Table 8).

### Testis-specific gene enrichment

Genes selected for enrichment analysis included target genes (*n* = 62) on autosomes that scored moderately or highly likely to be associated with TGCT and all target genes (*n* = 7) on the X chromosome; two of these genes did not have available expression data. There was enrichment of testis-specific expression (*P* = 0.00067) in this gene set with three genes having at least 5-fold greater expression in testis compared to all other tissues (Supplementary Fig. 6). The expression of three other genes was enhanced in testis as indicated by five-fold or greater expression in testis compared to the average in all other tissues.

### Functional assessment of variants by PAINTOR analysis

We also explored potential functional variants determined by PAINTOR, a Baysian approach that combines genetic association, linkage disequilibrium and enriched genomic features (Fig. 3)^33^. We annotated all 4755 CRVs with information from 36 datasets relevant to TGCT, including publicly available data and locally generated data from TGCT cell lines (histone marks, open chromatin marks, transcription factor binding sites, methylation), adult testis (histone marks, open chromatin marks, transcription factor binding sites, methylation, transcription start sites), embryo testis (open chromatin marks), and fetal testis (open chromatin marks) (Supplementary Table 9; Supplementary Fig. 7). PAINTOR analysis prioritized 100 variants as potentially functional, the majority of which had high posterior probabilities (≥95%); four (4%) variants had a posterior probability between 90 and 95%, and only one (1%) fell below 90%^34^ (Supplementary Data 9). Potentially functional variants were found for 57 (86%) of the 66 top signals. Two top signals, rs55873183 in *DMRT1* and rs17336718 in *TKTL1*, contained only one CRV and thus could not be evaluated by PAINTOR. Most variants identified through the PAINTOR analysis were intronic (67%), one was exonic, and most (20%) of the remainder fell within 10 kb of the target gene start site. Of the 102 variants, 83 (81%) disrupted transcription factor binding sites.

## Discussion

Our meta-analysis has increased the number of susceptibility loci for TGCT by one-third. Men in the 95th percentile of the PRS have a 6.8-fold increased disease risk compared to men at the median PRS (Fig. 2); and these men have a 3.4% lifetime risk as compared to 0.4% in the general population^2^. The PRS for TGCT contains fewer SNPs than those available for most other common cancers, yet with a larger effect. For example, women in the 95th percentile of the PRS for breast cancer (313 SNPs) had a 2.4-fold increased disease risk compared to women at the median PRS^35^. The performance of the PRS derived from TGCT susceptibility loci suggests that men at highest risk of disease can be identified.

**Fig. 4:**
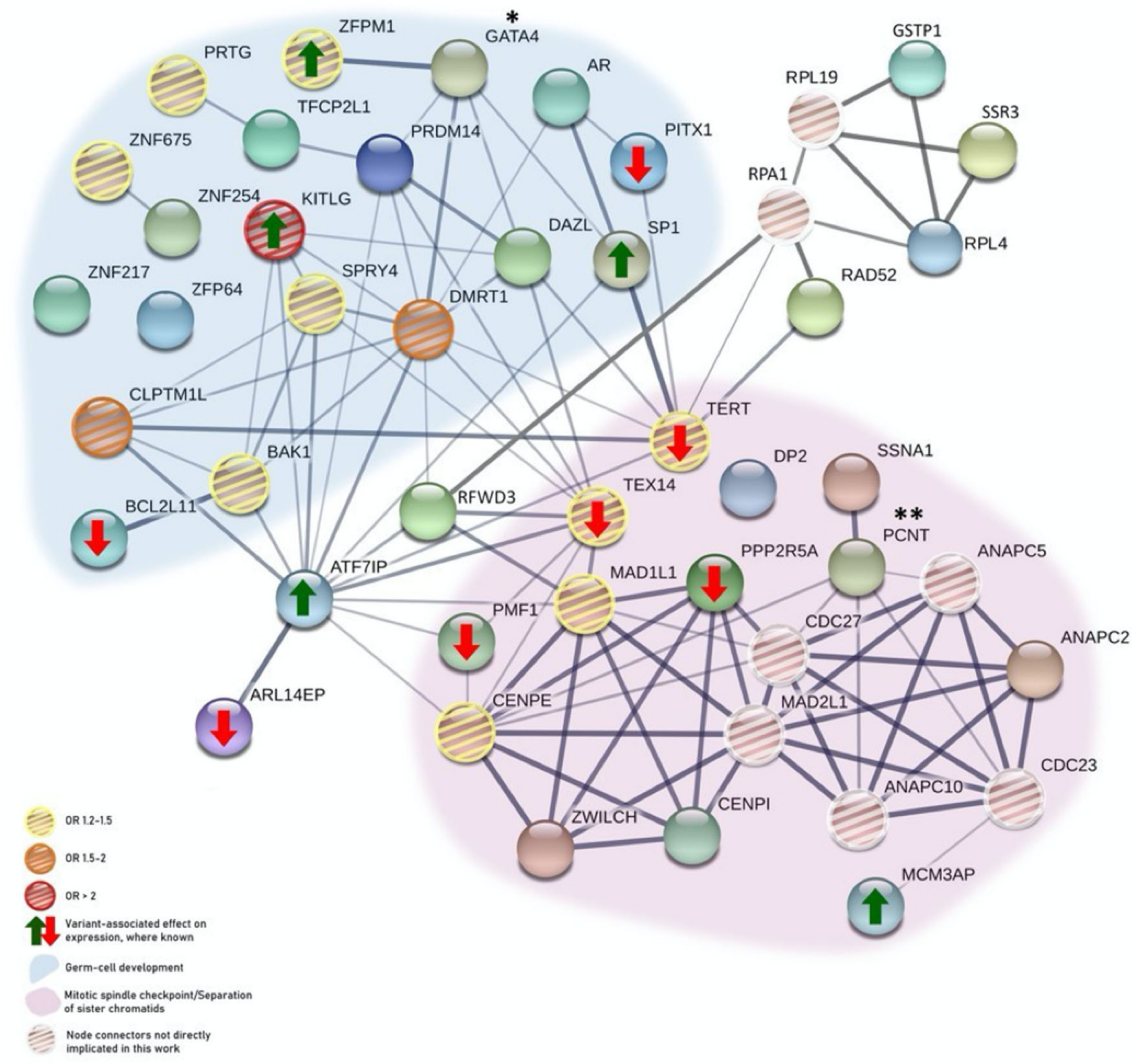
Interaction of proteins in the germ cell development and chromosomal segregation pathways. A protein–protein interaction network for the germ cell development and chromosomal segregation pathways was created using STRING (string-db.org). Proteins encoded by genes implicated as associated with TGCT susceptibility in these pathways are shown, and line weights indicates the degree of confidence of interaction between any two proteins. *GATA4, a previously identified TGCT susceptibility locus, did not reach genome-wide statistical significance in our current study. **PCNT was evaluated as a low likelihood target gene.

Evaluation of top association signals from our meta-analysis identified 65 target genes that were evaluated as moderately or highly likely to be associated with TGCT. Many of these genes encode proteins that fall into biological pathways relevant to TGCT susceptibility, including those that influence male germ cell specification and migration, sex determination and maturation, and regulation of the mitotic cell (HSA-69618, FDR 8.5 × 10^−5^; Fig. 4). For several target genes, findings from murine models support their direct role in the development of TGCT or TGCT-related phenotypes.

Deletion variants at the Steel locus (*Sl*) on the murine 129/Sv background are associated with increased incidence of TGCT; and the etiological gene has been demonstrated to be *Kitl*^36,37^. *KITLG* rs4474514 is the most statistically significant signal in our meta-analysis with a per-allele odds ratio over 2.0. Multiple other target genes implicated by top association signals influence male germ cell development in the mouse. *Prdm14* is critical for the specification of primordial germ cells from somatic cells, participating in the reacquisition of potential pluripotency and successful epigenetic reprogramming^38^. The identified region on 12q13.2 contains two candidate target genes, *SP1* and *AMHR2* (Supplementary Fig. 1n). SP1 is a transcription factor that regulates cellular processes, including inhibition of mouse embryonic stem cell differentiation^39^. eQTL analysis suggest that the potential functional variant is associated with SP1 upregulation, thus similarly favoring developmental arrest by maintaining fetal germ cells in a relatively dedifferentiated state. AMHR2 is the receptor for anti-Mullerian hormone (AMH) which, in addition to testosterone (and hence involvement also of AR), results in male sex differentiation, preventing the development of Mullerian ducts into the uterus and fallopian tubes^40^. In the Japanese rice fish (medaka), knockout of *amrh2* is associated with sex reversal and excessive proliferation of germ cells^41^.

Although we did not define *AR* as a moderately or highly likely target gene due to the lack of available data to inform eQTL analysis or the evaluation of gene expression in fetal testis, the top marker at Xq12 suggests that *AR* may be involved in the etiology of TGCT. Disruption of *AR* leads to androgen insensitivity syndrome and partial sex reversal, depending on the degree of disruption^42^. Furthermore, high linkage disequilibrium (r^2^ = 1) exists between the *AR* locus and variants associated with a decrease in male-pattern baldness^43^ (Supplementary Data 4), a phenotype previously associated with risk of TGCT^44^. Immunohistochemical investigations also identified AR protein to be present in 40–50% of seminoma and GCNIS samples^45^. Further evaluation of this gene is warranted, results from which may further support the long-held hypothesis that a relative decrease in androgen compared to the overall population contributes to risk of TGCT^46^.

We identified a fourth independent susceptibility allele at *DMRT1*, which plays a critical role in sex determination and maintenance of the male somatic niche^47^. Expression of *DMRT1* is enriched in testis tissue. Loss of *Dmrt1* on the murine 129/Sv background leads to an over 90% incidence of testicular teratomas, due to a lack of ability to silence regulators of pluripotency^48,49^. Knockout of *Dazl*, a master transcriptional regulator essential for spermatogenesis, causes spontaneous gonadal teratomas, likely due to prolonged expression of pluripotency genes^50,51^. Expression of *DAZL* is also enriched in testis tissue.

*BAK1* and *BCL2L11* are both members of the BCL-2 family, which together tightly regulate the mitochondrial apoptotic response to either facilitate or prevent cell death depending upon intercellular stimuli^52^. Bak (*BAK1*) is a pro-apoptotic effector of mitochondrial outer membrane permeabilization, which allows release of cytochrome C and other apoptogenic factors leading to cell death^53^. Bim (*BCL2L11*) is a pro-apoptotic BH3-only protein that can activate Bak, but preferentially activates pro-apoptotic effector Bax^54,55^. Interestingly in mouse models, Bim and Bik cooperate to initiate early germ cell apoptosis in a biological pathway that appears to require Bax, but not Bak^56^. Bax also controls apoptosis of fetal germ cells during their migration, and in Bax null mice ectopic germ cells with retained primitive markers are observed^57,58^. Further 60% of Nestin^Cre^Bax^fl/fl^Bak^−/−^ mice develop high-grade tumors within the testis that have expression profiles consistent with germ cell tumors^59^. Our eQTL analysis suggests downregulation of BCL2L11, implying improper survival of arrested germ cells and their transformation to pre-GCNIS.

We also identified multiple target genes encoding proteins involved in chromosomal segregation and heterochromatin organization. Inherited alterations in these genes likely contribute to unique hallmarks of TGCT that has one of the highest aneuploidy scores among cancers, characterized by near universal 12p isochromosome or amplification and frequent genome doubling^60,61^. *PPP2R5A*, a Ser/Thr phosphatase enriched at kinetochores and regulates chromosome-spindle interactions^62^, is an implicated target gene. Similar to *AR*, *CENPI* could not be defined as a top ranking target gene because of lack of available data for the X chromosome; but the top signal at Xq22.1 suggests that *CENPI*, a centromere protein and part of the CENPA-NAC (nucleosome-associated) complex responsible for chromosome alignment and segregation and mitotic progression important for gametogenesis^63,64^, may play a role in TGCT risk. At 9q34.3 the 29.5 kb haplotype block (rs28393706) contains two putative effector genes with overlapping promoter regions, *ANAPC2*, an E3 ligase enzyme that promotes metaphase-anaphase transition as part of the anaphase-promoting complex (APC), and *SSNA1* (SS nuclear autoantigen 1), a centrosomal protein regulating the microtubule-severing activity of spastin^65,66^. Six implicated genes (*PMF1*, *PPP2R5A*, *ANAPC2*, *SSNA1*, *TEX14*, and *MCM3AP*) have an eQTL associated with downregulation, consistent with a more permissive phenotype for chromosomal mis-segregation; and expression of *TEX14* is enriched in testis tissue. Further, multiple TGCT-implicated proteins in the chromosomal segregation pathway interact with TGCT-implicated male germ cell development proteins, demonstrating a biological network underlying TGCT susceptibility (Fig. 4).

After pathway analysis of moderately and highly ranking target genes, several were found to encode proteins that interact in mRNA translation, including one of the ribosomal proteins (*RPL4*), translation termination protein eRF3A (*GSTP1*) and translocon-associated protein subunit gamma (TRAP-gamma, encoded by *SSR3*), which is the general ribosomal interactor participating in the co-translational translocation of proteins into the endoplasmic reticulum^67^. Finally, multiple DNA-binding transcription factors are implicated in TGCT susceptibility, including *HNF1B*, *PITX1*, *PKNOX2*, *PRDM14*, *SP1, TFCP2L1*, *ZFPM1*, *ZNF64*, and *ZNF217*. Several are zinc finger proteins (ZNF) (including KRAB-ZNF) critical for proper germ cell development, such as male primordial germ cells specification and epigenetic reprogramming^68^.

Results from our investigation provide further understanding of the genetic architecture of TGCT, enhance comprehension of the biology of male germ cell development, and highlight biological pathways important to TGCT that are not noted in other cancers. Our findings implicate potentially important pathways, including regulation of apoptosis beyond the BAK1-BCL2L11 axis (*AIFM3*, *CLPTM1L*), enzymatic functions (*MPV17L, TKTL1*, and *UCK2*) and several genes involved in actin, cytoskeleton, and microtubule organization (*CYTH1*, *ENOSF1*, *TNXB*, and *ARL14EP*). The latter may contribute to errors in germ cell migration or chromosomal segregation, likely enhancing the dysregulation of genes directing the germ cell-somatic niche interaction during early development (*KITL, DMRT1*).

Our meta-analysis has identified 66 validated susceptibility loci for TGCT. Many of these loci have a stronger effect size than those observed in adult epithelial cancers, which results in a high fraction of explained heritability of TGCT. Many TGCT risk alleles have higher frequencies in men of European compared to African genetic ancestry, concordant with the known difference in disease incidence between these groups. Importantly, we have established a PRS that identifies men at highest risk of disease. This TGCT PRS could be potentially applied in men with other risk factors, such as cryptorchidism or infertility, to be targeted for early detection and disease mitigation.

## Methods

### Data sources

We procured existing data from five genome-wide association studies of TGCT from 3557 men with TGCT and 13,970 without disease^10,12,18,21^ previously published as a meta-analysis^9^ (Supplementary Table 1); from 300 men with TGCT and 151,991 men without disease provided by deCODE genetics (Reykjavik, Iceland); and from 697 men with TGCT and 8716 men without disease available from the UK Biobank. We completed de novo genome-wide genotyping on 5969 men with TGCT and 5261 without disease ascertained through 14 studies (Supplementary Table 2) from Canada (Princess Margaret Hospital, Toronto), Italy (University of Padova, Padova; University of Turin, Turin), Germany (University Medical Center Hamburg, Hamburg), Netherlands (University Medical Center Groningen, Groningen; Radboud University, Nijmegen), Norway (Cancer Registry of Norway, Oslo; Oslo University Hospital; Oslo), Sweden (Karolinska Institutet, Stockholm), United Kingdom (University of Leeds, Leeds), and the United States (Fred Hutchinson Cancer Research Center, Washington; MD Anderson Cancer Center, Texas; University of Pennsylvania, Pennsylvania; University of Southern California, California; Yale University, Connecticut) termed ‘TECAC’. We also completed de novo targeted SNP genotyping on 481 men with TGCT and 376 men without disease from Spain (Spanish National Cancer Research Centre, Spain) and 277 men with TGCT and 289 men without disease from Pennsylvania (University of Pennsylvania) and 281 men with TGCT and 733 men without disease from 14 TECAC centers whose samples failed pre-genome-wide genotyping quality control (Supplementary Table 3).

### Genotyping

TECAC samples were genotyped on the Illumina Infinium HumanCore-24 BeadChip array, which included a genome-wide backbone of 306,670 SNPs plus custom content of 6290 SNPs for a total of 312,960 genetic markers. Custom content of 7118 SNPs passing initial Illumina quality control was composed of 5598 SNPs from our previous meta-analysis with genome-wide significance 1 × 10^−5^ ≥ *P* > 5 × 10^−8^^11^ and 1520 additional SNPs related to testicular cancer and associated phenotypes. Apart from samples from MD Anderson Cancer Center (2.9%), genotyping was centralized at the Center for Applied Genomics (CAG; University of Pennsylvania, Children’s Hospital of Philadelphia, Philadelphia, PA). Following standard quality control, subjects were excluded because of discordant or ambiguous chromosomal sex, relatedness (IBD > 0.1875), excessive heterozygosity (>3 standard deviations from the mean), low genotype call rate (<98%), or non-European genetic ancestry as determined by principal component analysis (PCA). Quality control was performed using PLINK v1.09 (Purcell et al., 2007), and principal components were calculated using EIGENSOFT v6.1.4^69,70^. Subjects were plotted against the first two principal components and genetic clusters were determined by *k*-means clustering; those greater than six standard deviations from the center of the European cluster were removed (*n* = 581; Supplementary Fig. 8). Subjects with missing information on case status were excluded. SNPs were excluded because of low genotype call rate (< 99%), differential missingness by case status (*P* < 0.00001, Fisher’s exact test), differential missingness by DNA source (blood or saliva; *P* < 0.00001, Fisher’s exact test), Hardy-Weinberg equilibrium (*P* < 0.00001, Fisher’s exact test), duplicate physical position, or minor allele frequency < 0.01. To account for potential batch effects, we also removed SNPs with >10% difference in MAF comparing samples genotyped at MD Anderson to the CAG. After quality control, 10,608 individuals and 246,186 SNPs remained. Genome-wide imputation was performed using the Haplotype Reference Consortium Panel r1.1 (HRC)^71^. Phasing (Eagle2 v2.4.1^72^) and imputation (minimac4 v1.0.0^73^) were conducted automatically on the Michigan Imputation Server (https://imputationserver.sph.umich.edu). Imputed SNPs were screened for MAF, HWE, missingness, and imputation quality (INFO > 0.3).

### Targeted genotyping

Based on results from genotyping and imputation (see below, Genotype analysis and meta-analysis), 46 SNPs were brought forward for targeted genotyping; dbSNP was used to confirm SNP details. DNA was isolated from 2500 samples using Agencourt beads system (Beckman-Coulter), quantified on the Spectramax (Molecular Device) reader using Quant-iT™ PicoGreen® dsDNA Assay Kit, and genotyped on a Fluidigm 192.24 Dynamic Array Integrated Fluidic Circuit in the nanofluidic SNP genotyping system, SNPtype assay (Fluidigm Corp., CA), which employs allele-specifically designed fluorescencent (FAM or VIC) primers and a common reverse primer. SNP arrays were thermal cycled (Juno instrument), and the endpoint fluorescent values were measured on Biomark™ system. Final sample genotype calls and quality control were acquired using Fluidigm SNP Genotyping Analysis software. Subjects were removed for excess heterozygosity (>3 standard deviation from the mean) and genotype missingness (≥10%). SNPs were screened for genotype missingness (>2%), differential missingness (*P* < 0.001), and minor allele frequency (<0.01). After quality control, 1039 men with TGCT and 1398 men without disease remained (Supplementary Table 3).

### Genotype analysis and meta-analysis

Logistic regression was used to determine associations between TGCT status and genotype, assuming an additive genetic model. Regression models were implemented in SNPTEST v2.5^74^, and included the first three PCs and a categorical variable representing study center as covariates. Summary statistics from existing genome-wide association studies were combined using a fixed-effects model implemented in METAL (r. 2018-08-28)^75^, with each coefficient estimate weighted by the inverse of its variance (Supplementary Data 1, 3). To account for different coverage of the various reference panels, only SNPs that were present in all studies were considered. Multiallelic variants and SNPs demonstrating study heterogeneity (*P* < 0.001, Cochran’s Q test) were removed. We then selected the 60 top ranking previously unreported SNPs that were strongly associated (*P* < 5 × 10^−6^) with TGCT case status for targeted genotyping. Of these 60, 46 passed in silico and initial quality testing for Fluidigm primer specificity. Each SNP was tested for its association with TGCT, adjusted for study center. Results were combined with study-specific estimates derived from genome-wide genotype data (above) using METAL. Overall summary odds ratios and corresponding 95% confidence intervals were obtained. Associations with *P* ≤ 5 × 10^−8^ were considered statistically significant.

### Validation of imputed genotypes

TECAC subjects with genome-wide genotyping were rank-ordered based on the total number of minor alleles at the 46 SNPs represented on the targeted genotyping panel. We selected the top 500 subjects, assuring at least 10% representation of the minor allele for each SNP, for genotyping on the targeted panel. Two subjects were removed for missingness, and one for excessive heterozygosity, leaving 497 subjects (267 cases, 230 controls). The correlation coefficient between observed genotype on the targeted panel and imputed genotype inferred from genome-wide genotyping for 36 susceptibility loci was calculated. The average concordance was 0.96 (0.93, 0.99) (Supplementary Table 4).

### Independence analysis

For genetic regions with more than one SNP that reached genome-wide significance, we conducted conditional and joint (COJO) multiple-SNP analysis using GCTA v1.26.0^76^ to determine independence of each SNP marker. We used the summary statistics from our meta-analyses and individual-level SNP data from TECAC subjects to estimate pairwise linkage disequilibrium (Supplementary Table 5). For each region of interest, the most significant (i.e., reference) SNP was jointly modeled with each other ‘test’ SNP in the region. If the test SNP retained genome-wide significance in the joint model, it was deemed independent. This procedure was performed iteratively, adding the most highly significant independent SNP to the model at each step, ending when there were no more independent SNPs that reached genome-wide significance. SNPs were further interrogated by visualizing results in LocusZoom v1.4 and custom independence plots written in R.

### Stratified analysis

We conducted analyses stratified by family history of TGCT, tumor subtype (seminoma, nonseminoma, mixed), and cryptorchidism, for those studies and case subjects with available data (Supplementary Table 6). Associations were determined using an analytic pipeline mirroring the main analysis. In the analysis of tumor subtype, SNPs with a MAF < 0.05 were removed as were variants with study heterogeneity exceeding *P* < 0.05 by Cochran Q. In the analyses of family history and cryptorchidism, SNPs with a MAF < 0.05 were removed and only variants for which all study-specific effects were in the same direction were retained; and we did not rely on Cochran Q to test for study heterogeneity because of reduced power to detect differences.

### Heritability

We estimated heritability of a given SNP as the proportion of the total phenotypic variance explained by the SNP. The phenotypic variance can be considered the sum of genetic and environmental effects, which can be approximated from the familial relative risk. We used a derived value of four for the relative risk (RR) for affected fathers and eight for brothers^77^. With the RR represented by *λ*, heritability is then calculated as:

1 $$h=\frac{{\beta }^{2}\ast 2f(1-f)}{\log ({\lambda }^{2})}$$

where β is the estimated log-odds ratio of the SNP, and *f* is the frequency of the effect allele.

### Polygenic risk score

A polygenic risk score (PRS) consisting of the 22 novel and 56 previously identified susceptibility loci was calculated for 5602 men with TGCT and 5006 men without disease (Supplementary Table 2) using PLINK v1.09. The previously published data was only used in the calculation of effect sizes, as raw genotype data were not available, and to avoid bias from chip or batch effects. The number of risk alleles was multiplied by the effect size from the meta-analysis and summed across all risk loci. A lifetime risk of 0.5% for TGCT was assumed, which accounted for the range of risks over the countries included in the current study (e.g., lifetime risks in the United States 0.4%, United Kingdom 0.53%, Netherlands 0.64%, and Denmark 0.82%)^78^. The out-of-sample accuracy of the PRS was determined by leave-one-out cross validation of the area under the receiver-operator curve, which reflects the probability that the PRS can accurately predict TGCT status in a random subject.

### SNP associations with race and other GWAS studies

For the 22 identified loci, the variant frequency of most strongly associated SNP was downloaded from dbSNP (gnomAD—Genomes Accession: PRJNA398795 ID: 398795) for European (SAMN10181265), African (SAMN07488254) and East Asian (SAMN07488251) groups. Comparisons of risk allele frequencies were done using two-tailed Fisher’s Exact test (Supplementary Table 7). To determine associations with other GWAS studies, we used the suite of applications within LDLink^79–81^, using an LD of r^2^ > 0.80 (Supplementary Data 4).

### Credible risk variants (CRVs)

CRVs were defined to include all SNPs with LD of r^2^ ≥ 0.80 of the most strongly associated SNP in each locus, using the European population in the HRC. LD was estimated using GCTA. CRVs were annotated with NCBI’s hg19 RefSeq database using ANNOVAR r. 2019-10-24^82^.

### Colocalization analysis

For each GWAS locus, we used colocalization to find evidence that the GWAS signal at that locus could be explained by an eQTL signal. We used publicly available data from the GTEx consortium for this analysis. GWAS summary statistics were converted from hg37 to hg38 using LiftOver (https://genome.ucsc.edu/cgi-bin/hgLiftOver), resulting in a loss of 1,284,722 variants (6.0%). For each phenotype, colocalization analysis was run in windows across the genome separately for each of the 49 tissues in GTEx v8^83^. We first identified previously defined LD blocks for the genome^84^ with a sentinel SNP at *P* < 5 × 10^−8^, and restricted colocalization analysis to these LD blocks. For each LD block with a sentinel SNP, all genes within 1 Mb of the sentinel SNP (cis-Genes) were identified, and then restricted to those that were identified as eGenes in GTEx v8 (cis-eGenes). For each cis-eGene, colocalization analysis was performed using all variants within 1 Mb of the gene. A significant colocalization^29^ was defined as PP3 + PP4 > 0.8 and PP4/(PP3 + PP4) > 0.9 (Supplementary Data 7).

We and others have shown that colocalization analyses are most informative when performed across a diverse set of tissues and datasets^85^. Although the GTEx data are quite comprehensive, there are varying sample sizes across the 50 sampled tissues and eQTL effects are often shared across multiple tissues^85^. As a result, the power to detect eQTL-GWAS colocalizations varies by tissue, and multi-tissue analyses can discover more informative eQTL-GWAS colocalizations than analyses that rely on a single dataset or tissue. Thus, we do not rely solely on adult testis tissue for identifying eQTLs of interest, especially as it contains multiple tissue types and adult germ cells rather than primordial germ cells, the cells of origin for TGCT.

### ATAC-seq library generation and peak calls

Live cells from the TGCT cell lines were harvested via trypsinization, followed by a series of wash steps. 100,000 cells from each sample were pelleted at 550 × *g* for 5 min at 4 °C. The cell pellet was then resuspended in 50 μl cold lysis buffer (10 mM Tris-HCl, pH 7.4, 10 mM NaCl, 3 mM MgCl2, 0.1% IGEPAL CA-630) and centrifuged immediately at 550 × *g* for 10 min at 4 °C. The nuclei were resuspended in the transposition reaction mix (2× TD Buffer (Illumina Cat #FC-121–1030, Nextera), 2.5 µl Tn5 Transposase (Illumina Cat #FC-121–1030, Nextera), and Nuclease Free H2O) on ice and then incubated for 45 min at 37 °C. The transposed DNA was then purified using the MinElute Kit (Qiagen), eluted with 10.5 μl elution buffer. The transposed DNA was PCR amplified using Nextera primers for 12 cycles to generate each library. The PCR reaction was subsequently cleaned up using AMPureXP beads (Agencourt) and libraries were paired-end sequenced on the Illumina NovaSeq platform. Open chromatin regions were called using the ENCODE ATAC-seq pipeline (https://www.encodeproject.org/atac-seq/), selecting the resulting conservative irreproducible discovery peaks (with all coordinates referring to hg19). Each cell line was evaluated in triplicate. We defined a genomic region open if it had 1 bp overlap with an ATAC-seq peak.

### Cell fixation for chromatin capture

The protocol used for cell fixation was in line with previous methods^86^. NT2-D1 cells were collected and single-cell suspension were made with aliquots of 10 million cells in 10 mL media. Five hundred forty microliters (37%) formaldehyde was added and incubated for 10 min at RT on a platform rocker. The reaction was quenched by adding 1.5 mL 1 M cold glycine (4 °C) for a total volume of 12 mL. Fixed cells were centrifuged at 1000 rpm for 5 min at 4 °C and supernatant removed. The cell pellets were washed in 10 mL cold PBS (4 °C) followed by centrifugation as above. Supernatant was removed and cell pellets were resuspended in 5 mL of cold lysis buffer (10 mM Tris pH8, 10 mM NaCl, 0.2% NP-40 supplemented with protease inhibitor cocktails). Resuspended cells were incubated for 20 min on ice, centrifuged as above, and the lysis buffer removed. Finally, cell pellets were resuspended in 1 mL fresh lysis buffer, transferred to 1.5 mL Eppendorf tubes and snap frozen (ethanol/dry ice or liquid nitrogen). Cells were stored at −80 °C until they were thawed for 3 C library generation.

### 3C library generation and promoter Capture-C

We used standard methods for generation of 3 C libraries^31,32^. For each library, 10^7^ fixed cells were thawed at 37 °C, followed by centrifugation at RT for 5 min at 1845 × *g*. The cell pellet was resuspended in 1 mL of dH2O supplemented with 5 μL 200× protease inhibitor cocktail, incubated on ice for 10 min, then centrifuged. The cell pellet was resuspended to a total volume of 650 μL in dH2O. Fifty microliters of cell suspension was set aside for predigestion QC, and the remaining sample was divided into three tubes. Both predigestion controls and samples underwent a predigestion incubation in a Thermomixer (BenchMark) with the addition of 0.3%SDS, 1× NEB DpnII restriction buffer, and dH2O for 1 h at 37 °C shaking at 1000 rpm. A 1.7% solution of Triton X-100 was added to each tube and shaking was continued for another hour. After predigestion incubation, 10 μl of DpnII (NEB, 50 U/µL) was added to each sample tube only and continued shaking along with predigestion control until the end of the day. An additional 10 µL of DpnII was added to each digestion reaction and digested overnight. The next day, a further 10 µL DpnII was added and continue shaking for another 2–3 h. 100 μL of each digestion reaction was then removed, pooled into one 1.5 mL tube, and set aside for digestion efficiency QC. The remaining samples were heat inactivated incubated at 1000 rpm in a MultiTherm for 20 min at 65 °C to inactivate the DpnII and cooled on ice for 20 additional minutes. Digested samples were ligated with 8 μL of T4 DNA ligase (HC ThermoFisher, 30 U/µL) and 1× ligase buffer at 1000 rpm overnight at 16 °C in a MultiTherm. The next day, an additional 2 µL of T4 DNA ligase was spiked into each sample and incubated for another few hours. The ligated samples were then decrosslinked overnight at 65 °C with Proteinase K (20 mg/mL, Denville Scientific) along with predigestion and digestion control. The following morning, both controls and ligated samples were incubated for 30 min at 37 °C with RNase A (Millipore), followed by phenol/chloroform extraction, ethanol precipitation at −20 °C, the 3 C libraries were centrifuged at 85 × *g* for 45 min at 4 °C to pellet the samples. The controls were centrifuged at 1845 × *g*. The pellets were resuspended in 70% ethanol and centrifuged as described above. The pellets of 3 C libraries and controls were resuspended in 300 and 20 μL dH_2_O, respectively, and stored at −20 °C. Sample concentrations were measured by Qubit. Digestion and ligation efficiencies were assessed by gel electrophoresis on a 0.9% agarose gel and also by quantitative PCR (SYBR green, Thermo Fisher).

The promoter Capture-C approach was designed to leverage the four-cutter restriction enzyme *DpnII* in order to give high-resolution restriction fragments of a median of ~250 bp^31,32^. Custom capture baits were designed using Agilent SureSelect RNA probes targeting both ends of the *DpnII* restriction fragments containing promoters for coding mRNA, noncoding RNA, antisense RNA, snRNA, miRNA, snoRNA, and lincRNA transcripts (UCSC lincRNA transcripts and sno/miRNA under GRCh37/hg19 assembly) totaling 36,691 RNA baited fragments through the genome^86^. In this study, the capture library was reannotated under gencodeV19 at both 1-fragment and 4-fragment resolution and is successful in capturing 89% of all coding genes and 57% of noncoding RNA gene types. The missing coding genes could not be targeted due to duplication or highly repetitive DNA sequences in their promoter regions.

Isolated DNA from 3 C libraries was quantified using a Qubit fluorometer (Life Technologies), and 10 μg of each library was sheared in dH_2_O using a QSonica Q800R to an average fragment size of 350 bp. QSonica settings used were 60% amplitude, 30 s on, 30 s off, 2 min intervals, for a total of five intervals at 4 °C. After shearing, DNA was purified using AMPureXP beads (Agencourt). DNA size was assessed on a Bioanalyzer 2100 using a DNA 1000 Chip (Agilent) and DNA concentration was checked via Qubit. SureSelect XT library prep kits (Agilent) were used to repair DNA ends and for adaptor ligation following the manufacturer protocol. Excess adaptors were removed using AMPureXP beads. Size and concentration were checked by Bioanalyzer using a DNA 1000 Chip and by Qubit fluorometer before hybridization. One microgram of adaptor-ligated library was used as input for the SureSelect XT capture kit using manufacturer protocol and our custom-designed 41 K promoter Capture-C library. The quantity and quality of the captured library was assessed by Bioanalyzer0a high sensitivity DNA Chip and by Qubit fluorometer. SureSelect XT libraries were then paired-end sequenced on 8 lanes of Illumina Hiseq 4000 platform (100 bp read length).

### Analysis of Capture-C data

Quality control of the raw fastq files was performed with FastQC. Paired-end reads were preprocessed with the HiCUP pipeline60, with bowtie2 v2.4.2 as aligner and hg19 as reference genome. Significant promoter interactions at 1-DpnII fragment resolution were called using CHiCAGO v3.12^87^ with default parameters except for binsize which was set to 2500. Significant interactions at 4-DpnII fragment resolution were also called with CHiCAGO using artificial *.baitmap and *.rmap files where DpnII fragments were grouped into four consecutively and using default parameters except for removeAdjacent which was set to False. We define PIR a promoter-interacting region, irrespective of whether it is a baited region or not. The CHiCAGO function peakEnrichment4Features was used to assess enrichment of genomic features in promoter-interacting regions at both 1-fragment and 4-fragment resolution.

### ATAC-seq and high-resolution promoter Capture-C variant to gene mapping

We first identified all proxy SNPs in LD (r^2^ = 0.4) with the sentinel GWAS SNPs using SNiPA v3.4 (https://snipa.helmholtz-muenchen.de/snipa3/) with the following parameters: population = European; genome annotation = Ensembl 87; genotype database = 1000 Genomes Phase 3 v5; and genome assembly = GRCH37/hg19. We then assessed which of these proxy SNPs and which of the gene promoters baited in our Capture-C library resided in an open chromatin region in NT2-D1, by intersecting their genomic positions with those of the ATAC-seq peaks (using the BEDTools function intersectBed with 1 bp overlap). Finally, we exported the chromatin loops linking open proxy SNPs and open gene promoters in the NT2-D1 Capture-C dataset using only the 4-fragment resolution to increase power.

### Scoring of target genes

We devised a scoring system to determine target genes within gene regions demarcated by CRVs based on a published computational pipeline, integrated expression quantitative trait and in silico prediction of GWAS targets (INQUISIT)^34^. Due to the paucity of data available for TGCT, we modified the scoring system such that each gene was scored on (i) the number of genes in the region [2 = one gene; 1 = two or more genes; 0 = no genes]; (ii) location of most significantly associated signal [1 = exonic, intronic, or within ±10 Kb of a gene]; (iii) results from colocalization eQTL analysis^29^ [1 = two or more in non-testis tissue; 0.5 = one in non-testis tissue; 0 = none in non-testis tissue; and +1 = one in testis tissue]; (iv) gene expression in fetal germ cells based on tertiles of expression levels available from Li et al.^30^ [1 = high; 0.5 = medium; 0 = low;] (Supplementary Fig. 6); and (v) results from NT2-D1 promoter Capture-C analysis evaluated in conjunction with data from ATAC-seq in four TGCT cell lines [1 = connection in two or more cell lines; 0.5 = connection in one cell line; 0 = no connections]. Target genes were then categorized a highly likely [score ≥ 3.0), moderately likely (score = 2.0 or 2.5), or unlikely (score ≤ 2.0) to be associated with TGCT.

### Testis-specific gene enrichment

For the set of target autosomal genes that scored moderately or highly likely to be associated with TGCT and all target genes on the X chromosome, we determined tissue-specific gene expression using MAGMA v1.07 as implemented in FUMA v1.3.5^88^ (Supplementary Fig. 5); 67 genes had expression data available in GTeX. Testis-specific enrichment for this gene set was determined using the TissueEnrich v1.10.0 R package^89^. Genes with a minimum of 1 TPM and five-fold or higher expression in testis tissue compared to any other tissues were considered testis-enriched; gene not reaching the definition of testis-enriched, but with a minimum of 1 TPM and five-fold or higher expression in testis tissue compared to the average in all other tissues were considered testis-enhanced.

### PAINTOR analysis

We downloaded 36 unique datasets with information on methylation, open chromatin marks, histone marks, and transcription factor binding sites, i.e., features, in testis tissue or cell lines from ENCODE^90^ (Supplementary Table 9). All CRVs were annotated with these data and with locally derived data from ATAC-seq on four TGCT cell lines (2102EP, TCAM2, NT2-D1, NCCIT); methods described below. For each locus, all features that showed evidence of association (*P* < 0.15) were assessed for independence (r^2^ < 0.4). A likelihood-ratio test was used to determine if independent features yielded a statistically significant improvement in fit over a model without any features (*P* < 0.05). The selected features were entered into an Empirical Bayes model (Probabilistic Annotation Integrator, PAINTOR v3.0^33,91,92^ that was additionally informed by SNP association test statistics and linkage disequilibrium (LD). The model returned the likelihood that a given SNP was functional, for each SNP in the CRV (Supplementary Data 9, Supplementary Fig. 7).

### Transcription factor binding

We annotated all potential causal variants identified by PAINTOR and the two top signals with only one CRV in the region (*n* = 102) with transcription factor binding motifs (Supplementary Data 9). For each allele, we analyzed the matrix values, which also allows a determination of whether the disruption is strong or weak. Analysis was performed using the R package motifbreakerR v2.4.0^93^.

### Reporting summary

Further information on research design is available in the Nature Research Reporting Summary linked to this article.

## Supplementary information

Supplementary Information

Description of Additional Supplementary Files

Supplementary Data 1-10

Reporting Summary

## Data Availability

The meta-analysis data are uploaded under dbGaP phs001349.v1.p1 [https://www.ncbi.nlm.nih.gov/projects/gap/cgi-bin/study.cgi?study_id=phs001307.v1.p1] (Meta-Analysis of Five Genome-Wide Association Studies of TGCT) and the replication data under phs001349.v2.p1 [https://www.ncbi.nlm.nih.gov/projects/gap/cgi-bin/study.cgi?study_id=phs001349.v2.p1] (NCI Testicular Germ Cell Tumors Post GWAS). Summary statistics for the top 10,000 SNPs are available in Supplementary Data 10. UKBiobank data are available to all bona fide researchers upon data access application at http://www.ukbiobank.ac.uk/register-apply/. We obtained them under application number 3071 to Professor D. Timothy Bishop. The Icelandic population WGS data have been deposited at the European Variant Archive under accession code PRJEB15197. Access to the deCODE WGS is restricted due to Icelandic law and the regulations of the Icelandic Data authority, which prohibits the release of individual-level and personally identifying data. Requests for access may be submitted to deCODE directly by contacting B.V.H. (bjarni.halldorsson@decode.is) or K.S. (kstefans@decode.is). Access to these data can be granted only at the facilities of deCODE genetics in Iceland, subject to Icelandic laws regarding data usage. The ATAC-seq data and ENCODE data for PAINTOR analysis are included on a UCSC browser custom track at: https://genome.ucsc.edu/s/jpluta/TECAC2020. ATAC-seq and Capture-C data are uploaded to the Gene Express Ominbus (GEO) under accession number GSE175368. The remaining data are available within the Article, Supplementary Information, or from the authors upon request.

## Code availability

The code used in the analysis of this data is available at: https://github.com/nathanson-lab/TGCT_2021_NatureCommunications. Code used in the analysis is available at 10.5281/zenodo.4630810.

## References

[1] Gurney JK (2019). International trends in the incidence of testicular cancer: lessons from 35 years and 41 countries. Eur. Urol..

[2] Howlader, N. et al. *SEER Cancer Statistics Review, 1975–2017* (National Cancer Institute, 2020).

[3] Fossa SD (2005). Risk of contralateral testicular cancer: a population-based study of 29,515 U.S. men. J. Natl Cancer Inst..

[4] Dieckmann KP, Pichlmeier U (2004). Clinical epidemiology of testicular germ cell tumors. World J. Urol..

[5] Cheng L (2018). Testicular cancer. Nat. Rev. Dis. Prim..

[6] Litchfield K (2015). Quantifying the heritability of testicular germ cell tumour using both population-based and genomic approaches. Sci. Rep..

[7] Mucci LA (2016). Familial risk and heritability of cancer among twins in Nordic Countries. JAMA.

[8] AlDubayan SH (2019). Association of inherited pathogenic variants in checkpoint kinase 2 (CHEK2) with susceptibility to testicular germ cell tumors. JAMA Oncol..

[9] Wang Z (2017). Meta-analysis of five genome-wide association studies identifies multiple new loci associated with testicular germ cell tumor. Nat. Genet.

[10] Kanetsky PA (2011). A second independent locus within DMRT1 is associated with testicular germ cell tumor susceptibility. Hum. Mol. Genet.

[11] Kanetsky PA (2009). Common variation in KITLG and at 5q31.3 predisposes to testicular germ cell cancer. Nat. Genet..

[12] Chung CC (2013). Meta-analysis identifies four new loci associated with testicular germ cell tumor. Nat. Genet..

[13] Schumacher FR (2013). Testicular germ cell tumor susceptibility associated with the UCK2 locus on chromosome 1q23. Hum. Mol. Genet..

[14] Loveday C (2018). Validation of loci at 2q14.2 and 15q21.3 as risk factors for testicular cancer. Oncotarget.

[15] Litchfield K (2017). Identification of 19 new risk loci and potential regulatory mechanisms influencing susceptibility to testicular germ cell tumor. Nat. Genet..

[16] Litchfield K (2015). Multi-stage genome-wide association study identifies new susceptibility locus for testicular germ cell tumour on chromosome 3q25. Hum. Mol. Genet.

[17] Litchfield K (2015). Identification of four new susceptibility loci for testicular germ cell tumour. Nat. Commun..

[18] Ruark E (2013). Identification of nine new susceptibility loci for testicular cancer, including variants near DAZL and PRDM14. Nat. Genet.

[19] Turnbull C (2010). Variants near DMRT1, TERT and ATF7IP are associated with testicular germ cell cancer. Nat. Genet.

[20] Rapley EA (2009). A genome-wide association study of testicular germ cell tumor. Nat. Genet.

[21] Kristiansen W (2015). Two new loci and gene sets related to sex determination and cancer progression are associated with susceptibility to testicular germ cell tumor. Hum. Mol. Genet.

[22] Rajpert-De Meyts E, McGlynn KA, Okamoto K, Jewett MA, Bokemeyer C (2016). Testicular germ cell tumours. Lancet.

[23] Skakkebaek NE, Berthelsen JG, Giwercman A, Muller J (1987). Carcinoma-in-situ of the testis: possible origin from gonocytes and precursor of all types of germ cell tumours except spermatocytoma. Int. J. Androl..

[24] Gudbjartsson DF (2015). Large-scale whole-genome sequencing of the Icelandic population. Nat. Genet..

[25] Bycroft C (2018). The UK Biobank resource with deep phenotyping and genomic data. Nature.

[26] Carter H, Douville C, Stenson PD, Cooper DN, Karchin R (2013). Identifying Mendelian disease genes with the variant effect scoring tool. BMC Genomics.

[27] Ioannidis NM (2016). REVEL: an ensemble method for predicting the pathogenicity of rare missense variants. Am. J. Hum. Genet..

[28] Jian X, Liu X (2017). In silico prediction of deleteriousness for nonsynonymous and splice-altering single nucleotide variants in the human genome. Methods Mol. Biol..

[29] Caliskan M (2019). Genetic and epigenetic fine mapping of complex trait associated loci in the human liver. Am. J. Hum. Genet.

[30] Li L (2017). Single-cell RNA-seq analysis maps development of human germline cells and gonadal niche interactions. Cell Stem Cell.

[31] Su C (2020). Mapping effector genes at lupus GWAS loci using promoter Capture-C in follicular helper T cells. Nat. Commun..

[32] Chesi A (2019). Genome-scale Capture C promoter interactions implicate effector genes at GWAS loci for bone mineral density. Nat. Commun..

[33] Kichaev G (2014). Integrating functional data to prioritize causal variants in statistical fine-mapping studies. PLoS Genet.

[34] Fachal L (2020). Fine-mapping of 150 breast cancer risk regions identifies 191 likely target genes. Nat. Genet.

[35] Mavaddat N (2019). Polygenic risk scores for prediction of breast cancer and breast cancer subtypes. Am. J. Hum. Genet..

[36] Stevens L, Mackensen J (1961). Genetic and environmental influences on teratocarcinogenesis in mice. J. Natl Cancer Inst..

[37] Heaney JD, Lam MY, Michelson MV, Nadeau JH (2008). Loss of the transmembrane but not the soluble kit ligand isoform increases testicular germ cell tumor susceptibility in mice. Cancer Res..

[38] Yamaji M (2008). Critical function of Prdm14 for the establishment of the germ cell lineage in mice. Nat. Genet..

[39] Sladitschek HL, Neveu PA (2019). A gene regulatory network controls the balance between mesendoderm and ectoderm at pluripotency exit. Mol. Syst. Biol..

[40] Mullen RD, Ontiveros AE, Moses MM, Behringer RR (2019). AMH and AMHR2 mutations: a spectrum of reproductive phenotypes across vertebrate species. Dev. Biol..

[41] Morinaga C (2007). The hotei mutation of medaka in the anti-Mullerian hormone receptor causes the dysregulation of germ cell and sexual development. Proc. Natl Acad. Sci. USA.

[42] Hornig NC, Holterhus PM (2020). Molecular basis of androgen insensitivity syndromes. Mol. Cell Endocrinol..

[43] Hagenaars SP (2017). Genetic prediction of male pattern baldness. PLoS Genet.

[44] Moirano G (2016). Baldness and testicular cancer: the EPSAM case-control study. Andrology.

[45] Rajpert-De Meyts E, Skakkebaek NE (1992). Immunohistochemical identification of androgen receptors in germ cell neoplasia. J. Endocrinol..

[46] Henderson BE, Ross RK, Pike MC, Casagrande JT (1982). Endogenous hormones as a major factor in human cancer. Cancer Res..

[47] Murphy MW (2015). An ancient protein-DNA interaction underlying metazoan sex determination. Nat. Struct. Mol. Biol..

[48] Krentz AD (2013). Interaction between DMRT1 function and genetic background modulates signaling and pluripotency to control tumor susceptibility in the fetal germ line. Dev. Biol..

[49] Krentz AD (2009). The DM domain protein DMRT1 is a dose-sensitive regulator of fetal germ cell proliferation and pluripotency. Proc. Natl Acad. Sci. USA.

[50] Nicholls PK (2019). Mammalian germ cells are determined after PGC colonization of the nascent gonad. Proc. Natl Acad. Sci. USA.

[51] Li H (2019). DAZL is a master translational regulator of murine spermatogenesis. Natl Sci. Rev..

[52] Luo X, O’Neill KL, Huang K (2020). The third model of Bax/Bak activation: a Bcl-2 family feud finally resolved?. F1000Res..

[53] Dewson G (2008). To trigger apoptosis, Bak exposes its BH3 domain and homodimerizes via BH3:groove interactions. Mol. Cell.

[54] Kim H (2009). Stepwise activation of BAX and BAK by tBID, BIM, and PUMA initiates mitochondrial apoptosis. Mol. Cell.

[55] Sarosiek KA (2013). BID preferentially activates BAK while BIM preferentially activates BAX, affecting chemotherapy response. Mol. Cell.

[56] Coultas L (2005). Concomitant loss of proapoptotic BH3-only Bcl-2 antagonists Bik and Bim arrests spermatogenesis. EMBO J..

[57] Stallock J, Molyneaux K, Schaible K, Knudson CM, Wylie C (2003). The pro-apoptotic gene Bax is required for the death of ectopic primordial germ cells during their migration in the mouse embryo. Development.

[58] Runyan C, Gu Y, Shoemaker A, Looijenga L, Wylie C (2008). The distribution and behavior of extragonadal primordial germ cells in Bax mutant mice suggest a novel origin for sacrococcygeal germ cell tumors. Int. J. Dev. Biol..

[59] Katz SG (2013). Brain and testicular tumors in mice with progenitor cells lacking BAX and BAK. Oncogene.

[60] Taylor-Weiner A (2016). Genomic evolution and chemoresistance in germ-cell tumours. Nature.

[61] Taylor AM (2018). Genomic and functional approaches to understanding cancer aneuploidy. Cancer Cell.

[62] Foley EA, Maldonado M, Kapoor TM (2011). Formation of stable attachments between kinetochores and microtubules depends on the B56-PP2A phosphatase. Nat. Cell Biol..

[63] Matson DR, Stukenberg PT (2014). CENP-I and Aurora B act as a molecular switch that ties RZZ/Mad1 recruitment to kinetochore attachment status. J. Cell Biol..

[64] Liu ST (2003). Human CENP-I specifies localization of CENP-F, MAD1 and MAD2 to kinetochores and is essential for mitosis. Nat. Cell Biol..

[65] Goyal U, Renvoisé B, Chang J, Blackstone C (2014). Spastin-interacting protein NA14/SSNA1 functions in cytokinesis and axon development. PLoS ONE.

[66] Chang L, Zhang Z, Yang J, McLaughlin SH, Barford D (2015). Atomic structure of the APC/C and its mechanism of protein ubiquitination. Nature.

[67] Lang S (2019). Functions and mechanisms of the human ribosome-translocon complex. Subcell. Biochem..

[68] Tang WW, Kobayashi T, Irie N, Dietmann S, Surani MA (2016). Specification and epigenetic programming of the human germ line. Nat. Rev. Genet..

[69] Patterson N, Price AL, Reich D (2006). Population structure and eigenanalysis. PLoS Genet..

[70] Price AL (2006). Principal components analysis corrects for stratification in genome-wide association studies. Nat. Genet..

[71] McCarthy S (2016). A reference panel of 64,976 haplotypes for genotype imputation. Nat. Genet..

[72] Loh PR (2016). Reference-based phasing using the Haplotype Reference Consortium panel. Nat. Genet.

[73] Das S (2016). Next-generation genotype imputation service and methods. Nat. Genet.

[74] Marchini J, Howie B (2010). Genotype imputation for genome-wide association studies. Nat. Rev. Genet..

[75] Willer CJ, Li Y, Abecasis GR (2010). METAL: fast and efficient meta-analysis of genomewide association scans. Bioinformatics.

[76] Yang J (2012). Conditional and joint multiple-SNP analysis of GWAS summary statistics identifies additional variants influencing complex traits. Nat. Genet..

[77] Hemminki K, Li X (2004). Familial risk in testicular cancer as a clue to a heritable and environmental etiology. Br. J. Cancer.

[78] Bray, F. et al. *Cancer Incidence in Five Continents, Vol. XI* (International Agency for Research on Cancer, 2017).

[79] Alexander TA, Machiela MJ (2020). LDpop: an interactive online tool to calculate and visualize geographic LD patterns. BMC Bioinforma..

[80] Machiela MJ, Chanock SJ (2018). LDassoc: an online tool for interactively exploring genome-wide association study results and prioritizing variants for functional investigation. Bioinformatics.

[81] Machiela MJ, Chanock SJ (2015). LDlink: a web-based application for exploring population-specific haplotype structure and linking correlated alleles of possible functional variants. Bioinformatics.

[82] Chang, X. & Wang, K. wANNOVAR: annotating genetic variants for personal genomes via the web. *J. Med. Genet*. **49**, 433–436 (2012). 10.1136/jmedgenet-2012-100918 PMC3556337 22717648

[83] GTEx Consortium. The GTEx Consortium atlas of genetic regulatory effects across human tissues. *Science* **369**, 1318–1330 (2020). 10.1126/science.aaz1776 PMC7737656 32913098

[84] Berisa T, Pickrell JK (2016). Approximately independent linkage disequilibrium blocks in human populations. Bioinformatics.

[85] Battle A, Brown CD, Engelhardt BE, Montgomery SB (2017). Genetic effects on gene expression across human tissues. Nature.

[86] Hughes JR (2014). Analysis of hundreds of cis-regulatory landscapes at high resolution in a single, high-throughput experiment. Nat. Genet..

[87] Cairns J (2016). CHiCAGO: robust detection of DNA looping interactions in Capture Hi-C data. Genome Biol..

[88] Watanabe K, Taskesen E, van Bochoven A, Posthuma D (2017). Functional mapping and annotation of genetic associations with FUMA. Nat. Commun..

[89] Jain A, Tuteja G (2019). TissueEnrich: tissue-specific gene enrichment analysis. Bioinformatics.

[90] Davis CA (2018). The Encyclopedia of DNA elements (ENCODE): data portal update. Nucleic Acids Res..

[91] Kichaev G (2017). Improved methods for multi-trait fine mapping of pleiotropic risk loci. Bioinformatics.

[92] Kichaev G, Pasaniuc B (2015). Leveraging functional-annotation data in trans-ethnic fine-mapping studies. Am. J. Hum. Genet..

[93] Coetzee SG, Coetzee GA, Hazelett DJ (2015). motifbreakR: an R/Bioconductor package for predicting variant effects at transcription factor binding sites. Bioinformatics.

