# Supplementary material for: Identification of 22 susceptibility loci associated with testicular germ cell tumors
Source: Nat Commun. 2021 Jul 23;12:4487. doi: 10.1038/s41467-021-24334-y (PMC8302763; doi:10.1038/s41467-021-24334-y)
Supplement: Supplementary file 1 — Supplementary Information [file 41467_2021_24334_MOESM1_ESM.pdf]

SUPPLEMENTARY INFORMATION

Identification of 22 novel loci associated with susceptibility to testicular germ cell tumors

John Pluta, Louise C. Pyle, Kevin T. Nead, Rona Wilf, Mingyao Li, Nandita Mitra, Benita Weathers, Kurt D'Andrea, Kristian Almstrup, Lynn Anson-Cartwright, Javier Benitez, Christopher D. Brown, Stephen Chanock, Chu Chen, Victoria K. Cortessis, Alberto Ferlin, Carlo Foresta, Marija Gamulin, Jourik A. Gietema, Chiara Grasso, Mark Greene, Tom Grotmol, Robert Hamilton, Trine B. Haugen, Russ Hauser, Michelle A. T. Hildebrandt, Matthew E. Johnson, , Robert Karlsson, Lambertus A. Kiemeney, Davor Lessel, Ragnhild A. Lothe, Jennifer T. Loud, Chey Loveday, Paloma Martin-Gimeno, Coby Meijer, Jérémie Nsengimana, David I. Quinn, Thorunn Rafnar, Shweta Ramdas, Lorenzo Richiardi, Rolf I. Skotheim, Kari Stefansson, Clare Turnbull, David J. Vaughn, Fredrik Wiklund, Xifeng Wu, Daphne Yang, Tongzhang Zheng, Andrew D. Wells, Struan F. A. Grant, Ewa Rajpert-De Meyts, Stephen M. Schwartz, D. Timothy Bishop, Katherine A. McGlynn, Peter A. Kanetsky*, Katherine L. Nathanson* & the Testicular Cancer Consortium

Table of contents

Supplementary Table 1. TGCT GWAS and corresponding subjects included in the current study 4

Supplementary Table 2. TECAC sites and corresponding subjects with *de novo* genome-wide genotyping that passed quality control measures and were used in the current study 5

Supplementary Table 3. TECAC sites and corresponding subjects with targeted genotyping used in the current study 6

Supplementary Table 4. Targeted genotyping panel and corresponding results from validation of imputed genotypes 7

Supplementary Table 5. Pairwise linkage disequilibrium (r2) among independent SNPs in previously identified regions. 8

Supplementary Table 6. Number of subjects included in TGCT stratified analyses 9

Supplementary Table 7. Comparison of newly identified SNP frequencies associated with TGCT across genetic ancestry 10

Supplementary Table 8. Evaluation of potential target genes contained in gene regions corresponding to top signals on the X chromosome 11

Supplementary Table 15. Data sets used to annotate PAINTOR 12

Supplementary Fig. 1. Regional association plots of novel susceptibility loci 13-24

Supplementary Fig. 2. Conditional analysis plots showing independence of novel susceptibility loci in previously identified regions 25-27

Supplementary Fig. 3. QQ-plot for TGCT meta-analysis 28

Supplementary Fig. 4. Diagram of five independent nearby regions on chromosome 19 associated with TGCT susceptibility 29

Supplementary Fig. 5. Representative images of high, medium, and low expression

levels in fetal gonads of potential target genes 30

Supplementary Fig. 6. Heatmap of tissue-specific gene expression for target genes moderately or highly likely to be associated with TGCT 31

Supplementary Fig. 7. Schematic example of functional annotation of novel loci............................... 32

Supplementary Fig. 8. Principal components analysis of TECAC samples with *de novo* genome-wide genotyping 33

Supplementary Methods 34-42

References 43-45

Supplementary Table 1. TGCT GWAS and corresponding subjects included in the current study

Site	Case	Control	Total	Data	Imputation Backbone	Reference
Denmark	183	363	546	Summary Statistics	1000 Genomes	Dalgaard et al.[1]
Karolinska Institutet/Cancer Registry of Norway/OsloMet – Oslo Metropolitan University	1,326	6,687	8,013	Summary Statistics	1000 Genomes	Kristiansen et al.[2]
National Cancer Institute	581	1,056	1,637	Genotype	1000 Genomes	Schumacher et al.[3]
University of Pennsylvania	481	919	1,400	Genotype	1000 Genomes	Kanetsky et al.[4]
Institute of Cancer Research	986	4,945	5,931	Genotype	1000 Genomes	Litchfield et al.[5]
TECAC (**Supp Table 2**)	5,602	5,006	10,608	Genotype	HRC*	Current Study
deCODE Genetics	300	151,991	152,291	Summary Statistics	Whole Genome Sequencing	Gudbjartsson et al.[6]
UK Biobank	697	8,716	9,413	Summary Statistics	HRC*	Bycroft et al.[7]
**Total**	**10,156**	**179,683**	**189,839**			

* Haplotype Reference Consortium

Supplementary Table 2. TECAC sites and corresponding subjects with *de novo* genome-wide genotyping that passed quality control measures and were used in the current study

Site	Location	Men with TGCT	Men without TGCT	Total
Fred Hutchinson Cancer Research Center	Seattle, WA	472	986	1,458
Karolinska Institutet/Cancer Registry of Norway/OsloMet – Oslo Metropolitan University*	Stockholm, Sweden	868	516	1,384
MD Anderson Cancer Center	Houston, TX	271	37	308
Princess Margaret Cancer Center	Toronto, Ontario	380	0	380
Radboud University of Njimegen	Nijmegen, Netherlands	299	1,212	1,511
University Medical Center Hamburg-Eppendorf	Hamburg, Germany	427	426	853
University of Groningen	Groningen, Netherlands	487	0	487
University of Leeds	Leeds, UK	381	239	620
Oslo University Hospital	Oslo, Norway	660	0	660
University of Padova	Padova, Italy	323	336	659
University of Pennsylvania	Philadelphia, PA	254	618	872
University of Southern California	Los Angeles, CA	406	0	406
University of Turin	Torino, Italy	180	275	455
Yale University	New Haven, CT	194	361	555
**Total**		**5,602**	**5,006**	**10,608**

*Newly genotyped controls from Karolinska Institutet/Cancer Registry of Norway are first or second degree relatives of cases that were not included in this analysis.

Supplementary Table 3. TECAC sites and corresponding subjects with targeted genotyping used in the current study

Site	Men with TGCT	Men without TGCT	Total
Fred Hutchinson Cancer Research Center	3	9	12
Karolinska Institutet/Cancer Registry of Norway/OsloMet – Oslo Metropolitan University	91	222	313
MD Anderson Cancer Center	0	0	0
Princess Margaret Cancer Center	31	0	31
Radboud University of Njimegen	0	4	4
Spanish National Cancer Research Center	481	376	857
University Medical Center Hamburg-Eppendorf	1	0	1
University of Groningen	6	4	10
University of Leeds	42	65	107
Oslo University Hospital	10	343	353
University of Padova	77	38	115
University of Pennsylvania	277	289	566
University of Southern California	2	12	14
University of Turin	17	33	50
Yale University	1	3	4
**Total**	**1,039**	**1,398**	**2,437**

Supplementary Table 4. Targeted genotyping panel and corresponding results from validation of imputed genotypes

rsID	Chr:location (hg19)	Correlation*	n
rs340773	2:16464895	0.98	464
rs6761764	2:65684395	0.92	497
rs6708784	2:111927379	-	-
rs4353792	3:17078640	0.99	497
rs2117754	3:149363508	-	-
rs4333176	4:10475280	-	-
rs7734992	5:1280128	-	-
rs13355456	5:162350166	0.94	497
rs9469079	6:32032421	0.97	497
rs6910233	6:33534726	-	-
rs55694649	6:165769357	0.94	497
rs7789337	7:138788795	0.99	497
rs4735315	8:95661772	0.98	496
rs9987332	8:120933963	0.96	497
rs10976519	9:779507	0.95	497
rs13289835	9:33069181	0.97	497
rs10818964	9:127190340	-	-
rs4144630	9:127439200	0.97	497
rs28393706	9:140073294	0.98	497
rs7912968	10:7534248	0.96	497
rs10786745	10:104956827	0.99	496
rs7927974	11:30351223	-	-
rs10751136	11:85910309	0.97	496
rs2887532	12:1051495	-	-
rs4931592	12:32141419	0.95	497
rs12830125	12:51301431	0.98	496
rs35969688	12:53793209	0.95	497
rs10160879	12:88963746	0.94	497
rs4766561	12:111673244	0.94	494
rs114583024	14:90727628	0.95	497
rs4786384	16:3136919	-	-
rs55779573	17:76691564	0.92	497
rs2741167	18:703785	0.92	497
rs2156650	18:45733788	-	-
rs10775491	18:46688467	0.98	497
rs34541064	19:1850165	0.96	495
rs12461745	19:22262667	0.97	497
rs58333554	19:36910208	0.96	497
rs6068588	20:52197366	-	-
rs6090457	20:62200576	0.96	497
rs16988720	22:30909767	0.97	497
rs67465697	22:40531645	0.94	497
rs72620486	X:24384181	0.83	483
rs2360670	X:100432681	0.98	483
rs4528012	X:106916533	-	-
rs9698786	X:131532809	0.97	483
**Average**		0.96	495

The 46 SNPs on the targeted genotyping panel are listed. * (-) indicates a genotyped SNP. The correlation coefficient between observed genotype on the targeted panel and imputed genotype inferred from genome-wide genotyping for 36 susceptibility markers is shown.

Supplementary Table 5. Pairwise linkage disequilibrium (r^2) among independent SNPs in previously identified regions**.** Novel hits are highlighted.

TERT	rs2735940	rs36115365	rs7734992
rs2735940	1	0.26	0.14
rs36115365	-	1	0.11
rs7734992	-	-	1

BAK1	rs141079110
rs210137	0.04

DMRT1	rs755383	rs7039737	rs55873183	rs10976519
rs755383	1	0.38	0.04	0.003
rs7039737	-	1	0.02	0.0005
rs55873183	-	-	1	0.004
rs10976519	-	-	-	1

TKTL1	rs17336718
rs4898474	0.19

Supplementary Table 6. Number of subjects included in TGCT stratified analyses.*

Site	Histological subtype			Family History		Cryptorchidism	
	**Seminoma**	**Non-seminoma**	**Mixed germ cell tumor**	**Yes**	**No**	**Yes**	**No**
Denmark	88	55	55	-	-	-	-
Karolinska Institutet/Cancer Registry of Norway/OsloMet – Oslo Metropolitan University	766	549	549	-	-	-	-
National Cancer Institute	243	309	334	76	505	131	450
University of Pennsylvania	171	238	299	49	432	39	442
Institute of Cancer Research	410	319	410	136	850	56	930
TECAC (**Supp Table 2**)	2,456	1,897	2,760	95	5507	295	5307
UK Biobank	395	223	223	-	-	-	-
**Total**	4,529	3,590	4,630	356	7294	521	7129

Associations were tested using a two-sided Wald test on the logistic regression coefficient with an alpha level of 5×10^{-8} to account for multiple comparisons.
*No statistically significant associations ($P \leq 5 \times 10^{-8}$) were noted for any subgroup.

Supplementary Table 7. Comparison of newly identified SNP frequencies* associated with TGCT across genetic ancestry.

rsID	Chr	Location (hg19)	A1/A2*	A1 frequency	European		African		$P_{\text{(African v. European)}}$	East Asian		$P_{\text{(East Asian v. European)}}$
					A1	A2	A1	A2		A1	A2	
rs351418	1	212449403	T/C	0.38	0.43	0.57	0.09	0.91	$\ll 1.0 \times 10^{-10}$	0.65	0.35	$\ll 1.0 \times 10^{-10}$
rs6708784	2	111927379	G/A	0.50	0.49	0.51	0.36	0.64	$\ll 1.0 \times 10^{-10}$	0.43	0.57	0.01
rs7734992	5	1280128	T/C	0.60	0.57	0.43	0.33	0.67	$\ll 1.0 \times 10^{-10}$	0.61	0.39	0.15
rs9469079	6	32032421	T/C	0.13	0.12	0.88	0.22	0.78	$\ll 1.0 \times 10^{-10}$	0.13	0.87	0.49
rs141079110	6	33533625	A/G	0.75	0.76	0.24	0.77	0.23	0.0006	0.96	0.04	$\ll 1.0 \times 10^{-10}$
rs9987332	8	120933963	A/G	0.44	0.46	0.54	0.69	0.31	$\ll 1.0 \times 10^{-10}$	0.95	0.05	$\ll 1.0 \times 10^{-10}$
rs10976519	9	779507	G/T	0.42	0.42	0.58	0.74	0.26	$\ll 1.0 \times 10^{-10}$	0.52	0.48	6.29×10^{-5}
rs10818964*	9	127190340	G/A	0.67	0.67	0.33	0.26	0.74	$\ll 1.0 \times 10^{-10}$	0.36	0.64	$\ll 1.0 \times 10^{-10}$
rs28393706	9	140073294	T/C	0.75	0.75	0.25	0.63	0.37	$\ll 1.0 \times 10^{-10}$	0.94	0.06	$\ll 1.0 \times 10^{-10}$
rs7912968	10	7534248	C/G	0.38	0.34	0.66	0.11	0.89	$\ll 1.0 \times 10^{-10}$	0.58	0.42	$\ll 1.0 \times 10^{-10}$
rs7927974	11	30351223	G/A	0.29	0.31	0.69	0.44	0.56	$\ll 1.0 \times 10^{-10}$	0.58	0.42	$\ll 1.0 \times 10^{-10}$
rs2887532	11	1051495	C/T	0.82	0.81	0.19	0.97	0.03	$\ll 1.0 \times 10^{-10}$	0.93	0.07	$\ll 1.0 \times 10^{-10}$
rs12830125	12	51301431	C/G	0.34	0.33	0.67	0.13	0.87	$\ll 1.0 \times 10^{-10}$	0.24	0.76	4.37×10^{-9}
rs35969688	12	53793209	A/G	0.18	0.17	0.83	0.20	0.80	$\ll 1.0 \times 10^{-10}$	0.20	0.80	0.05
rs55779573	17	76691564	C/T	0.53	0.57	0.43	0.12	0.88	$\ll 1.0 \times 10^{-10}$	0.42	0.58	3.84×10^{-9}
rs2847334	18	692095	G/A	0.57	0.56	0.44	0.79	0.21	$\ll 1.0 \times 10^{-10}$	0.69	0.31	1.36×10^{-6}
rs8104804	19	28356614	C/T	0.19	0.20	0.80	0.32	0.68	$\ll 1.0 \times 10^{-10}$	0.25	0.75	0.00
rs6068588	20	52197366	A/C	0.12	0.14	0.86	0.21	0.79	$\ll 1.0 \times 10^{-10}$	0.07	0.93	7.27×10^{-11}
rs72620486	X	24384181	T/C	0.15	0.15	0.85	0.06	0.94	$\ll 1.0 \times 10^{-10}$	0.12	0.88	0.26
rs2335864	X	66489986	G/A	0.20	0.18	0.82	0.87	0.13	$\ll 1.0 \times 10^{-10}$	0.00	1.00	0.00
rs2360670	X	100432681	A/T	0.54	0.56	0.44	0.39	0.61	$\ll 1.0 \times 10^{-10}$	0.67	0.33	1.18×10^{-6}
rs4898474	X	153535143	C/T	0.31	0.28	0.72	0.66	0.34	$\ll 1.0 \times 10^{-10}$	0.44	0.57	4.81×10^{-11}

* All SNP frequencies obtain from gnomAD with the exception of rs10818964 obtained from 1000G. Chr, chromosome; A1, risk allele; *P* , *P* value for two-tailed Fisher's Exact test.

Supplementary Table 8. Evaluation of potential target genes contained in gene regions corresponding to top signals on the X chromosome.

			Top signal			Genes in region			Location of top signal		eQTL			Expression in fetal testis			Promoter Capture-C contact				
Fig. 1 label	Chr	Cytoband	rsID	Position	Number of CRVs	Gene*	Number	Score	Location	Score	Count in non-testis tissue	In testis tissue	Score	Count	Category	Score	Number of cell lines	Score	Total Score	Target gene likelihood	Maximum possible target gene likelihood**
s	X	Xp22.11	rs72620486	24384181	29	SUPT20HL1	2	1	5' UTR	1	no data	no data	N/A	13	Low	0	0	0	2	Moderate	High
						PDK3	2	1	Proximal	0	no data	no data	N/A	3239	High	1	0	0	2	Moderate	High
t	X	Xq12	rs2335864	66489986	177	AR	0	0	Proximal	0	no data	no data	N/A	no data	no data	N/A	0	0	0	Low	High
u	X	Xq22.1	rs2360670	100432681	27	CENPI	2	1	Distal	0	no data	no data	N/A	171	Low	0	0	0	1	Low	High
						DRP2	2	1	Proximal	0	no data	no data	N/A	62	Low	0	0	0	1	Low	High
v	X	Xq28	rs4898474	153535143	37	TKTL1	2	1	Intron	1	no data	no data	N/A	1803	Moderate	0.5	0	0	3	High	High
						TEX28	2	1	Proximal	0	no data	no data	N/A	0	Low	0	0	0	1	Low	High
56	X	Xq28	rs17336718	153536119	1	TKTL1	1	2	Intron	1	no data	no data	N/A	1803	Moderate	0.5	0	0	4	High	High

* For top signals with no genes delimited by the CRV block, the closest gene(s) outside the block was evaluated. For top signals having at least one gene delimited by the CRV block, the gene located closest to the top signal is indicated in red.

** Target gene likelihood after adding the maximum score possible for evaluation of eQTL data (2, all genes) and expression in fetal testis (1, *AR* only).

Supplementary Table 9 Data sets* used to annotate PAINTOR.

Annotation	Cell/Tissue	Type	Category
ENCSR278FHC	Testis Embryo	DNAse-seq	Open Chromatin Marks
ENCSR611DJQ	Testis Adult	H3K4me3	Histone Marks
ENCSR000EXB	NT2-D1	H3K36me3	Histone Marks
ENCSR954IGQ	Testis Adult	H3K27ac	Histone Marks
ENCSR886NTH	Testis Adult	5-group	Open Chromatin Mark
ENCSR494TNM	Testis Adult	CTCF	Histone Marks
ENCSR841EQJ	Testis Adult	RAMPAGE	Transcription Start Sites
ENCSR898RGU	NT2-D1	H3K4me3	Open Chromatin Marks
ENCSR303XKE	Testis Fetal	DNAse I hypersensitivity	Open Chromatin Marks
ENCSR000EXG	NT2-D1	YY1	Transcription Factor Binding Sites
ENCSR418RNI	NT2-D1	5-group	Open Chromatin Marks
ENCSR000EXC	NT2-D1	H3K9ac	Histone Marks
ENCSR619EZG	Testis Adult	H3K4me3	Histone Marks
ENCSR091MNT	Testis Adult	H3K27me3	Histone Marks
ENCSR000EXE	NT2-D1	H3K27me3	Histone Marks
ENCSR376JOC	Testis Adult	H3K9me3	Histone Marks
ENCSR000EXA	NT2-D1	H3K4me1	Histone Marks
ENCSR136ZQZ	Testis Adult	H3K27ac	Histone Marks
ENCSR956VQB	Testis Adult	H3K4me1	Histone Marks
ENCSR000EWY	NT2-D1	ZNF274	Transcription Factor Binding Sites
ENCSR866SRG	Testis Adult	RAMPAGE	Transcription Start Sites
ENCFF841TKB	Testis Embryo	DNAse-seq	Open Chromatin Marks
ENCSR729DRB	Testis Embryo	DNAse-seq	Open Chromatin Marks
ENCSR981CID	Testis Adult	CTCF	Transcription Factor Binding Sites
ENCSR803FAP	Testis Adult	POLR2A	Transcription Factor Binding Sites
ENCSR000EWZ	NT2-D1	H3K9me3	Histone Marks
ENCSR000EPS	NT2-D1	DNAse-seq	Open Chromatin Marks
ENCSR080YRO	NT2-D1	RRBS	Methylation
ENCSR942OLI	Testis Adult	DNAme	Methylation
ENCSR962XHD	NT2-D1	DNAme	Methylation
ENCSR000DED	Testis Adult	RRBS	Methylation
ENCSR304AIL	Testis Adult	DNAme	Methylation
ENCSR806NNG	Testis Adult	Cpg Methylation	Methylation
ENCFF507JBR	Testis Adult	WGBS	Methylation
ENCFF038JFQ	Testis Adult	WGBS	Methylation
ENCFF715DMX	Testis Adult	WGBS	Methylation
NCCIT	NCCIT	ATACseq	Open Chromatin Marks
NT2-D1	NT2-D1	ATACseq	Open Chromatin Marks
TCAM2	TCAM2	ATACseq	Open Chromatin Marks
2102EP	2102EP	ATACseq	Open Chromatin Marks

* All datasets are publicly available from ENCODE except for ATACseq completed on NCCIT, NT2-D1, TCAM2, and 210EP cell lines, which were generated by TECAC members.

Supplementary Fig. 1 | Regional association plots of novel susceptibility loci. a-v, Regional association plots of genotypes imputed using the Haplotype Reference Consortium Panel were created using LocusZoom. Plots show a 500kb region (unless otherwise specified below) centered around the top signal identified in our meta-analysis, indicated by a purple circle (●), and displays -log$_{10}$(*P)*, LD structure, recombination, and gene regions. **a**, rs351418 (1:212449403). **b**, rs6708784 (2:111927379). **c**, rs7734992 (5:1280128). **d**, rs9469079 (6:32032421). **e**, rs141079110 (6:33533625). **f**, rs9987332 (8:120933963). **g**, rs10976519 (9:779507). **h**, rs10818964 (9:127190340). **i**, rs28393706 (9:140073294). **j**, rs7912968 (10:7534248). **k**, rs7927974 (11:30351223). **l**, rs2887532 (12:1051495). **m**, rs12830125 (12:51301431). **n**, 900kb region around rs35969688 (12:53793209). **o**, rs55779573 (17:76691564). **p**, rs2847334 (18:692095). **q**. 1000kb region around rs8104804 (19:28356614). **r**, rs6068588 (20:52197366). **s**, rs2360670 (X:100432681). **t**, rs4898474 (X:153535143). **u**, rs72620486 (X:24384181). **v**, 3000kb region around rs2335864 (X:66489986).

a

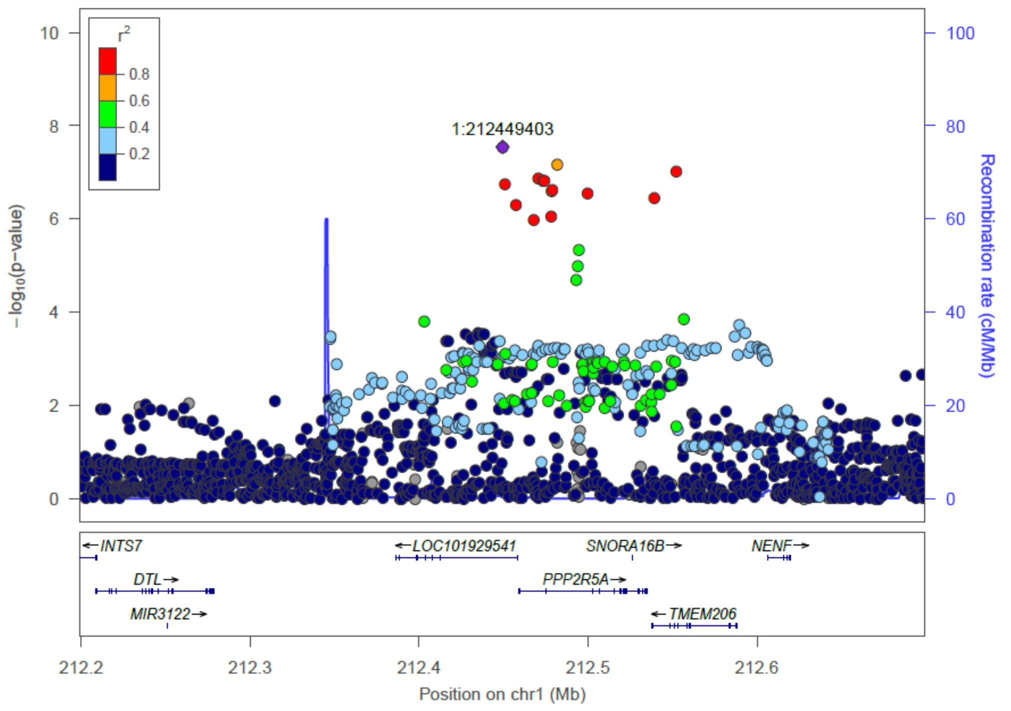

b

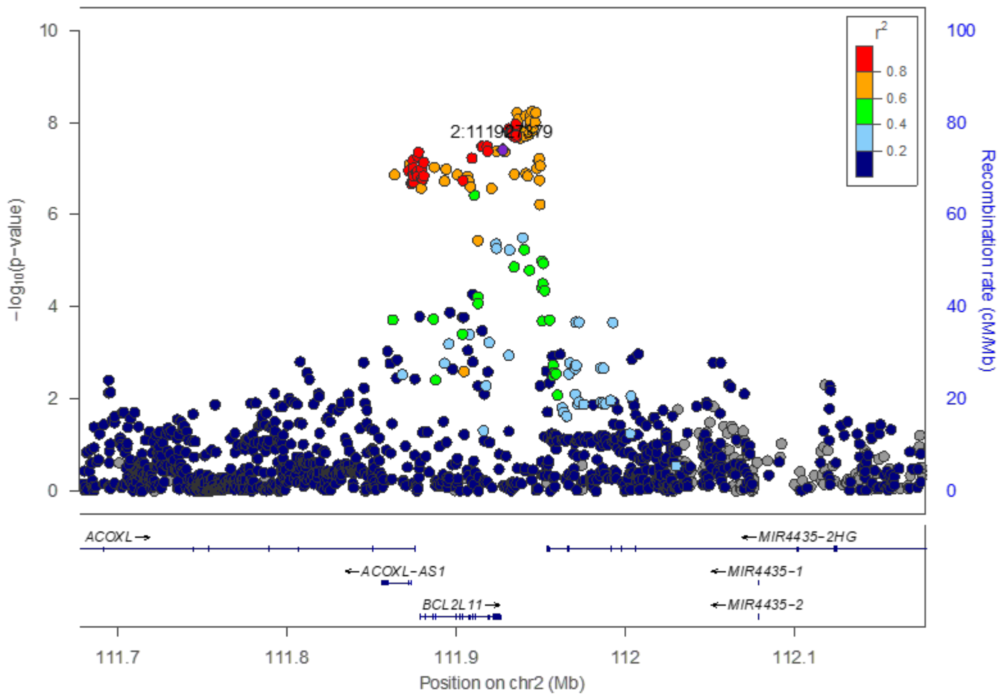

r2
0.8
0.6
0.4
0.2
10
8
6
4
2
0
−log10(p-value)
100
80
60
40
20
0
Recombination rate (cM/Mb)
ACOXL→
←MIR4435-2HG
←ACOXL-AS1
←MIR4435-1
BCL2L11→
←MIR4435-2
111.7
111.8
111.9
112
112.1
Position on chr2 (Mb)

c

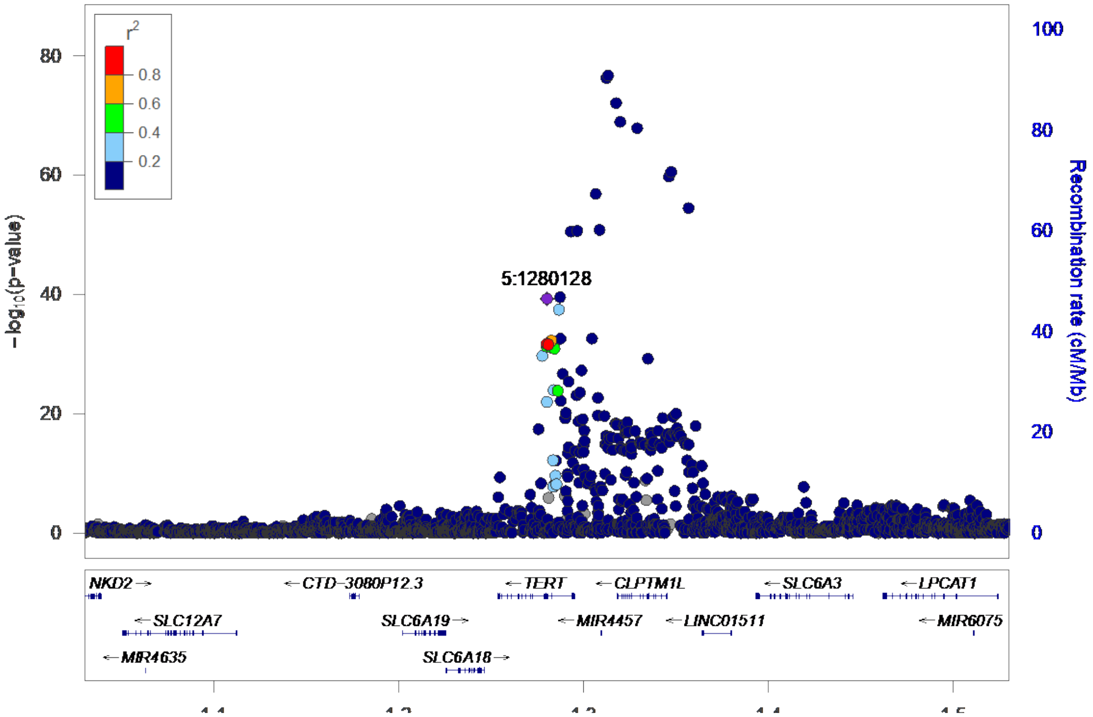

r2
0.8
0.6
0.4
0.2
80
60
40
20
0
−log10(p-value)
100
80
60
40
20
0
Recombination rate (cM/Mb)
5:1280128
NKD2→
←CTD-3080P12.3
←TERT
←CLPTM1L
←SLC6A3
←LPCAT1
←SLC12A7
SLC6A19→
←MIR4457
←LINC01511
←MIR6075
←MIR4635
SLC6A18→

d

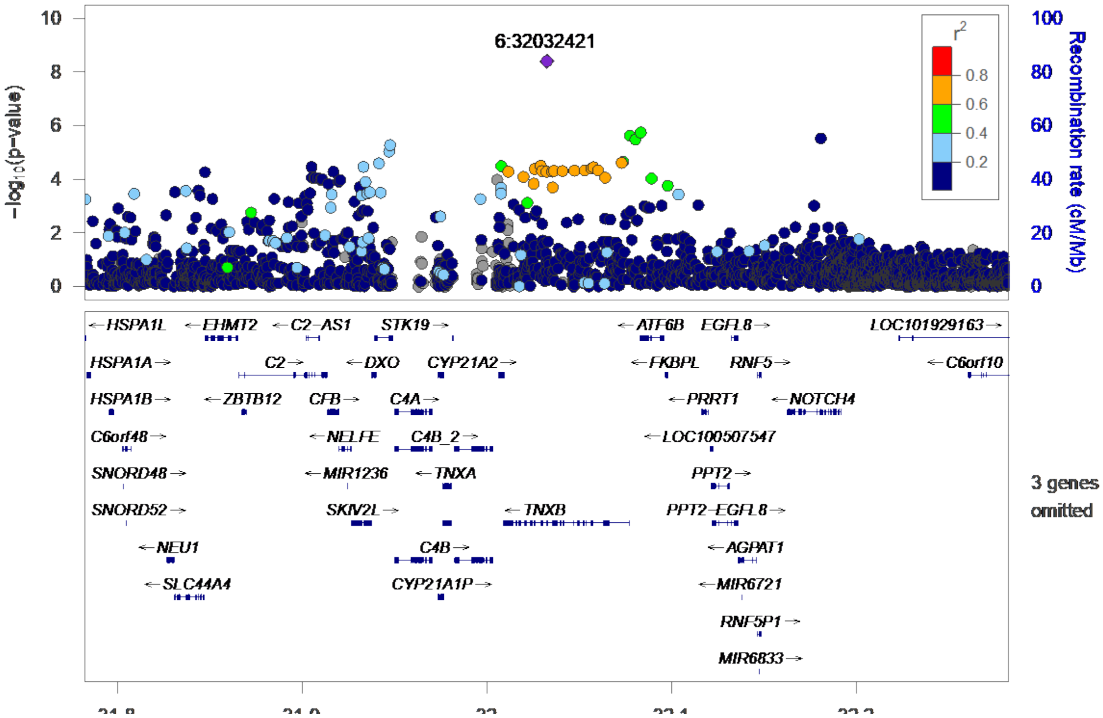
6:32032421
r2
0.8
0.6
0.4
0.2
−log10(p−value)
Recombination rate (cM/Mb)
HSPA1L
EHMT2
C2−AS1
STK19
ATF6B
EGFL8
LOC101929163
HSPA1A
C2
DXO
CYP21A2
FKBPL
RNF5
C6orf10
HSPA1B
ZBTB12
CFB
C4A
PRRT1
NOTCH4
C6orf48
NELFE
C4B_2
LOC100507547
SNORD48
MIR1236
TNXA
PPT2
SNORD52
SKIV2L
TNXB
PPT2−EGFL8
NEU1
C4B
AGPAT1
SLC44A4
CYP21A1P
MIR6721
RNF5P1
MIR6833
3 genes omitted

e

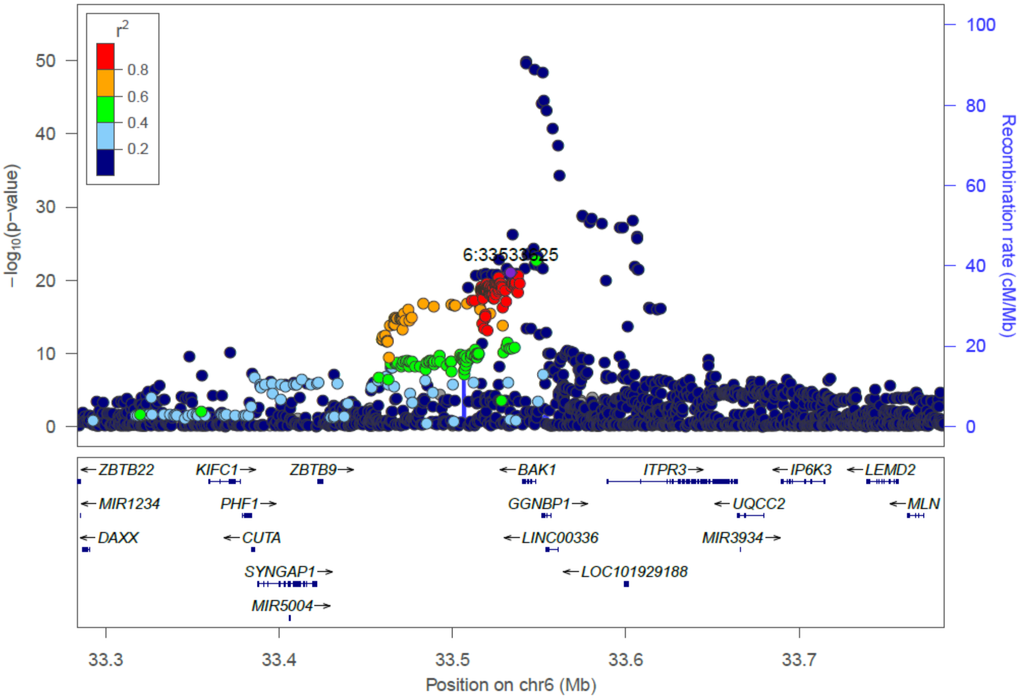
r2
0.8
0.6
0.4
0.2
6:33533625
−log10(p−value)
Recombination rate (cM/Mb)
ZBTB22
KIFC1
ZBTB9
BAK1
ITPR3
IP6K3
LEMD2
MIR1234
PHF1
GGNBP1
UQCC2
MLN
DAXX
CUTA
LINC00336
MIR3934
SYNGAP1
LOC101929188
MIR5004
33.3
33.4
33.5
33.6
33.7
Position on chr6 (Mb)

f

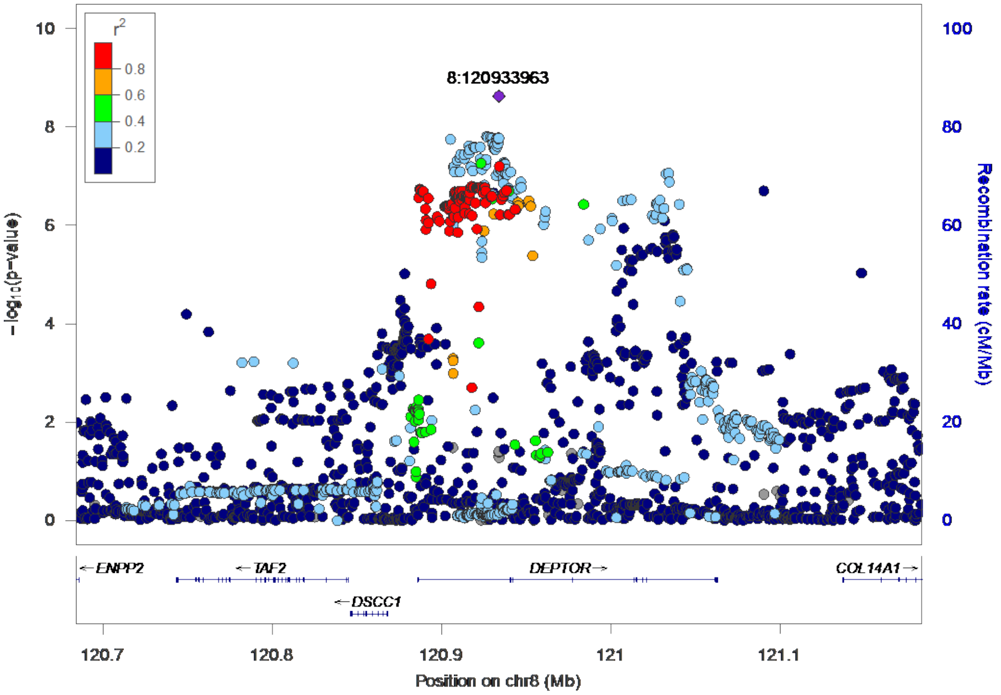

r2
0.8
0.6
0.4
0.2
8:120933963
−log10(p−value)
Recombination rate (cM/Mb)
ENPP2
TAF2
DSCC1
DEPTOR
COL14A1
120.7
120.8
120.9
121
121.1
Position on chr8 (Mb)

g

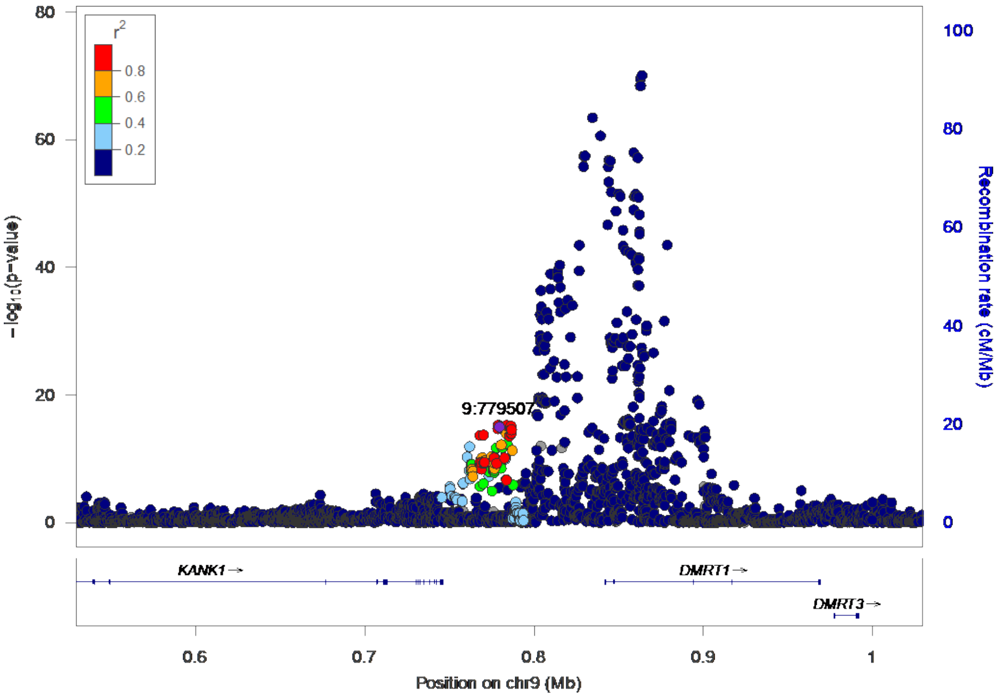

r2
0.8
0.6
0.4
0.2
9:779507
−log10(p−value)
Recombination rate (cM/Mb)
KANK1
DMRT1
DMRT3
0.6
0.7
0.8
0.9
1
Position on chr9 (Mb)

h

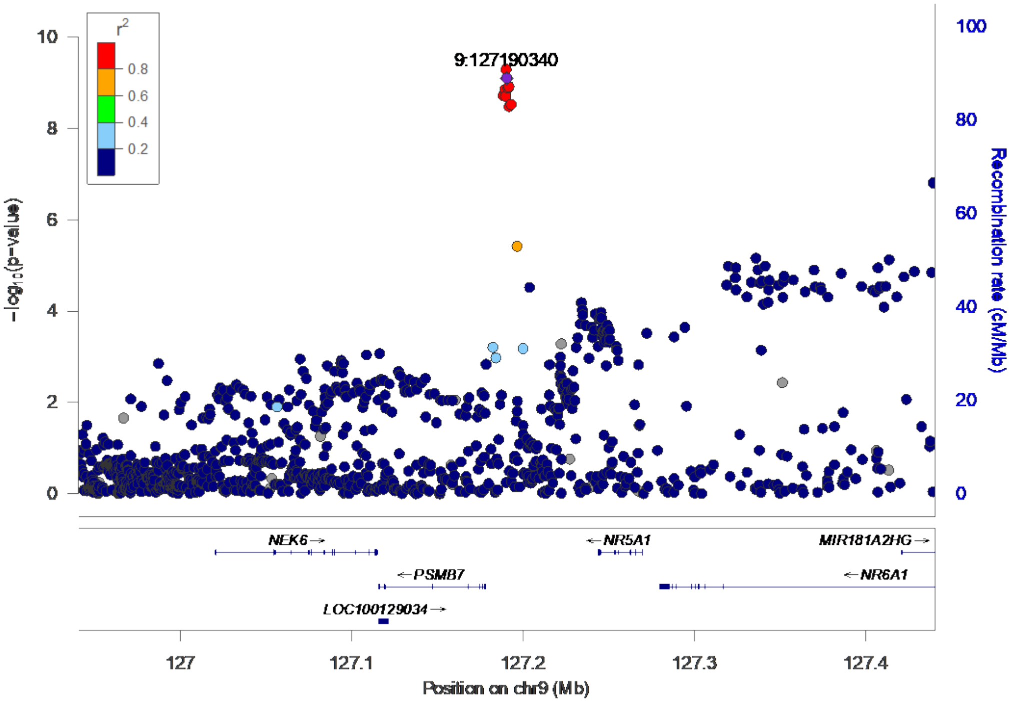
9:127190340
r2
0.8
0.6
0.4
0.2
−log10(p−value)
Recombination rate (cM/Mb)
NEK6→
←NR5A1
MIR181A2HG→
←PSMB7
←NR6A1
LOC100129034→
127
127.1
127.2
127.3
127.4
Position on chr9 (Mb)

i

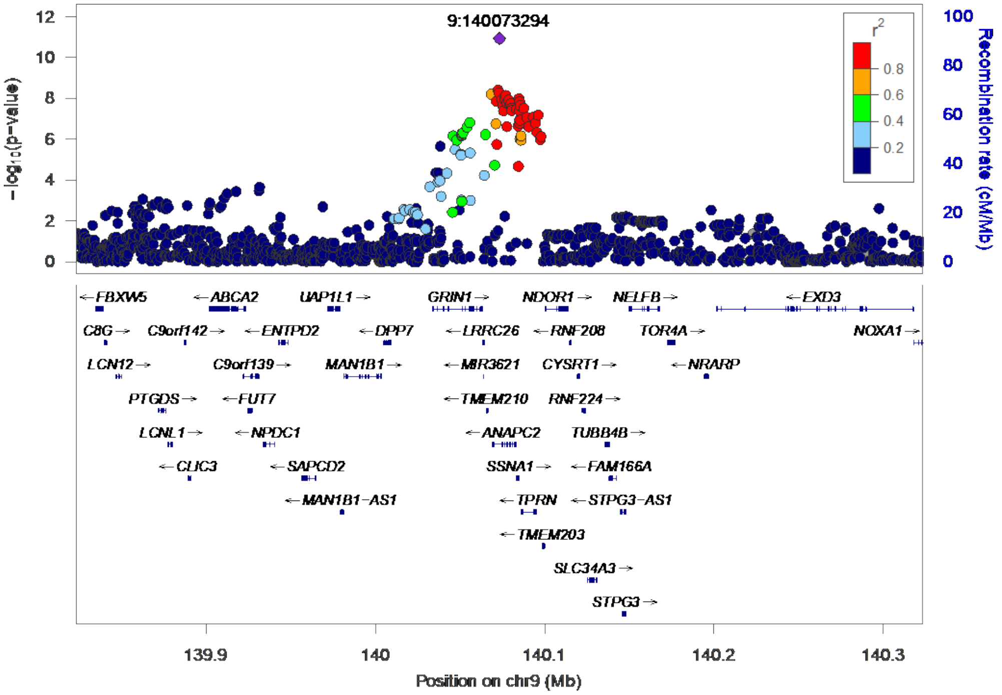
9:140073294
r2
0.8
0.6
0.4
0.2
−log10(p−value)
Recombination rate (cM/Mb)
←FBXW5
←ABCA2
UAP1L1→
GRIN1→
NDOR1→
NELFB→
←EXD3
C8G→
C9orf142→
←ENTPD2
←DPP7
←LRRC26
←RNF208
TOR4A→
NOXA1→
LCN12→
C9orf139→
MAN1B1→
←MIR3621
CYSRT1→
←NRARP
PTGDS→
←FUT7
←TMEM210
RNF224→
LCNL1→
←NPDC1
←ANAPC2
TUBB4B→
←CLIC3
←SAPCD2
SSNA1→
←FAM166A
←MAN1B1−AS1
←TPRN
←STPG3−AS1
←TMEM203
SLC34A3→
STPG3→
139.9
140
140.1
140.2
140.3
Position on chr9 (Mb)

j

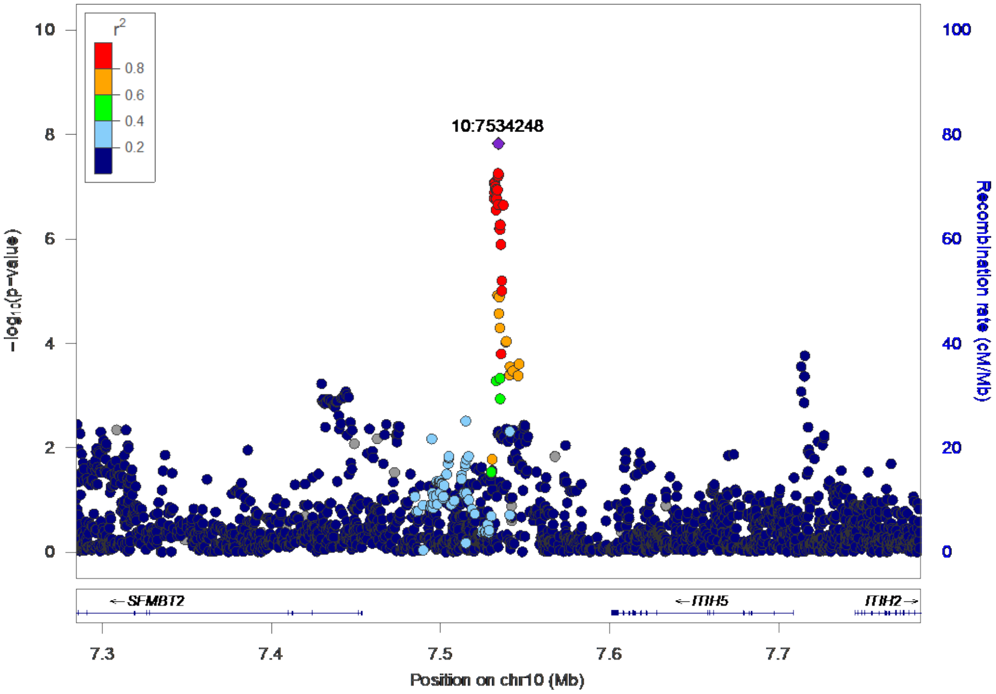

10:7534248
r²
0.8
0.6
0.4
0.2
−log₁₀(p−value)
Recombination rate (cM/Mb)
SFMBT2
ITIH5
ITIH2
Position on chr10 (Mb)

k

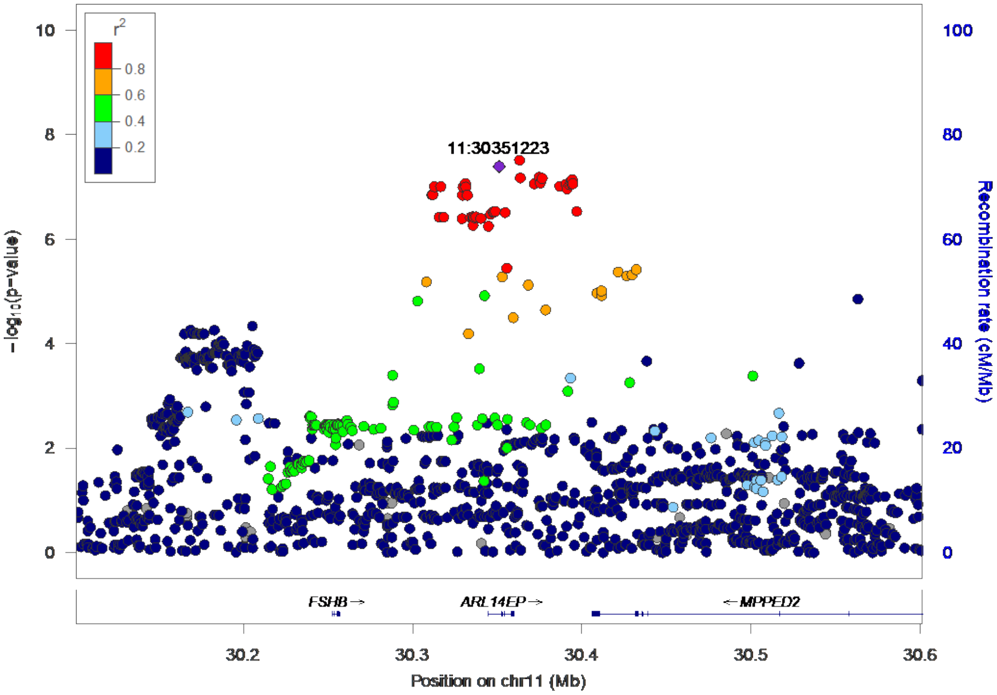

11:30351223
r²
0.8
0.6
0.4
0.2
−log₁₀(p−value)
Recombination rate (cM/Mb)
FSHB
ARL14EP
MPPED2
Position on chr11 (Mb)

l

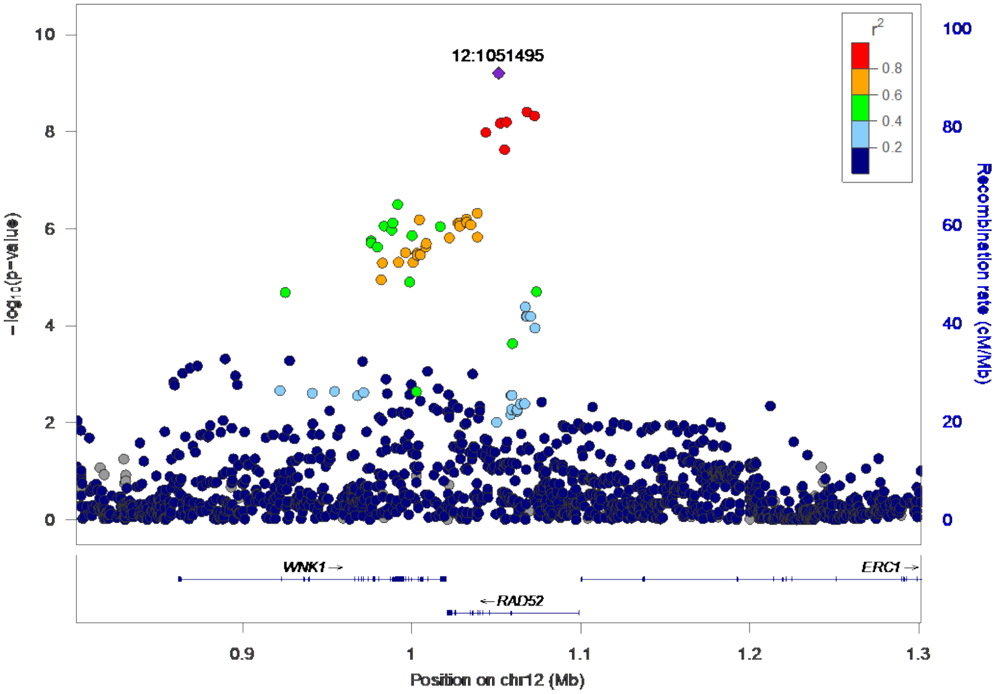
12:1051495
r2
0.8
0.6
0.4
0.2
−log10(p−value)
Recombination rate (cM/Mb)
WNK1→
←RAD52
ERC1→
0.9
1
1.1
1.2
1.3
Position on chr12 (Mb)

m

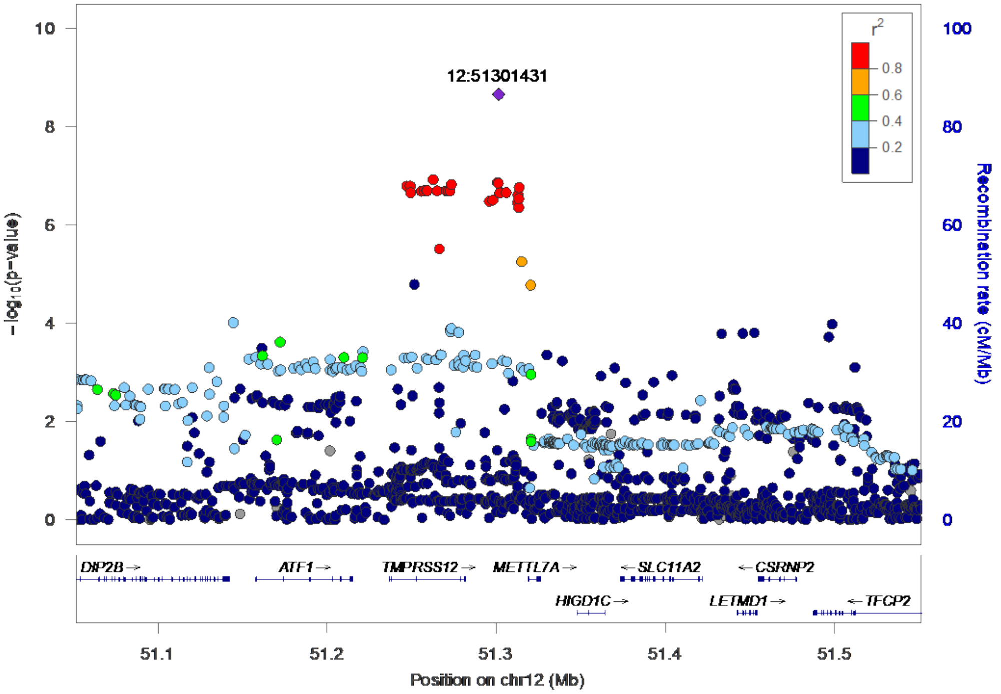
12:51301431
r2
0.8
0.6
0.4
0.2
−log10(p−value)
Recombination rate (cM/Mb)
DIP2B→
ATF1→
TMPRSS12→
METTL7A→
←SLC11A2
←CSRNP2
HIGD1C→
LETMD1→
←TFCP2
51.1
51.2
51.3
51.4
51.5
Position on chr12 (Mb)

n

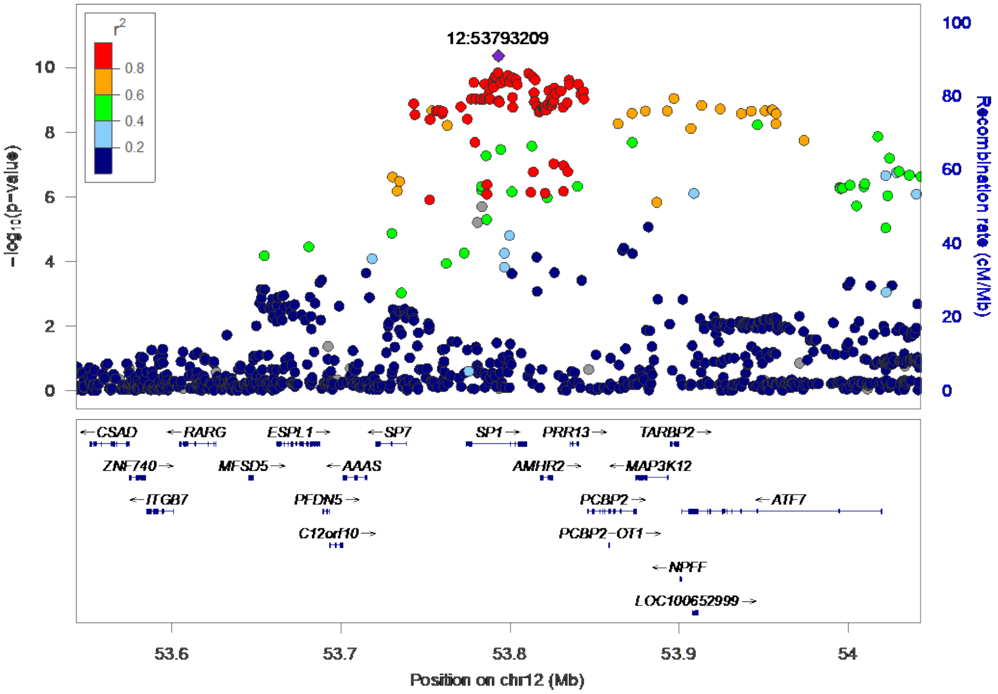
12:53793209
r2
0.8
0.6
0.4
0.2
−log10(p−value)
Recombination rate (cM/Mb)
CSAD
RARG
ESPL1
SP7
SP1
PRR13
TARBP2
ZNF740
MFSD5
AAAS
AMHR2
MAP3K12
ITGB7
PFDN5
PCBP2
ATF7
C12orf10
PCBP2−OT1
NPFF
LOC100652999
53.6
53.7
53.8
53.9
54
Position on chr12 (Mb)

o

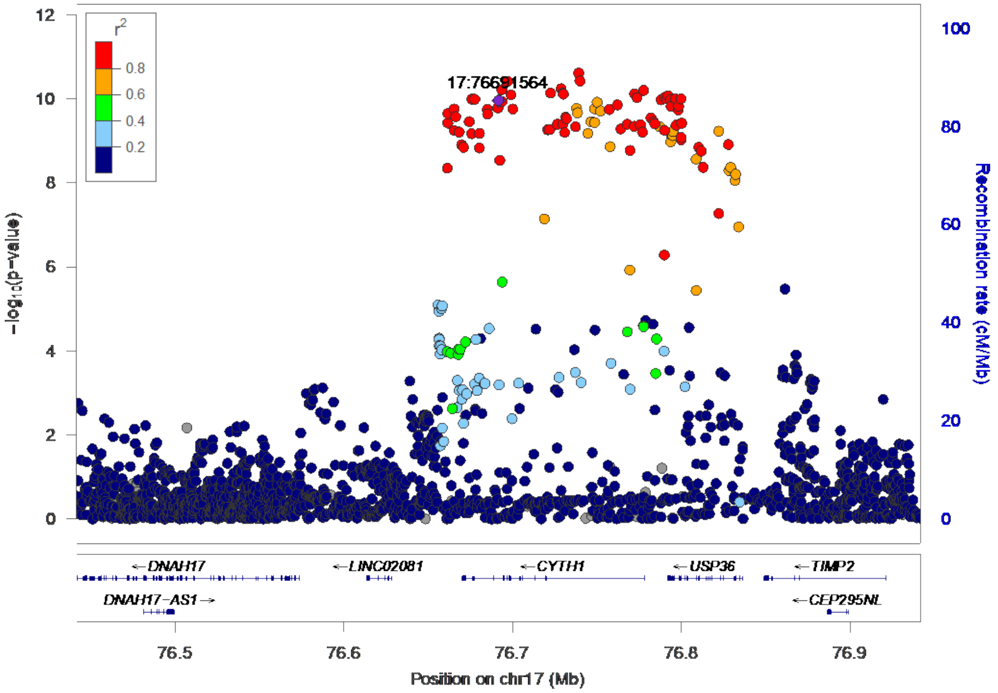
17:76691564
r2
0.8
0.6
0.4
0.2
−log10(p−value)
Recombination rate (cM/Mb)
DNAH17
LINC02081
CYTH1
USP36
TIMP2
DNAH17−AS1
CEP295NL
76.5
76.6
76.7
76.8
76.9
Position on chr17 (Mb)

p

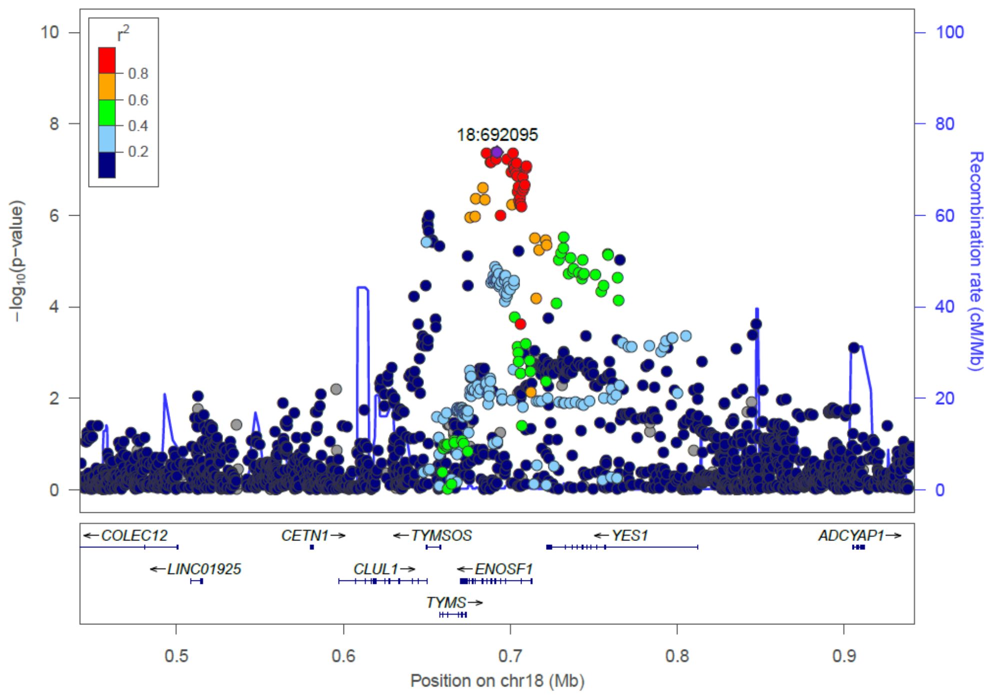

r2
0.8
0.6
0.4
0.2
18:692095
−log10(p-value)
Recombination rate (cM/Mb)
COLEC12
CETN1
TYMSOS
YES1
ADCYAP1
LINC01925
CLUL1
ENOSF1
TYMS
0.5
0.6
0.7
0.8
0.9
Position on chr18 (Mb)

q

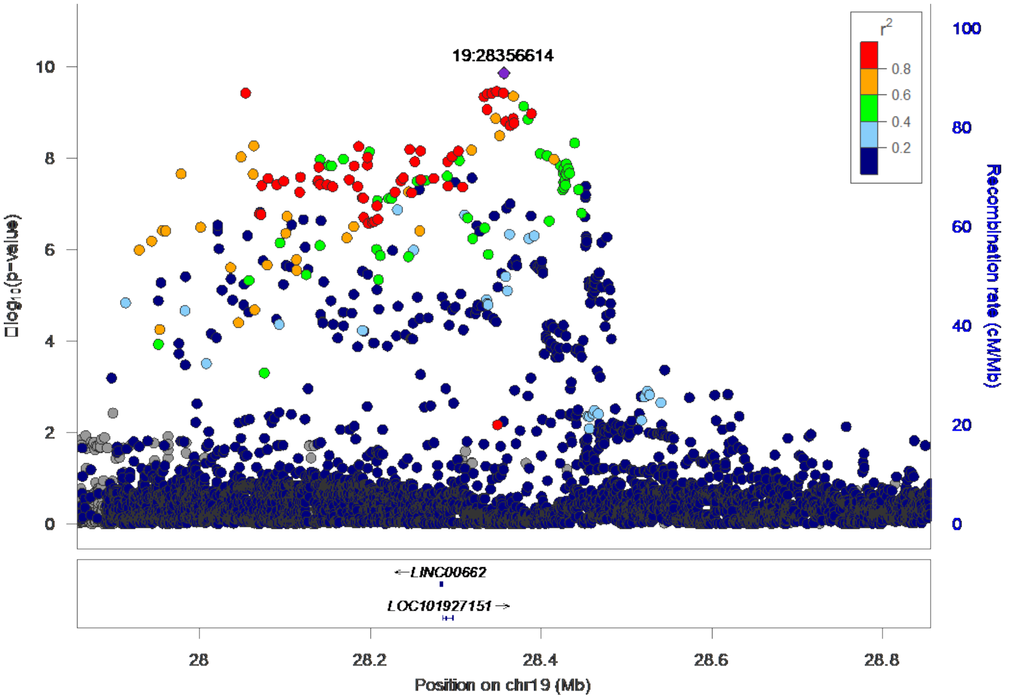

r2
0.8
0.6
0.4
0.2
19:28356614
log10(p-value)
Recombination rate (cM/Mb)
LINC00662
LOC101927151
28
28.2
28.4
28.6
28.8
Position on chr19 (Mb)

r

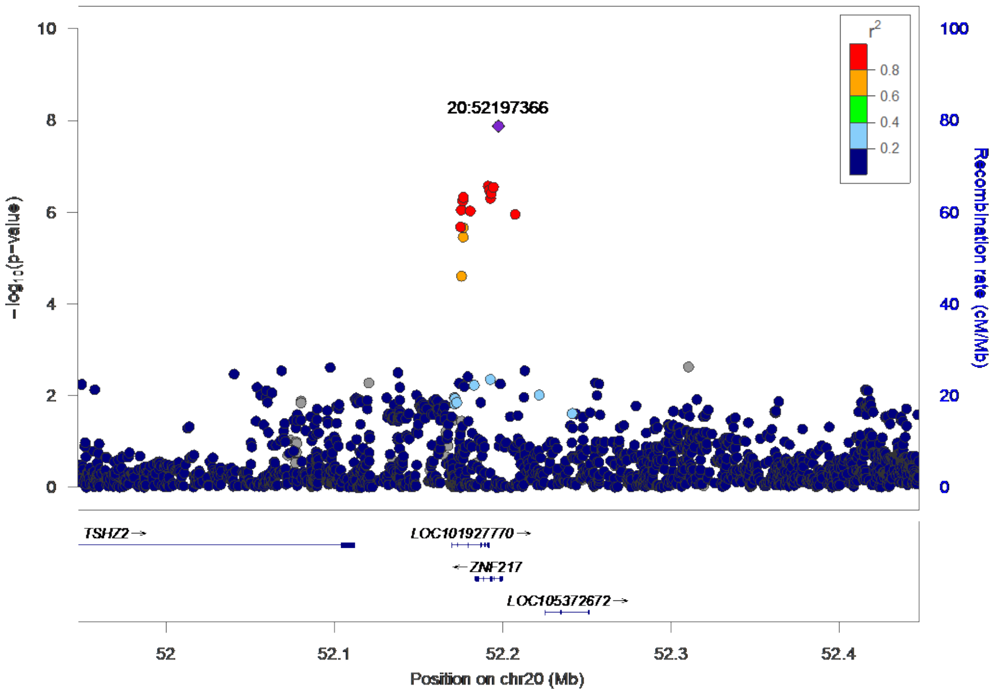

20:52197366
r2
0.8
0.6
0.4
0.2
10
8
6
4
2
0
100
80
60
40
20
−log10(p−value)
Recombination rate (cM/Mb)
TSHZ2→
LOC101927770→
←ZNF217
LOC105372672→
52
52.1
52.2
52.3
52.4
Position on chr20 (Mb)

s

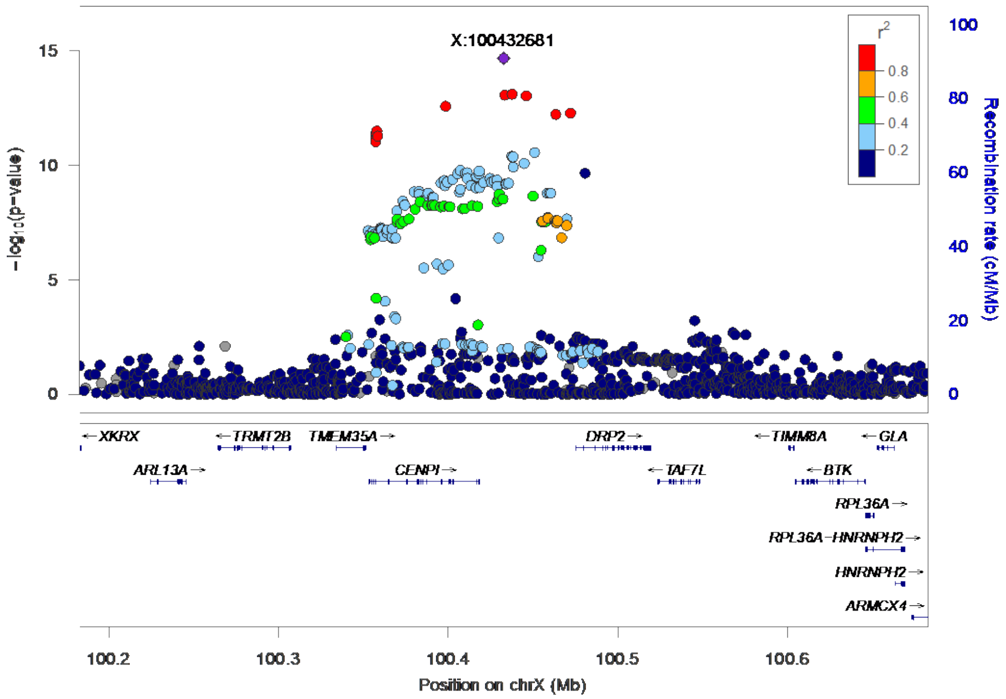

X:100432681
r2
0.8
0.6
0.4
0.2
15
10
5
0
100
80
60
40
20
−log10(p−value)
Recombination rate (cM/Mb)
←XKRX
←TRMT2B
TMEM35A→
DRP2→
←TIMM8A
←GLA
ARL13A→
CENPI→
←TAF7L
←BTK
RPL36A→
RPL36A−HNRNPH2→
HNRNPH2→
ARMCX4→
100.2
100.3
100.4
100.5
100.6
Position on chrX (Mb)

t

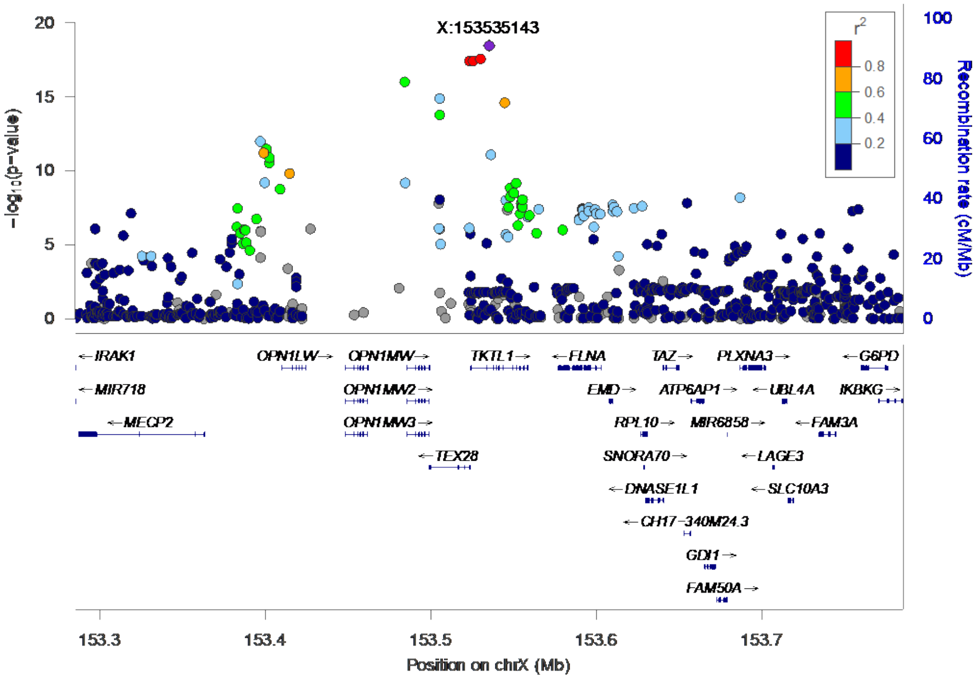
X:153535143
−log10(p-value)
Recombination rate (cM/Mb)
r2
0.8
0.6
0.4
0.2
IRAK1
MIR718
MECP2
OPN1LW
OPN1MW
OPN1MW2
OPN1MW3
TEX28
TKTL1
FLNA
EMD
RPL10
SNORA70
DNASE1L1
CH17-340M24.3
TAZ
ATP6AP1
MIR6858
GDI1
FAM50A
PLXNA3
UBL4A
LAGE3
SLC10A3
G6PD
IKBKG
FAM3A
153.3
153.4
153.5
153.6
153.7
Position on chrX (Mb)

u

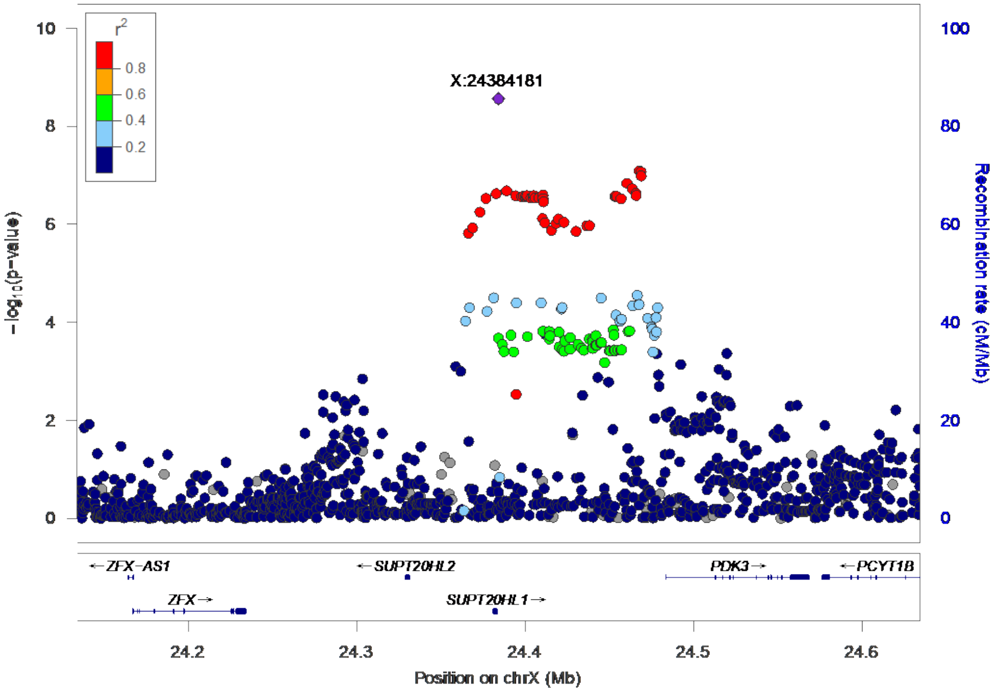
X:24384181
−log10(p-value)
Recombination rate (cM/Mb)
r2
0.8
0.6
0.4
0.2
ZFX-AS1
ZFX
SUPT20HL2
SUPT20HL1
PDK3
PCYT1B
24.2
24.3
24.4
24.5
24.6
Position on chrX (Mb)

v

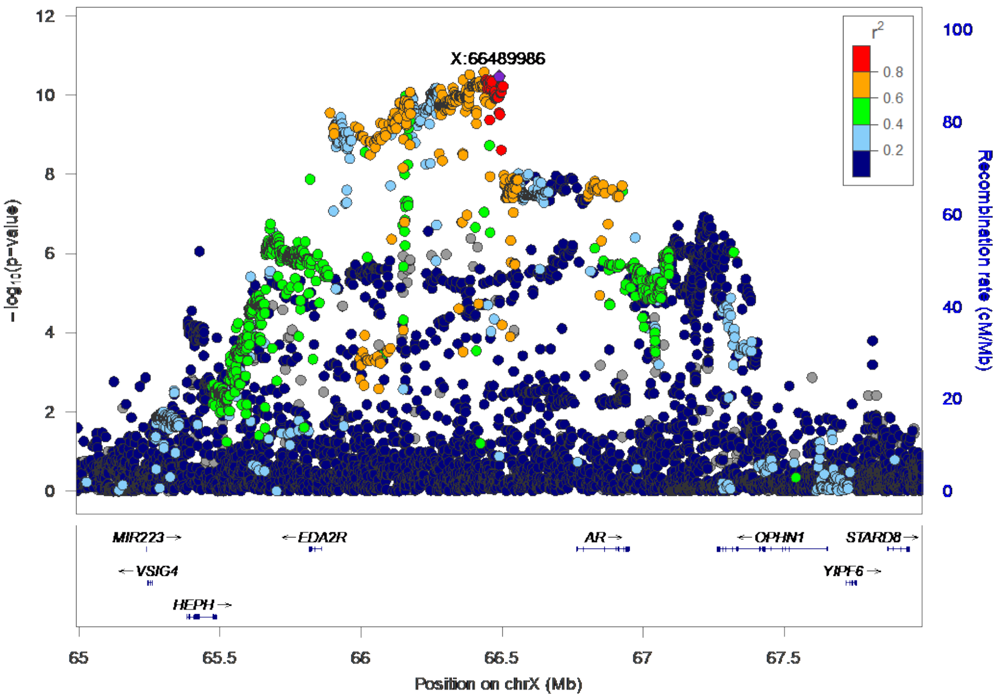
X:66489986
r2
0.8
0.6
0.4
0.2
−log10(p−value)
Recombination rate (cM/Mb)
MIR223→
←VSIG4
HEPH→
←EDA2R
AR→
←OPHN1
STARD8→
YIPF6→
Position on chrX (Mb)

Supplementary Fig. 2 | Conditional analysis plots showing independence of novel susceptibility loci in previously identified regions. Conditional analysis plots show previously identified susceptibility locus as a red triangle with a black outline and the novel susceptibility locus shown as a red circle with a black outline. A color gradient indicated in the figure legend shows the strength of LD with the novel susceptibility locus. A marker significantly associated with TGCT status ($P \leq 5 \times 10^{-8}$) in univariate logistic regression analysis that retained this level of significance in a model that also included the novel susceptibility locus is considered an independent marker and shown as a triangle; all other markers are shown as a circle. Novel independent regions appear as a cluster of markers in high LD with the novel susceptibility locus that are independent of any previously identified marker. **a**, novel marker rs7734992 (5:1280128) and independent regions (*TERT*). **b**, novel marker rs141079110 (6:33533625) and independent regions (*BAK1*). **c**, novel marker rs10976519 (9:779507) and independent regions (*DMRT1*). **d**, novel marker rs4898474 (X:153535143) and independent regions (*TKTL1*).

a

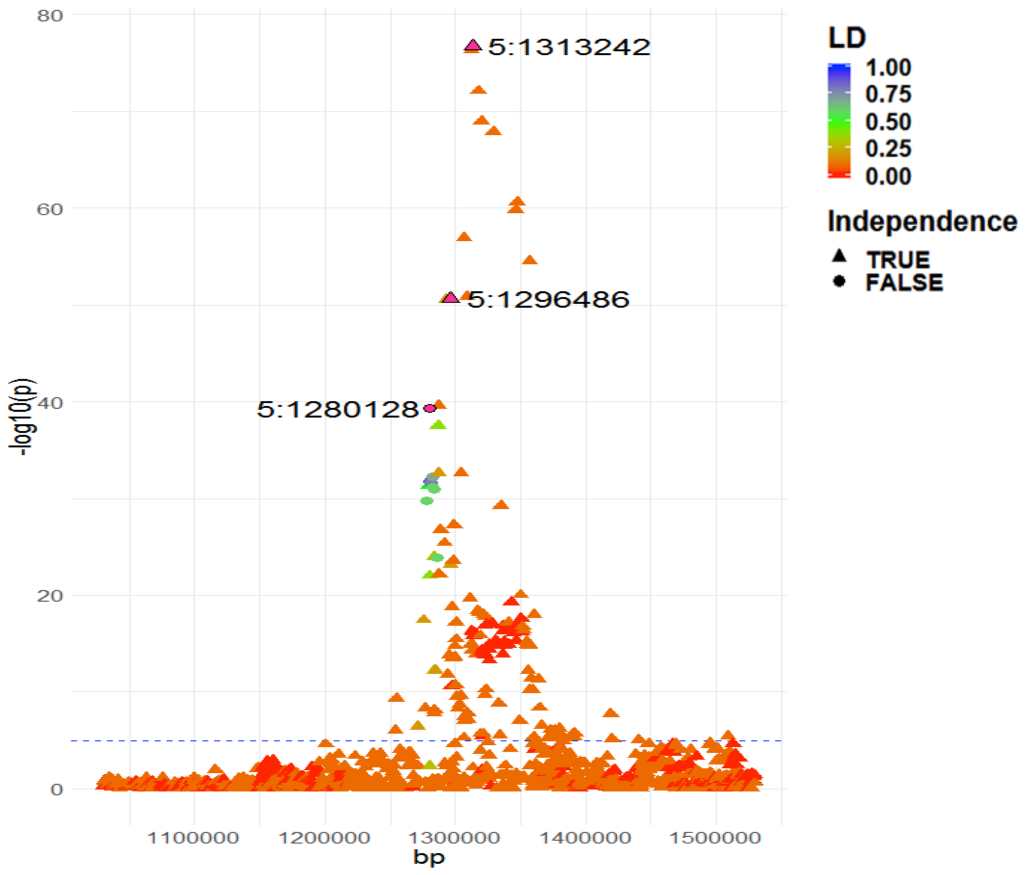

b

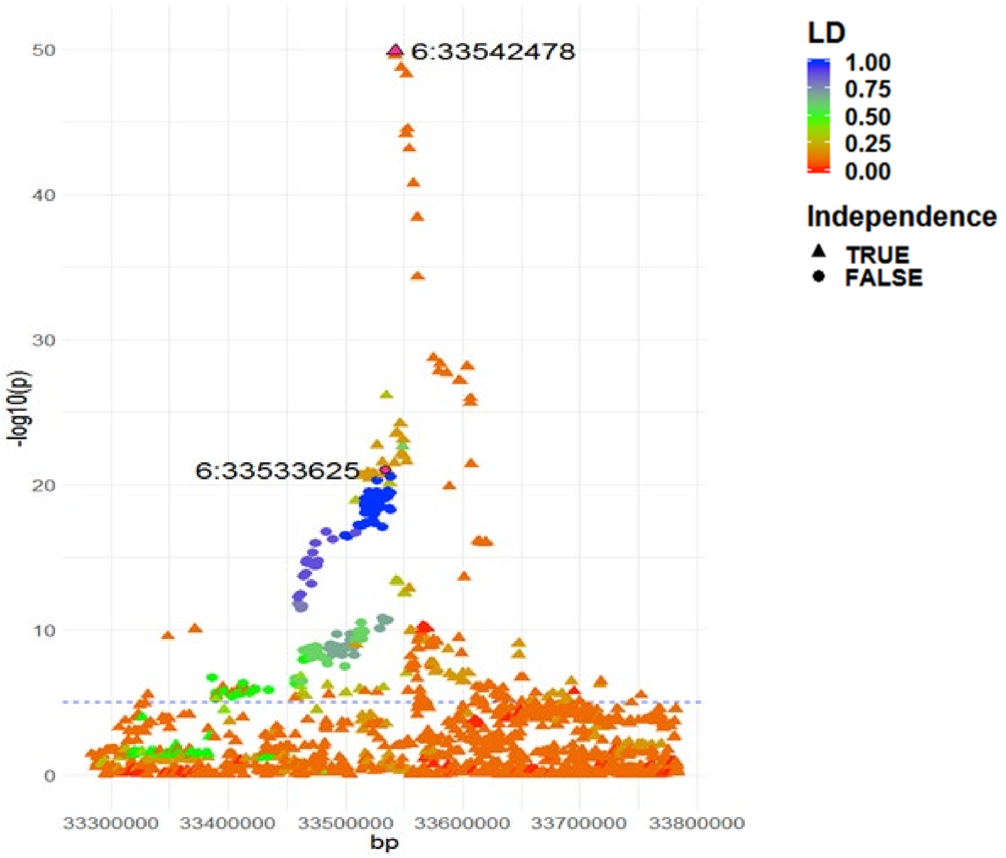
6:33542478
6:33533625
-log10(p)
50
40
30
20
10
0
33300000
33400000
33500000
33600000
33700000
33800000
bp
LD
1.00
0.75
0.50
0.25
0.00
Independence
TRUE
FALSE

c

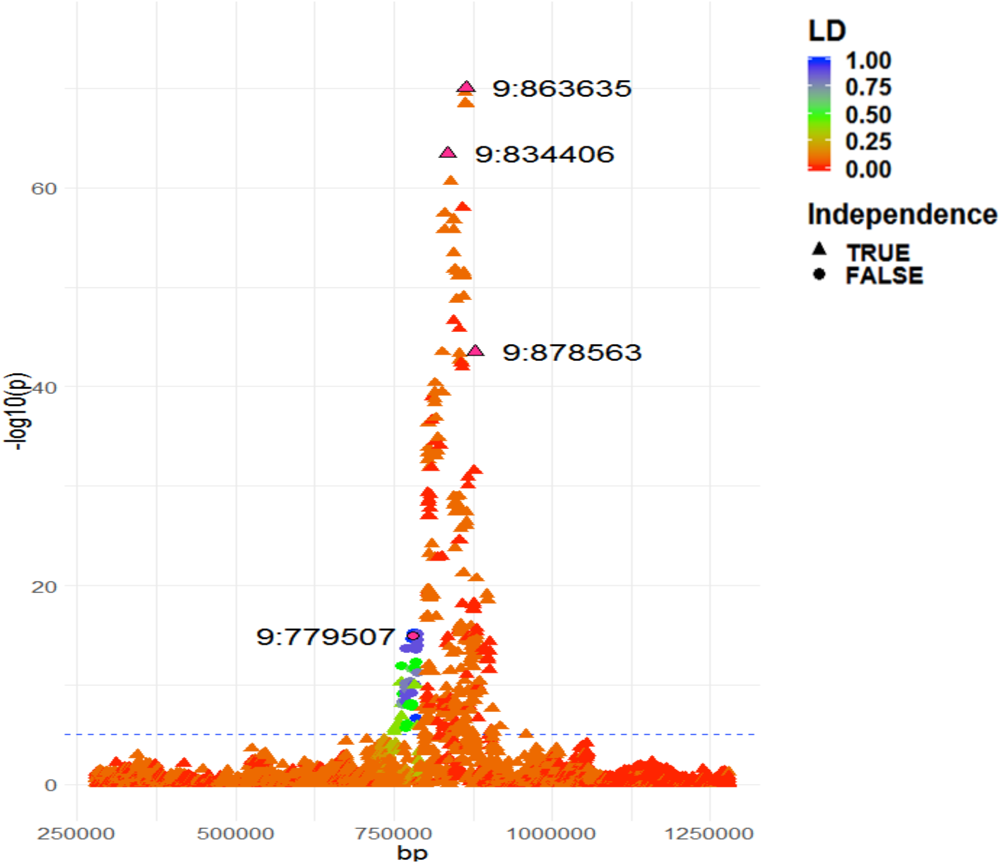
9:863635
9:834406
9:878563
9:779507
-log10(p)
60
40
20
0
250000
500000
750000
1000000
1250000
bp
LD
1.00
0.75
0.50
0.25
0.00
Independence
TRUE
FALSE

d

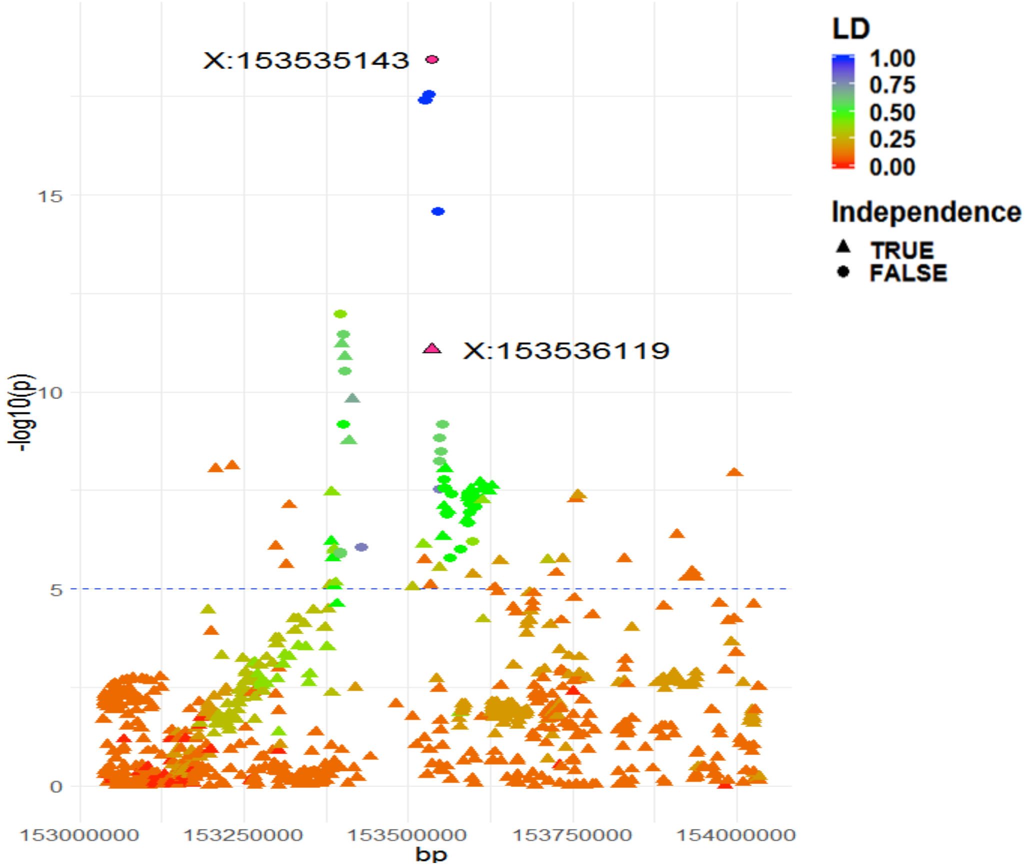

X:153535143
X:153536119
-log10(p)
15
10
5
0
153000000
153250000
153500000
153750000
154000000
bp
LD
1.00
0.75
0.50
0.25
0.00
Independence
TRUE
FALSE

.

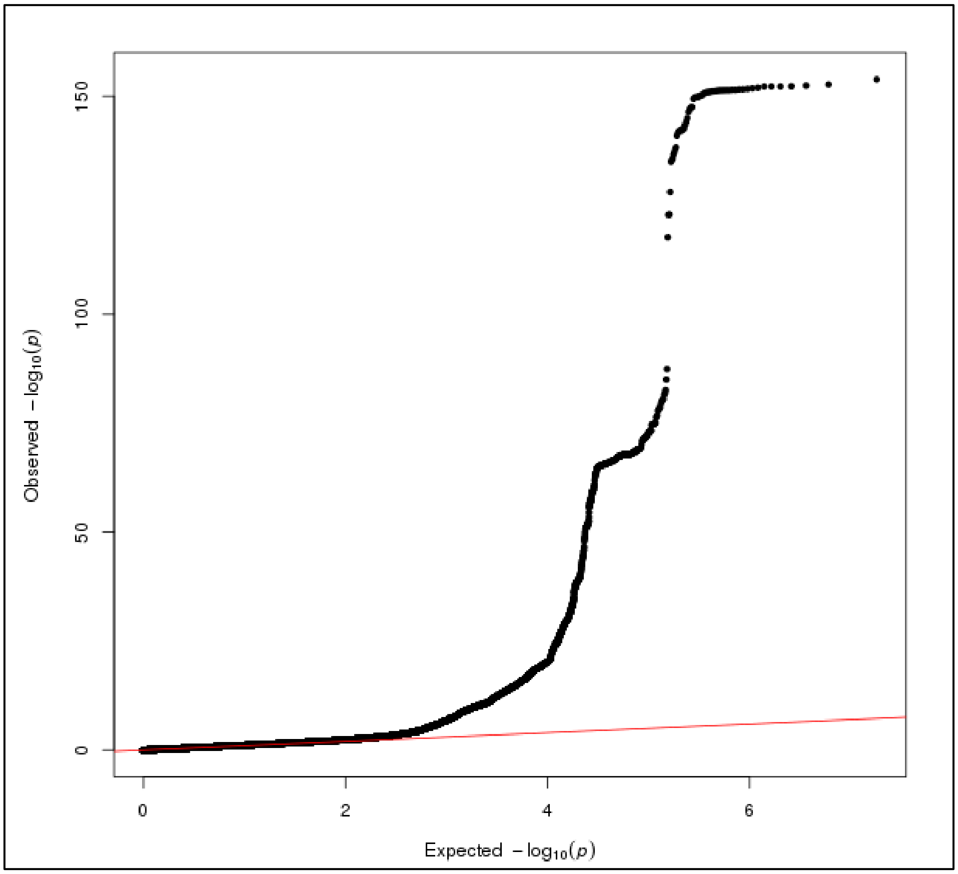

Supplementary Fig. 3 | Q-Q plot for TGCT meta-analysis.

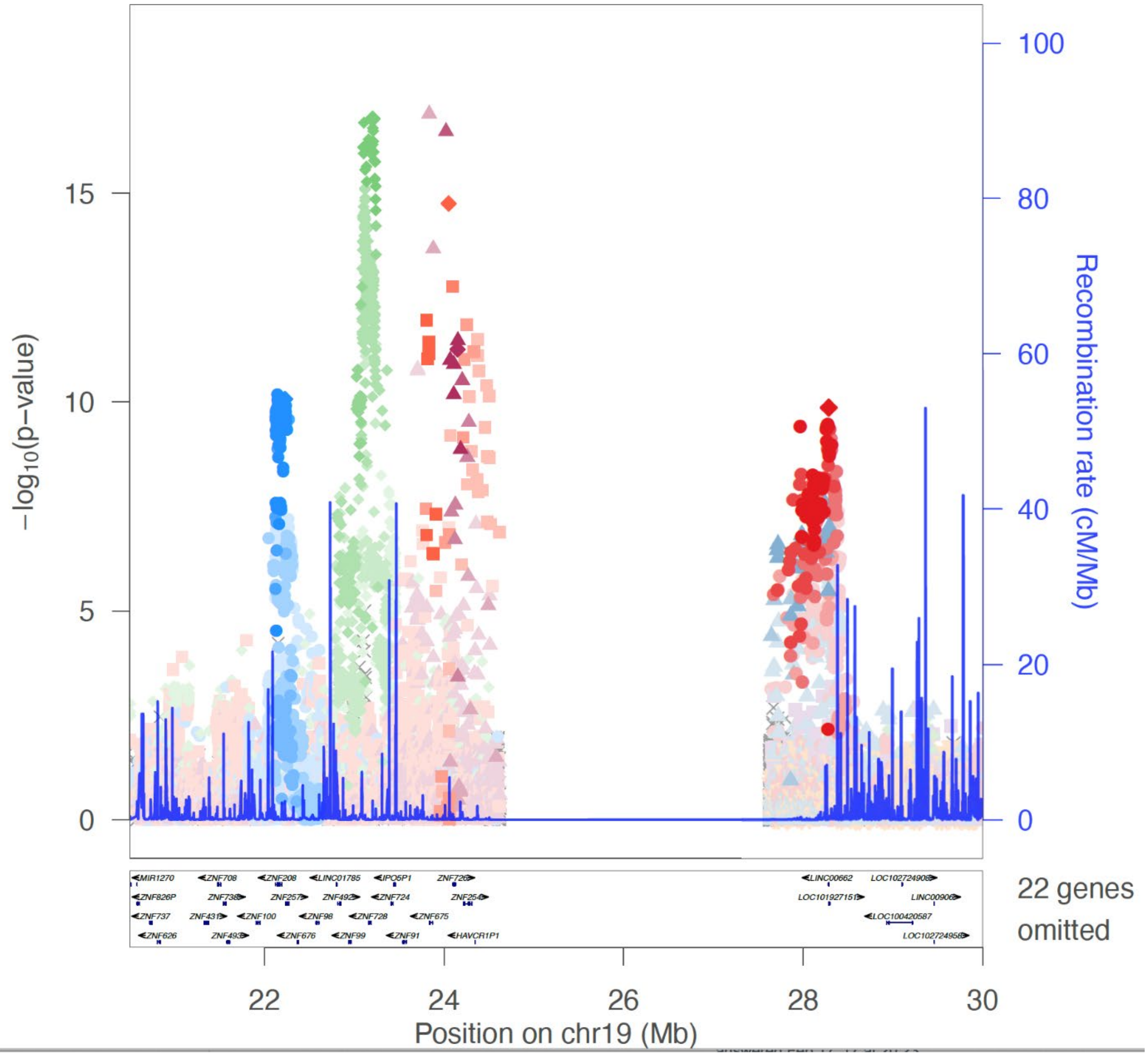

Supplementary Fig. 4 | Diagram of five independent nearby regions on chromosome 19 in associated with TGCT susceptibility. Additional genes in the region include *ZNF85, ZNF430, ZNF714, LINC00664, ZNF429, LOC400682, LOC641367, ZNF43, ZNF729, LOC101929124, LINC01233, LOC100996349, LINC01859, LINC01858, ZNF730, LINC01224, ZNF681, RPSAP58, LINC01532, UQCRFS1, LOC284395*

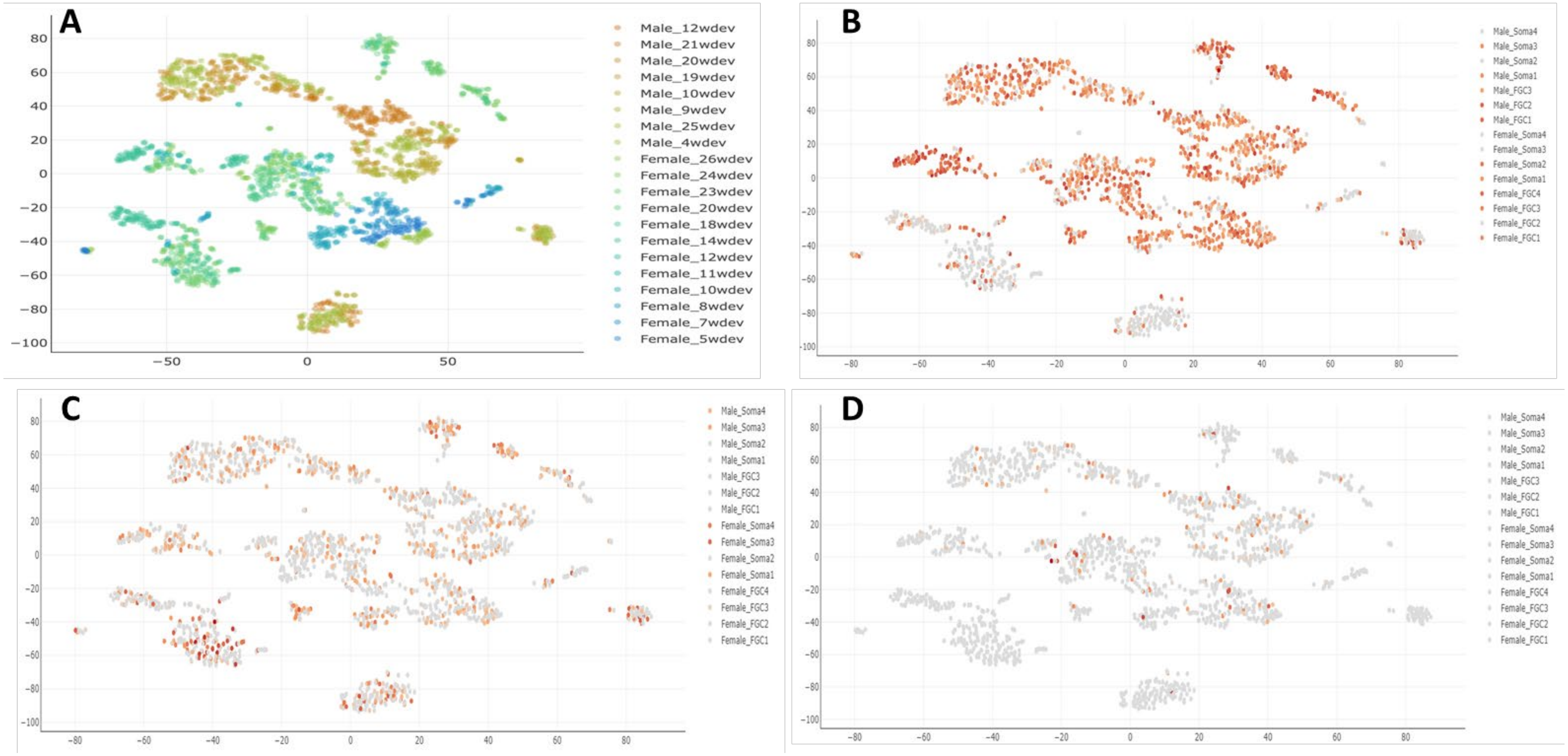

Supplementary Fig. 5 | Representative images of median of high, medium, and low expression levels in fetal gonads of potential target genes. Data from single cell analysis of human gonads at different stages of development was acquired from Li et al.[8] and analyzed in the ReproGenomics Viewer [9, 10]. Values representing the total transcript count ($\log_2$(TPM/10 + 1)) from all fetal gonadal cell populations are given. Tertile categories of expression values were used to assign expression as low (≤698), medium, 699-2348), or high ≥2349). **a**, map of cell types; **b**, high expression of *ZWILCH*, TPM=3949; **c**, medium expression of *LATS2*, TPM=1401; **d**, low expression of *CATSPER3*, TPM=207.

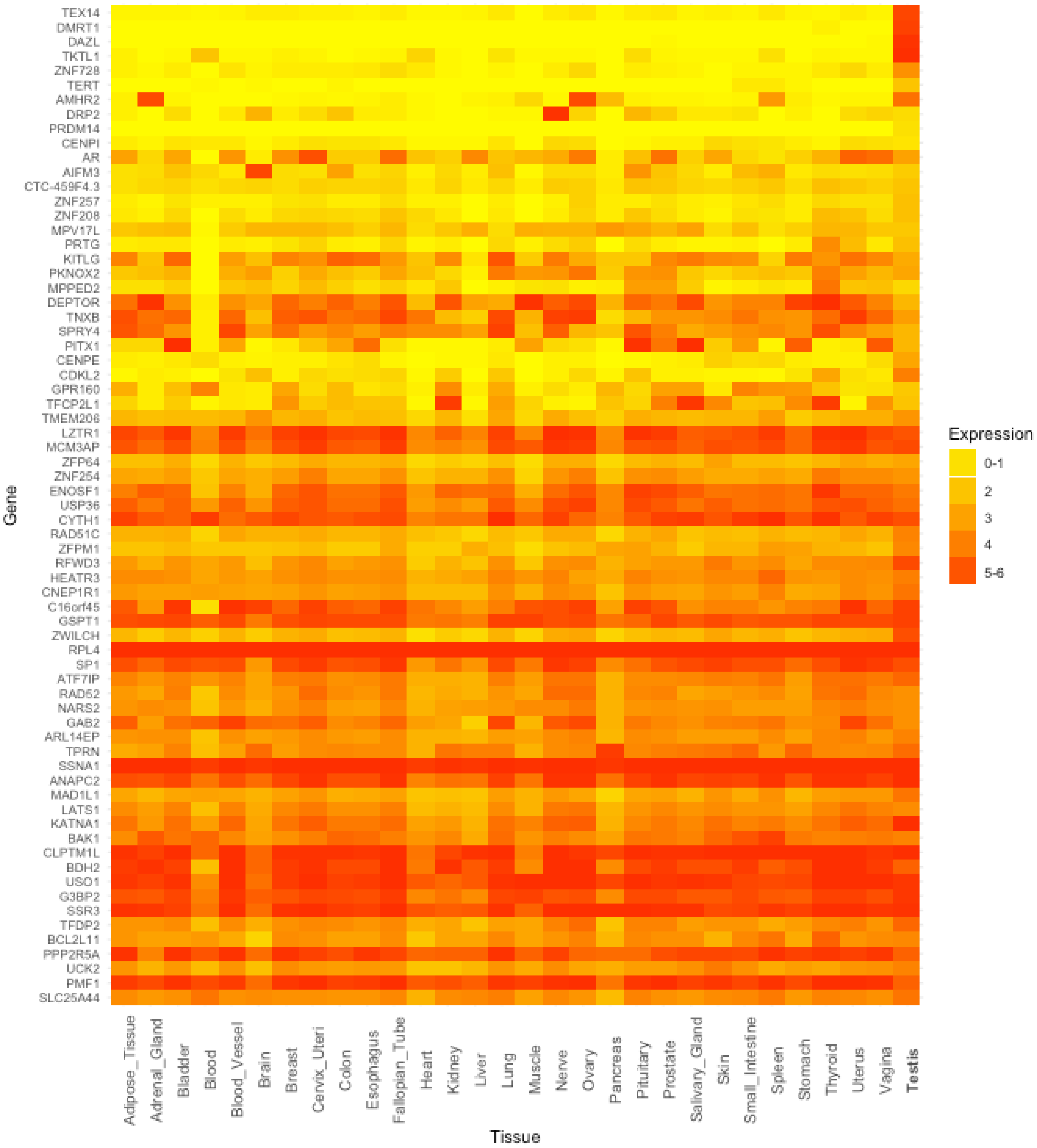

Supplementary Fig. 6 | Heatmap of tissue-specific gene expression. Expression levels (TPM) for 67 potential target genes are plotted for 30 tissue types available in GTEx. Testis-specific expression is in the right-most column. *DAZL*, *DMRT1*, and *TEX14* are testis-enriched; their expression in testis tissue is at least 1 TPM and is 5-fold or greater than in all other tissues; *TERT*, *TKTL1*, and *ZNF728* are testis-enhanced; their expression in testis tissue is at least 1 TPM and is 5-fold or greater than the average in all other tissues.

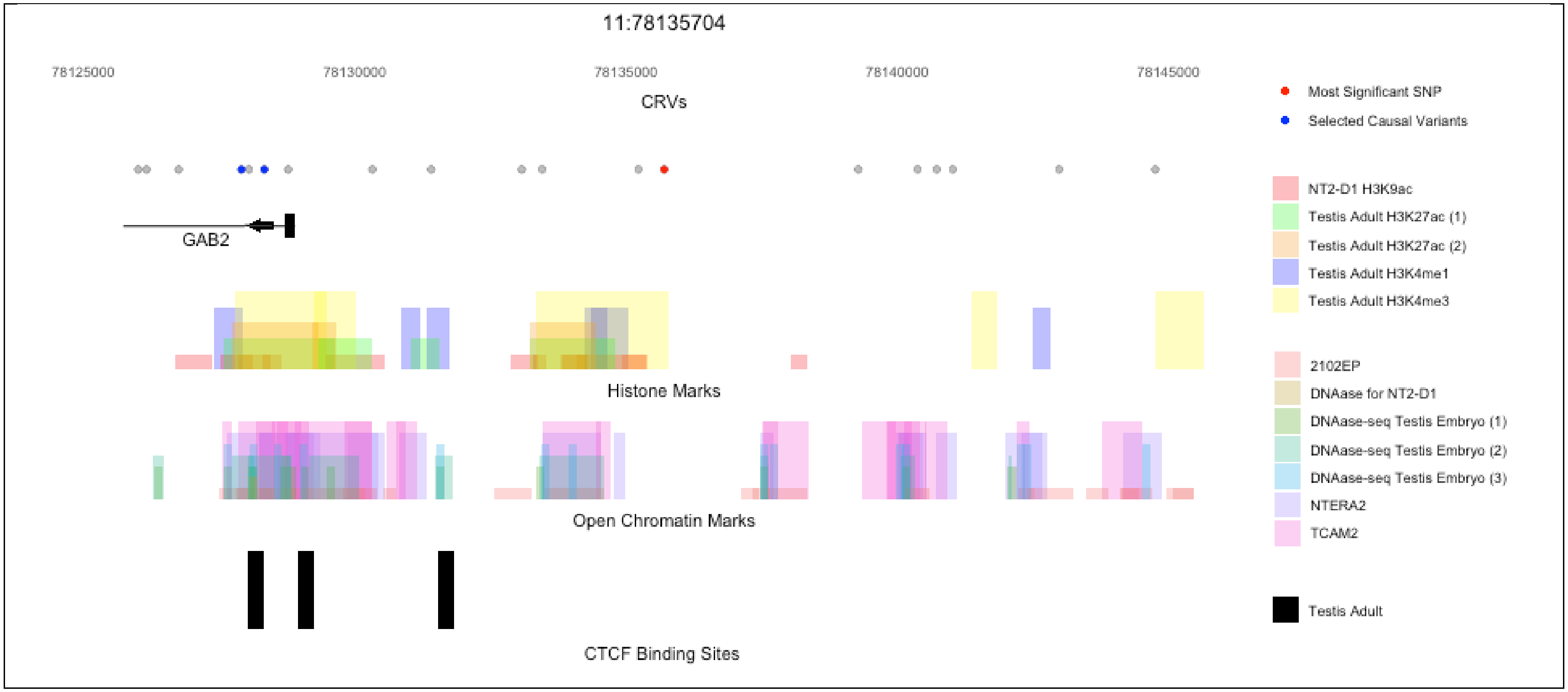

Supplementary Fig. 7 | Schematic example of functional annotation of novel loci. The functional annotation of GAB2 is shown in the panel. The top row indicates credible risk variants (CRV) for this marker, with the most statistically significant marker—the reference signal—rs990706 shown in red, and the inferred functional variants in blue. All data presented was included in the PAINTOR model. Each feature has a unique height, to allow for better discrimination. All the features overlap in the same region at the inferred functional variant on the left (rs869202), while the inferred functional variant on the right (rs881337) is selected due to features and high LD with rs869202 (r^2 = 1). The reference locus is intragenic, while the inferred functional variants map to *GAB2*.

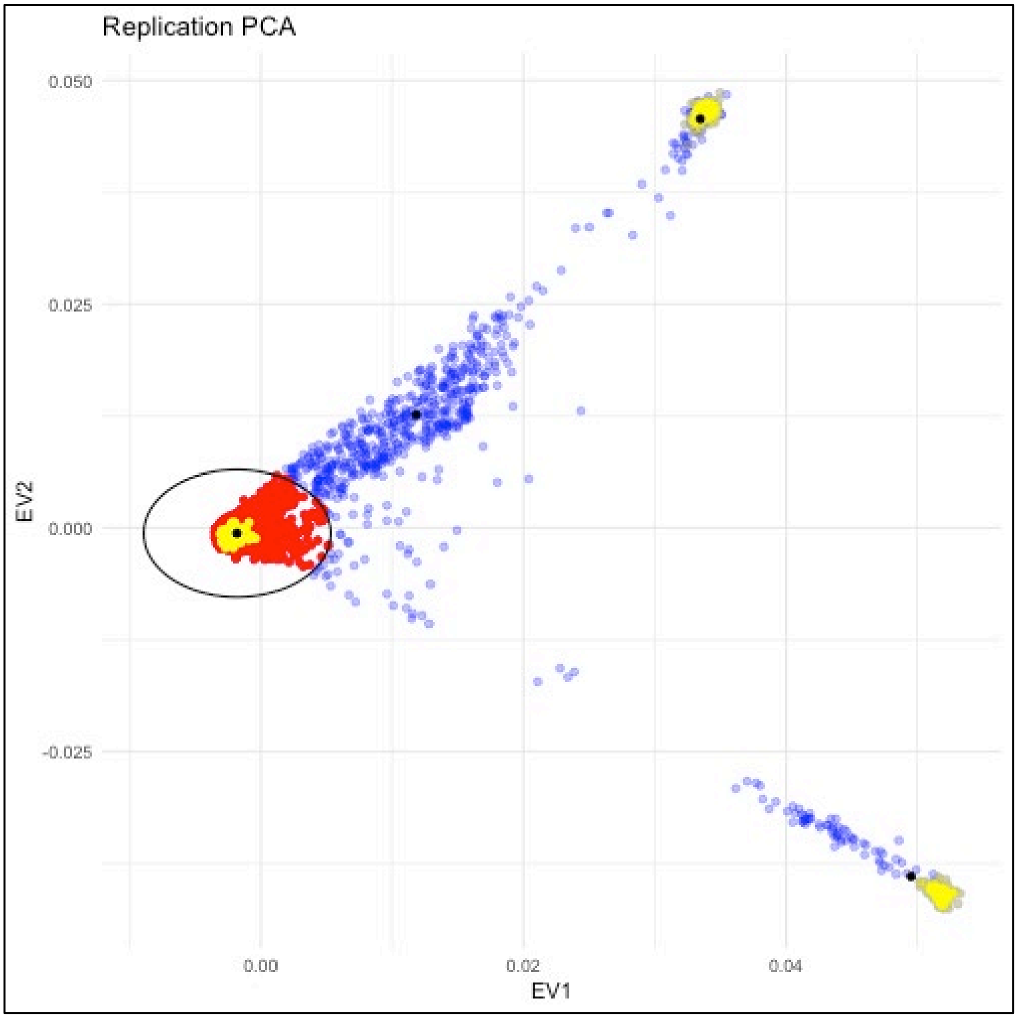

Supplementary Fig. 8 | Principal components analysis of TECAC samples with *de novo* genome-wide genotyping. Data from all genotyped subjects (n = 14,296) was decomposed into principal components and grouped by k-means clustering. 'European population' was defined as subjects within six standard deviations of the mean of the EUR cluster (indicated in red). 581 subjects from the analysis dataset (after removing relatives) that were outside of the cluster were removed from further analysis.

Supplemental Methods

Published genome-wide association studies included in the current meta-analysis

The data on 3557 and 13,970 men with and without testicular germ cell tumor (TGCT), respectively, from the five TGCT GWA studies from Denmark; Institute of Cancer Research; Karolinska Institutet, the Cancer Registry of Norway, and OsloMet – Oslo Metropolitan University (Norwegian-Swedish testicular cancer study); National Cancer Institute; and University of Pennsylvania were previously published individually and as a meta-analysis[2, 3, 4, 11, 12, 13]. The ascertainment of the subjects for those studies with IRB approvals is described in the referenced publications.

Additional genome-wide association studies included in the current meta-analysis

deCode Genetics

The Icelandic testicular cancer study is based on extensive genetic information on the Icelandic population, which has been previously described[6]. The whole genomes of 15,220 Icelanders were sequenced and variants were imputed into 151,677 Icelanders whose DNA had been genotyped with various Illumina SNP chips and phased using long-range phasing[14, 15]. Genealogical deduction of carrier status of 282,894 untyped relatives of chip-typed individuals further increased the sample size for association analysis. Information on testicular cancer in the genotyped individuals is from the population-based Icelandic Cancer Registry (ICR)[16]. A total of 300 Icelandic testicular cancer cases and 152,022 control subjects were used in the analysis. The number of variants tested was 13,171,680. The study was approved by the National Bioethics Committee of Iceland (ref. 12–122-V7). Written informed consent was obtained from all genotyped subjects.

The methods used for association testing in the Icelandic population have been described in detail[17]. To test for association between variants and cancer in the Icelandic study, logistic regression was used, treating disease status as the response and genotype counts as covariates. Other relevant covariates that might correlate with disease status were also included in the model as nuisance, for example, sex, county of birth, current age or age at death (first- and second-order terms included), blood sample availability for the individual, and an indicator function for the overlap of the lifetime of the individual with the time span of phenotype collection. To account for inflation in test statistics due to cryptic relatedness and stratification in the Icelandic population, we applied the method of linkage disequilibrium score regression[18]. The estimated correction factor for testicular cancer was 1.03.

UK Biobank

The UK biobank[7, 19] is a large cohort of 500,000 adult residents in the United Kingdom between 2006 and 2010, aged 40 to 69 at recruitment, extensively phenotyped and genotyped on the Axiom Array (Thermo Fisher Scientific) which contains >800,000 markers with the imputation conducted on a further 96 million markers. This prospective cohort contains 697 cases of testicular cancer of white ethnicity confirmed in the national cancer registry, including those diagnosed before joining the study and incidental cases. Data from ethnic minorities were not used in this analysis. Of the 697 cases, 395 were seminomas, 223 were non-seminomas whereas 79 had no histological record. To conduct genome-wide association analysis, we selected 8716 controls in the same cohort, matching with cases for year of birth and recruitment center (there were 22 recruitment centers distributed across the UK). We further imposed that controls were free from any cancer and were all white males. Genome-wide association analysis was conducted using PLINK2 separately for seminoma, non-seminoma and

unstratified, adjusting the leading 15 principal components calculated using the whole UK biobank cohort.

Testicular Cancer Consortium (TECAC)

For the sites included as part of the aggregated TECAC sample set, the TGCT case-control studies from Fred Hutchinson Cancer Research Center (The Adult Testicular Lifestyle and Blood Specimen study; ATLAS); Karolinska Institutet, Cancer Registry of Norway, and OsloMet – Oslo Metropolitan University; MD Anderson Cancer Center; University of Oslo; University of Pennsylvania (Testicular Cancer in Philadelphia Area Counties study; TestPAC); and University of Southern California are described in detail in [11, 13]. The control subjects from Karolinska Institutet, Cancer Registry of Norway, and OsloMet – Oslo Metropolitan University were 1st and 2nd degree relatives of TGCT cases not included in our analytic sample. Although these controls may carry a higher load of TGCT risk alleles compared to the normal population, we included them in our analyses to generate more robust estimates of association for the newly genotyped data despite the possibility of attenuation of association. Other sites not included in the prior studies are described below.

Princess Margaret Cancer Center

Princess Margaret has been treating TGCT since the late 1970’s using a Multidisciplinary clinical approach. Patients are drawn from the Toronto, neighboring greater Toronto cities and Northern Ontario communities. Beginning in 2008, the UHN Genito-Urinary (GU) Biobank at Princess Margaret has used a clinically integrated approach to collect blood samples on consenting TGCT patients who are seen in Testes-GU Clinic with IRB approval. Biobanked samples are stored along with clinical data for used in future research ethics board approved

studies. Princess Margaret sent a total of 537 TGCT cases including clinical data to University of Pennsylvania for DNA extraction and sequencing of which 380 were used for the germline genotyping and 31 for targeted genotyping.

Spanish National Cancer Research Centre (CNIO)

Forty Spanish candidate families with TGCTs fulfilling the criteria of having at least two affected members were selected (56 affected patients and 17 healthy parents)[20]. For the current study, probands only were used. A group of sporadic cases (n= 417) without family history of TGCTs was also collected, and finally a cohort of bilateral patients was recruited (n= 20). Recruitment of patients and collection of peripheral blood and clinical data were undertaken by a collaborative agreement with the Spanish Germ Cell Group and 35 oncology and urology departments from different institutions in our country. Ethical Committees from each hospital approved the study, and written informed consent was obtained from all participants prior to inclusion in the study. The control group consisted of 366 male Spanish healthy controls, present in the Spanish National Cancer Research Centre (CNIO) Biobank and registered at the Institute of Health Carlos III sample collection (C0001433).

Radboud University of Njimegen

All cases diagnosed with TGCT in the period 1997-2007 in the Eastern part of the Netherlands, covering a region of 1.3 inhabitants, were identified through the nationwide Netherlands Cancer Registry (NCR) held by the Netherlands Comprehensive Cancer Organization. Patients aged 15 to 40 years at diagnosis, alive at time of study, with a known address and able to read and understand the Dutch language, were included in the study. For these patients, data regarding patient and tumor characteristics, treatment and vital status were collected by well-trained data

managers from the NCR by consulting the medical files. In addition, patients were asked by their treating physician to donate a blood sample and to fill out a postal questionnaire. All patients that gave informed consent, received a lifestyle questionnaire. These questionnaires contained questions regarding lifestyle, medical history and family history of cancer. Blood samples were collected by regional Thrombosis Service centers. If needed, participants were visited at home for these blood collections. The study was approved by the institutional review board of Radboud University Medical Center.

Controls were obtained from the Nijmegen Biomedical Study[21], a population-based survey conducted by the Radboud University Medical Centre in Nijmegen. A random sample of max. N=800 in each gender-specific 5-year age group was drawn from the population register of the Municipality of Nijmegen. The total sample was 21,756. These selectees were invited to participate in a study on health and gene-environment interactions for disease. A postal questionnaire was also sent together with the invitation letter. The final page of the questionnaire was an informed consent form. The participants could indicate on the informed consent form whether they were willing to donate blood samples. Blood samples were taken by the regional Thrombosis service centers. If needed, participants were visited at home for these blood collections. The response to the questionnaire was 43% (N=9,350), and 69% (N=6,468) of the responders donated blood samples. Out of the 10,953 males, 4,307 filled out a questionnaire and 2,993 also donated blood. 1,033 males with blood samples were selected for a genome-wide association study. We retrieved genotyping data that passed quality control criteria for 906 males. In a second, third and fourth phase of the study, participants were invited to come to the University for anthropometric measurements, non-invasive blood vessel measurements, blood and urine collection and were asked to fill out additional questionnaire forms. Part of the males has participated in these additional phases. Applied selection criteria

were: minimum age of 18 years, country of residence: Netherlands (Nijmegen) and capable of reading a Dutch questionnaire.

University of Groningen

University Medical Center Groningen, The Netherlands is a large referral University Medical Center has treating testicular germ cell cancer since 1977 using a multidisciplinary clinical team approach. Patients are referred from the region of two million people. Starting in 1998, we registered TGCT patients in the institutional testicular cancer database. Starting in 2001, we approached TGCT patients to participate in Ethics Committee approved studies in which, after written informed consent, clinical data and blood samples were collected and stored in our institutional data biobank. The University Medical Center Groningen sent a total of 510 cases including clinical data and 189 family members to the University of Pennsylvania.

University of Leeds

The Leeds Testicular Cancer Study has recruited testicular cancer cases, parents and controls in the UK between 1998 and 2001 (ethical approval 98/094) and between 2013 and 2018 (ethical approval 12/YH/0532). Of these recruits, 442 cases and 273 controls were included in the TECAC Replication cohort. Thirty-four samples were duplicated for quality control purposes. DNA was extracted from blood samples at the University of Leeds and sent to the University of Pennsylvania for centralized genotyping with samples from other TECAC groups.

University of Padova

Subjects were selected from participants in the IRB-approved protocol #2400P from the University of Padova, Department of Medicine, Unit of Andrology and Reproductive Medicine, entitled “Genetic predisposition to testicular cancer and male infertility.” Cases were referred for sperm cryopreservation after orchiectomy for TGCT, and controls were males without TGCT recruited among men who were referred for standard semen analysis, and TGCT was excluded by andrological examination and testicular ultrasound. All cases and controls were Caucasian from the northeast of Italy and informed consent was obtained from each subject. Subgroups of this cohort of men with TGCT and controls has been reported in previous studies of genetic polymorphisms in candidate genes[22, 23, 24, 25].

University of Turin

EPSAM (Esposizioni Postnatali e SAlute Maschile) is a population-based case-control study that recruited among residents of the Province of Turin, Piedmont Region, Italy[26, 27]. Patients aged 15 to 54 years and diagnosed with TGCT between 1997 and 2008 were recruited with corresponding controls. Cases and controls were contacted using two sources. The first source included men who were diagnosed with TGCT at the main hospital of the city of Turin and invited to participate in the study through their oncologist. Controls were selected among patients admitted at the same hospital between 2008 and 2009 for non-neoplastic diseases unrelated to hormonal factors and infertility, frequency matched to cases on year of birth and residence. The second source was based on general practitioners of the Turin Province whose patients’ list included at least one man with a history of orchiectomy for testicular cancer (ICD-9 CM surgical procedure codes: 623–624; ICD-9 CM diagnostic code: 186). Controls were randomly selected among the patients of the same GP of the corresponding case, matching on year of birth. Thus, all cases and controls were resident in the Province of Turin, but they were either contacted though the hospital (first source) or their general practitioner (second source).

Between 2008 and 2010 cases and controls received a postal questionnaire and a kit for self-collection of a saliva sample for DNA extraction[28]. The study included 255 cases and 459 controls, of which 206 cases and 323 controls contributed with DNA to the Testicular Cancer Consortium.

University of Ulm

Four hundred and forty-seven Croatian men with TGCTs were ascertained from the Departments of Oncology and Urology at the Clinical Hospital Centre Zagreb, Croatia. In addition, 446 male individuals from the Croatian population, who were at least 50 years old with no personal or family history of cancer, were selected from blood donors at the Croatian Institute for Transfusion Medicine, Zagreb, Croatia, as previously described[29, 30]. Written informed consent was obtained from each individual after detailed explanation of the purpose of the study. The study was approved by the ethics committees of the Clinical Hospital Centre Zagreb and the Croatian Institute for Transfusion Medicine.

Yale University

Details of the study have been described previously[31]. Briefly, participants for this population-based case–control study were recruited between 2006 and 2010 among male residents of CT and MA. The incident cases included newly diagnosed patients with TGCT (International Classification of Diseases for Oncology Morphology Codes 9906–9910) identified using the Yale Comprehensive Cancer Center's Rapid Case Ascertainment Shared Resource (RCA) and the Massachusetts Cancer Registry. The eligibility criteria for cases in the study included having a histologically confirmed TGCT (Stage 0–IV) diagnosed during 2006–2010, no previous cancer diagnoses except for non-melanoma skin cancer, being a male resident of CT or MA and

between the ages of 18 and 55 at diagnosis, alive and competent to answer questions at the time of interview, and able to speak English in order to complete the interview. A total of 356 histologically confirmed incident TGCT patients were identified and 308 cases provided blood samples for the study. A total of 513 population-based controls were selected with using a random digit dialing and 323 of them provided blood samples that were included in this analysis. The male controls were frequency-matched to the cases based on age (±5 years) and state. Efforts were made to frequency match the cases and controls with a 1:1 ratio by adjusting the number of controls randomly selected in each age stratum during the study period. The study was approved by the IRBs at both Yale and Harvard Universities, and by the Human Investigation Committee (HIC) at the Department of Public Health at the States of the CT and MA, at the Dana Farber Cancer Institute, and the 28 participating hospitals in Connecticut.

In-person interviews were conducted by trained interviewers, either in the participants' homes or at locations convenient for the subjects after approval by each subject's hospital and physician and having received consents from the study subjects. A standardized and structured questionnaire was used to collect information on demographic and lifestyle factors, occupation history, past medical history, physical activity, family cancer history, diet and nutrient intakes. Birth certificates were obtained for information on birth outcomes including birth weight, birth length and undescended testis. Following the completion of the in-person interview, at least 5cc venous blood was collected from each of the 308 cases and 323 controls and stored at -84°C until laboratory analyses.

References

1. Dalgaard MD*, et al.* A genome-wide association study of men with symptoms of testicular dysgenesis syndrome and its network biology interpretation. *J Med Genet* **49**, 58-65 (2012).

2. Kristiansen W*, et al.* Two new loci and gene sets related to sex determination and cancer progression are associated with susceptibility to testicular germ cell tumor. *Hum Mol Genet* **24**, 4138-4146 (2015).

3. Schumacher FR*, et al.* Testicular germ cell tumor susceptibility associated with the UCK2 locus on chromosome 1q23. *Hum Mol Genet* **22**, 2748-2753 (2013).

4. Kanetsky PA*, et al.* A second independent locus within DMRT1 is associated with testicular germ cell tumor susceptibility. *Hum Mol Genet* **20**, 3109-3117 (2011).

5. Litchfield K*, et al.* Identification of four new susceptibility loci for testicular germ cell tumour. *Nat Commun* **6**, 8690 (2015).

6. Gudbjartsson DF*, et al.* Large-scale whole-genome sequencing of the Icelandic population. *Nat Genet* **47**, 435-444 (2015).

7. Bycroft C*, et al.* The UK Biobank resource with deep phenotyping and genomic data. *Nature* **562**, 203-209 (2018).

8. Li L*, et al.* Single-Cell RNA-Seq Analysis Maps Development of Human Germline Cells and Gonadal Niche Interactions. *Cell Stem Cell* **20**, 858-873.e854 (2017).

9. Darde TA*, et al.* The ReproGenomics Viewer: a multi-omics and cross-species resource compatible with single-cell studies for the reproductive science community. *Bioinformatics* **35**, 3133-3139 (2019).

10. Darde TA*, et al.* The ReproGenomics Viewer: an integrative cross-species toolbox for the reproductive science community. *Nucleic Acids Res* **43**, W109-116 (2015).

11. Chung CC*, et al.* Meta-analysis identifies four new loci associated with testicular germ cell tumor. *Nat Genet* **45**, 680-685 (2013).

12. Ruark E*, et al.* Identification of nine new susceptibility loci for testicular cancer, including variants near DAZL and PRDM14. *Nat Genet* **45**, 686-689 (2013).

13. Wang Z*, et al.* Meta-analysis of five genome-wide association studies identifies multiple new loci associated with testicular germ cell tumor. *Nat Genet* **49**, 1141-1147 (2017).

14. Kong A*, et al.* Detection of sharing by descent, long-range phasing and haplotype imputation. *Nat Genet* **40**, 1068-1075 (2008).

15. Kong A*, et al.* Parental origin of sequence variants associated with complex diseases. *Nature* **462**, 868-874 (2009).

16. Sigurdardottir LG*, et al.* Data quality at the Icelandic Cancer Registry: comparability, validity, timeliness and completeness. *Acta Oncol* **51**, 880-889 (2012).

17. Helgason H*, et al.* Loss-of-function variants in ATM confer risk of gastric cancer. *Nat Genet* **47**, 906-910 (2015).

18. Bulik-Sullivan BK*, et al.* LD Score regression distinguishes confounding from polygenicity in genome-wide association studies. *Nat Genet* **47**, 291-295 (2015).

19. Sudlow C*, et al.* UK biobank: an open access resource for identifying the causes of a wide range of complex diseases of middle and old age. *PLoS Med* **12**, e1001779 (2015).

20. Paumard-Hernandez B*, et al.* Whole exome sequencing identifies PLEC, EXO5 and DNAH7 as novel susceptibility genes in testicular cancer. *Int J Cancer* **143**, 1954-1962 (2018).

21. Galesloot TE*, et al.* Cohort Profile: The Nijmegen Biomedical Study (NBS). *Int J Epidemiol* **46**, 1099-1100j (2017).

22. Ferlin A, Zuccarello D, Zuccarello B, Chirico MR, Zanon GF, Foresta C. Genetic alterations associated with cryptorchidism. *JAMA* **300**, 2271-2276 (2008).

23. Ferlin A, Ganz F, Pengo M, Selice R, Frigo AC, Foresta C. Association of testicular germ cell tumor with polymorphisms in estrogen receptor and steroid metabolism genes. *Endocr Relat Cancer* **17**, 17-25 (2010).

24. Ferlin A, Pengo M, Pizzol D, Carraro U, Frigo AC, Foresta C. Variants in KITLG predispose to testicular germ cell cancer independently from spermatogenic function. *Endocr Relat Cancer* **19**, 101-108 (2012).

25. Rocca MS*, et al.* Copy number variations of E2F1: a new genetic risk factor for testicular cancer. *Endocr Relat Cancer* **24**, 119-125 (2017).

26. Richiardi L*, et al.* Lifetime growth and risk of testicular cancer. *Int J Cancer* **135**, 695-701 (2014).

27. Moirano G*, et al.* Postnatal risk factors for testicular cancer: The EPSAM case-control study. *Int J Cancer* **141**, 1803-1810 (2017).

28. Grasso C*, et al.* Subfertility and Risk of Testicular Cancer in the EPSAM Case-Control Study. *PLoS One* **11**, e0169174 (2016).

29. Lessel D*, et al.* Replication of genetic susceptibility loci for testicular germ cell cancer in the Croatian population. *Carcinogenesis* **33**, 1548-1552 (2012).

30. AlDubayan SH*, et al.* Association of Inherited Pathogenic Variants in Checkpoint Kinase 2 (CHEK2) With Susceptibility to Testicular Germ Cell Tumors. *JAMA Oncol* **5**, 514-522 (2019).

31. Li N*, et al.* Muscle-building supplement use and increased risk of testicular germ cell cancer in men from Connecticut and Massachusetts. *Br J Cancer* **112**, 1247-1250 (2015).
