# Supplementary material for: Identification of 22 susceptibility loci associated with testicular germ cell tumors
Source: Nat Commun. 2021 Jul 23;12:4487. doi: 10.1038/s41467-021-24334-y (PMC8302763; doi:10.1038/s41467-021-24334-y)
Supplement: Supplementary file 2 — Description of Additional Supplementary Files [file 41467_2021_24334_MOESM2_ESM.pdf]

Description of Additional Supplementary Files

File Name: Supplementary Data 1
Description: Detailed information on new independent top signals for TGCT reaching genome-wide significance

File Name: Supplementary Data 2
Description: Summary information for previously published TGCT susceptibility loci.

File Name: Supplementary Data 3
Description: Detailed information on previously published TGCT susceptibility markers

File Name: Supplementary Data 4
Description: Comparison of TGCT identified susceptibility markers with other GWAS

File Name: Supplementary Data 5
Description: Evaluation of potential target genes contained in gene regions corresponding to top signals on autosomes

File Name: Supplementary Data 6
Description: CRVs annotated at gene coding regions or splice sites.

File Name: Supplementary Data 7
Description: Results of TGCT eQTL colocalization analysis

File Name: Supplementary Data 8
Description: Data visualizing the annotation of top signals with information from 36 ENCODE and four in-house sources (Supplementary Table 9 and Supplementary Data 7) can be found at https://genome.ucsc.edu/s/jpluta/TECAC2020.

File Name: Supplementary Data 9
Description: Credible risk variants and results of PAINTOR analysis to identify TGCT-associated potential functional variants

File Name: Supplementary Data 10
Description: Summary statistics for the top 10,000 signals
