# Supplementary material for: Identification of 22 susceptibility loci associated with testicular germ cell tumors
Source: Nat Commun. 2021 Jul 23;12:4487. doi: 10.1038/s41467-021-24334-y (PMC8302763; doi:10.1038/s41467-021-24334-y)
Supplement: Supplementary file 4 — Reporting Summary [file 41467_2021_24334_MOESM4_ESM.pdf]

nature research

Corresponding author(s): Nathanson, Katherine

Last updated by author(s): Feb 8, 2021

Reporting Summary

Nature Research wishes to improve the reproducibility of the work that we publish. This form provides structure for consistency and transparency in reporting. For further information on Nature Research policies, see our Editorial Policies and the Editorial Policy Checklist.

Statistics

For all statistical analyses, confirm that the following items are present in the figure legend, table legend, main text, or Methods section.

n/a	Confirmed	
☐	☒	The exact sample size (*n*) for each experimental group/condition, given as a discrete number and unit of measurement
☐	☒	A statement on whether measurements were taken from distinct samples or whether the same sample was measured repeatedly
☐	☒	The statistical test(s) used AND whether they are one- or two-sided<br>*Only common tests should be described solely by name; describe more complex techniques in the Methods section.*
☐	☒	A description of all covariates tested
☐	☒	A description of any assumptions or corrections, such as tests of normality and adjustment for multiple comparisons
☐	☒	A full description of the statistical parameters including central tendency (e.g. means) or other basic estimates (e.g. regression coefficient) AND variation (e.g. standard deviation) or associated estimates of uncertainty (e.g. confidence intervals)
☐	☒	For null hypothesis testing, the test statistic (e.g. *F*, *t*, *r*) with confidence intervals, effect sizes, degrees of freedom and *P* value noted<br>*Give P values as exact values whenever suitable.*
☒	☐	For Bayesian analysis, information on the choice of priors and Markov chain Monte Carlo settings
☐	☒	For hierarchical and complex designs, identification of the appropriate level for tests and full reporting of outcomes
☐	☒	Estimates of effect sizes (e.g. Cohen's *d*, Pearson's *r*), indicating how they were calculated

Our web collection on statistics for biologists contains articles on many of the points above.

Software and code

Policy information about availability of computer code

Data collection	*Provide a description of all commercial, open source and custom code used to collect the data in this study, specifying the version used OR state that no software was used.*
Data analysis	PLINK1.09, for quality control and general manipulation of genotype data, PRS scoring; EIGENSOFT v6.1.4 for principle component analysis of genomic ancestry; SNPTEST v2.5 for association testing; PAINTOR v3.0 for functional analysis; METAL (release 2018-08-28) for meta-analysis; Michigan Imputation Server v1.0.2 which implements minimac4 v1.0.0 and eagle2 v24.1 for imputation against HHRC r1.1; GCTA v1.26.0 for independence analysis and identification of CRVs; LocusZoom 1.4 for plotting of snps with linkage disequilibrium; MAGMA 1.07 for gene set analysis, implemented via FUMA v1.3.5e; UCSC LiftOver for conversion of grch38 coordinates to grch37; FastQC v0.11.9 for quality control of FASTA files; bowtie2 v2.4.2 for alignment; ANNOVAR r. 2019-10-24 for annotation; CHiCAGO v3.12 for Hi-C interactions and to assess enrichment of genomic features in promoter interacting regions; SNiPA v3.4 for identify proxy snps and annotation; STRINGR v11.0 for network annotation and visualization. Custom scripts in R v3.0.0 and bash were used extensively throughout the analysis.

For manuscripts utilizing custom algorithms or software that are central to the research but not yet described in published literature, software must be made available to editors and reviewers. We strongly encourage code deposition in a community repository (e.g. GitHub). See the Nature Research guidelines for submitting code & software for further information.

Data

Policy information about availability of data

All manuscripts must include a data availability statement. This statement should provide the following information, where applicable:

- Accession codes, unique identifiers, or web links for publicly available datasets
- A list of figures that have associated raw data
- A description of any restrictions on data availability

The meta-analysis data is up-loaded under dbGaP phs001349.v1.p1 (Meta-Analysis of Five Genome-Wide Association Studies of TGCT) and the TECAC replication data under phs001349.v2.p1 (NCI Testicular Germ Cell Tumors Post GWAS). UK Biobank data are available to all bona fide researchers upon data access application at http://www.ukbiobank.ac.uk/register-apply/. We obtained them under application number 3071 to Professor D. Timothy Bishop. The Icelandic population WGS data have been deposited at the European Variant Archive under accession code PRJEB15197. The ATACseq data and ENCODE data for PAINTOR analysis is included on a UCSC browser custom track at: https://genome.ucsc.edu/s/jpluta/TECAC2020.

Field-specific reporting

Please select the one below that is the best fit for your research. If you are not sure, read the appropriate sections before making your selection.

☒ Life sciences ☐ Behavioural & social sciences ☐ Ecological, evolutionary & environmental sciences

For a reference copy of the document with all sections, see nature.com/documents/nr-reporting-summary-flat.pdf

Life sciences study design

All studies must disclose on these points even when the disclosure is negative.

Sample size	Subjects were drawn from nine sources which represent all data currently available to the consortium (previous data from 5 TGCT studies; novel genotyped data; targeted genotype data; UK biobank; and deCODE genetics) for a total sample of 179,683 controls and 10,156 cases, comprising the largest association study of TGCT to date. The de novo genotype data alone was sufficiently powered to detect odds-ratios of approximately 1.12 or greater. Meta-analysis further increases statistical power.
Data exclusions	The replication data included 14,952 subjects. Exclusion criteria were pre-established, following the guidelines in Anderson et al., 2010. Subjects were excluded for discordant or ambiguous chromosomal sex, relatedness (IBD > 0.1875), excessive heterozygosity (> 3 standard deviations from the mean), low genotype call rates (<98%), non-European genetic ancestry. After quality in control, 10,608 subjects remained. Raw data included 312,960 genetic markers. SNPs were excluded for low genotype call rate (<99%), differential missingness by case status (p < 0.00001), differential missingness by DNA source (blood or saliva; p < 0.00001), Hardy-Weinberg equilibrium (p < 0.00001), duplicated physical position, and minor allele frequency < 0.01. SNPs with a MAF difference >10% between the two genotyping sites were removed. After quality control, 246,186 SNPs remained. Meta analysis of imputed data yielded 21,492,562. After meta-analysis, multi-allelic variants and SNPs demonstrating study heterogeneity (p<0.001) were removed, as well as variants that were not observed in all data sets, leaving a total of 16,511,202 SNPs.<br><br>The targeted genotyping dataset included 2,499 subjects and 46 SNPs. Subjects were removed for excess heterozygosity (> 3 standard deviation from the mean) and genotype missingness (≥10%). SNPs were screened for genotype missingness (> 2%), differential missingness (p< 0.001), and minor allele frequency (< 0.01). Afterquality control, 1,039 men with TGCT and 1,398 men without disease remained.
Replication	The 14,952 novel subjects used in this study were intended to replication previous findings of genetic associations with TGCT. The 60 top ranking previously unreported SNPs that were strongly associated (p<0.00005) with TGCT case status were selected for targeted genotyping, of which 46 passed in silico and initial quality testing for Fluidigm primer specificity. Replication results were meta-analyzed the targeted genotyping results and data from 7 other sources. Of the 56 previously reported variants, 12 failed to replicate.
Randomization	This study compared specific phenotypes (cancer versus non-cancer) which are predefined. Phenotype is the effect of interest, randomization is not applicable.
Blinding	This study compared specific phenotypes (cancer versus non-cancer) which are predefined. As there is no randomization, blinding is not applicable.

Reporting for specific materials, systems and methods

We require information from authors about some types of materials, experimental systems and methods used in many studies. Here, indicate whether each material, system or method listed is relevant to your study. If you are not sure if a list item applies to your research, read the appropriate section before selecting a response.

Materials & experimental systems

n/a	Involved in the study
☒	☐ Antibodies
☒	☐ Eukaryotic cell lines
☒	☐ Palaeontology and archaeology
☒	☐ Animals and other organisms
☐	☒ Human research participants
☒	☐ Clinical data
☒	☐ Dual use research of concern

Methods

n/a	Involved in the study
☒	☐ ChIP-seq
☒	☐ Flow cytometry
☒	☐ MRI-based neuroimaging

Human research participants

Policy information about studies involving human research participants

Population characteristics	Covariate-relevant covariates include: gender (all male cases, mostly male controls); diagnosis of testicular germ cell tumor for cases; no history of cancer for controls; genomic ancestry (restricted to European ancestry); geographic location at which subjects were sampled.
Recruitment	Participants were not recruited to the current study. Rather, participants were recruited to local case-control or cohort studies at TECAC centers, and contributed to the current study. A small number of control subjects were parents of cases that were not included in the study; although they did not have TGCT, these subjects may be at higher risk. The effect of including these controls is that point estimates may be slightly biased towards the null.
Ethics oversight	TECAC Steering Committee; University of Pennsylvania IRB

Note that full information on the approval of the study protocol must also be provided in the manuscript.
